# Moral judgment reloaded: a moral dilemma validation study

**DOI:** 10.3389/fpsyg.2014.00607

**Published:** 2014-07-01

**Authors:** Julia F. Christensen, Albert Flexas, Margareta Calabrese, Nadine K. Gut, Antoni Gomila

**Affiliations:** ^1^Psychology, Evolution and Cognition (IFISC-CSIC), University of the Balearic IslandsPalma, Spain; ^2^School of Psychology and Neuroscience, University of St AndrewsSt Andrews, UK; ^3^Strathclyde Institute of Pharmacy and Biomedical Sciences, University of StrathclydeGlasgow, UK

**Keywords:** moral dilemmas, moral judgment, decision making, cross cultural, DPHMJ

## Abstract

We propose a revised set of moral dilemmas for studies on moral judgment. We selected a total of 46 moral dilemmas available in the literature and fine-tuned them in terms of four conceptual factors (*Personal Force, Benefit Recipient, Evitability*, and *Intention*) and methodological aspects of the dilemma formulation (*word count, expression style, question formats)* that have been shown to influence moral judgment. Second, we obtained normative codings of arousal and valence for each dilemma showing that emotional arousal in response to moral dilemmas depends crucially on the factors *Personal Force, Benefit Recipient*, and *Intentionality*. Third, we validated the dilemma set confirming that people's moral judgment is sensitive to *all four* conceptual factors, and to their interactions. Results are discussed in the context of this field of research, outlining also the relevance of our RT effects for the Dual Process account of moral judgment. Finally, we suggest tentative theoretical avenues for future testing, particularly stressing the importance of the factor *Intentionality* in moral judgment. Additionally, due to the importance of cross-cultural studies in the quest for universals in human moral cognition, we provide the new set dilemmas in six languages (English, French, German, Spanish, Catalan, and Danish). The norming values provided here refer to the Spanish dilemma set.

“… but what happens when we are exposed to totally new and unfamiliar settings where our habits don't suffice?”

Philip Zimbardo (2007); The Lucifer Effect, *p. 6*

## Introduction

Moral dilemmas have become a standard methodology for research on moral judgment. Moral dilemmas are hypothetical short stories which describe a situation in which two conflicting moral reasons are relevant; for instance, the duty not to kill, and the duty to help. By inducing the participants to make a forced choice between these two reasons, it can be investigated which reason is given precedence in a particular situation, and which features of the situation matter for that decision. Accordingly, we assume that this kind of hypothetical “ticking bomb scenarios” can help to disentangle what determines human moral judgment. This is, however, only possible if the *moral dilemmas* are very well designed and potentially relevant factors are controlled for. The aim of this paper is to provide a set of such carefully designed and validated moral dilemmas.

The moral dilemmas commonly used in Cognitive Neuroscience experiments are based on what Foot (1967) and Thomson (1976) called the “Trolley Problem.” The trolley dilemma has two main versions. In the first one, a runaway trolley is heading for five railway workers who will be killed if the trolley pursues its course. The experimental participant is asked to take the perspective of a protagonist in the story who can choose the option to leap in and to pull a switch which will redirect the trolley onto a different track and save the five railway workers. However, redirected onto the other track, the trolley will kill one railway worker who would otherwise not have been killed. In an alternative version of the dilemma, the action the protagonist has to perform in order to stop the trolley is different. This time, there is no switch but a large stranger who is standing on a bridge over the tracks. The protagonist can now choose to push that person with his hands onto the tracks so that the large body stops the train. The outcome is the same: five individuals saved by sacrificing one. However, participants in this task more easily consent to pull the switch while they are much more reluctant to push the stranger with their own hands. The “action” that the protagonist of the story can choose to carry out—or not—is termed a *moral transgression* or *moral violation*. The choice itself, between the act of *committing* or *omitting* to carry out the moral transgression is a *moral judgment*. The decision to *commit* the harm is referred to as an *utilitarian* moral judgment, because it weights costs and benefits, while the decision to *refrain* from harm is a *deontological* moral judgment, because it gives more weight to the “not to kill” principle.

The influential work of Greene et al. (2001), which introduced moral dilemmas into Cognitive Neuroscience, has been followed by many other studies as a way to deepen our understanding of the role of *emotion* in moral judgment (for a review, see Christensen and Gomila, 2012). However, results obtained with this methodological approach have been heterogeneous, and there is a lack of consensus regarding how to interpret them.

In our opinion, one of the main reasons for this lays in the simple fact that the majority of studies have relied on the initial set of moral dilemmas devised by Greene et al. (2001). While this set indisputably provided invaluable evidence about the neural underpinnings of moral judgment, it was not validated. Thus, conceptual pitfalls and formulation errors have potentially remained unchallenged (Christensen and Gomila, 2012). In fact, one of the key findings that have been reported (i.e., emotional involvement in moral judgment) might have been due to uncontrolled variations in the dilemma formulations, rather than to the factors supposedly taken into account (i.e., personal vs. impersonal versions of the dilemma). As a matter of fact, Greene and colleagues themselves have worded constructive self-criticism with respect to that initial dilemma set and suggested using only a subset of the initial dilemmas, however, without validating them either (Greene et al., 2009). Still, researchers continue to use this initial set. Here we present our efforts to remedy this situation.

We have fine-tuned a set of dilemmas methodologically and conceptually (controlling four conceptual factors). The set was selected from previously used moral dilemma sets: (i) Greene et al. (2001, 2004) and (ii) Moore et al. (2008) (this set was based on Greene et al.'s but optimized). Both sets have been used in a wealth of studies, however, without previous validation (e.g., Royzman and Baron, 2002; Koenigs et al., 2007; Moore et al., 2008, 2011a,b). After the dilemma fine-tuning, norming values were obtained for each dilemma: (i) of arousal and valence (to ascertain the differential involvement of emotional processes along the dimensions of the 4 conceptual factors) and (ii) of moral judgment (to confirm that moral judgment is sensitive to the four factors)^1^. Finally, in the Supplementary Material of this work, we provide the new set in six languages: *English, French, Spanish, German, Danish*, and *Catalan* in order to make it more readily available for cross-cultural studies in the field. Please note that the norming study was carried out with the Spanish dilemma version. We encourage norming studies in the other languages (and in other cultures).

## Dilemma “fine-tuning”—proposal of an optimized set

All dilemmas included in this set involved the decision to carry out a moral transgression which would result in a better overall numerical outcome. The participant was always the protagonist of this action (the moral transgression)^2^ and all dilemmas involved killing (i.e., all social and other physical harm dilemmas were eliminated). Furthermore, of the initial 48 dilemmas, 2 were eliminated (the personal and impersonal versions of the cliffhanger dilemma) due to the unlikely acrobatics they involve.

In what follows we outline the changes we have made regarding (i) the *instructions* given to the participant (subsection *Instructions to the Participant*); (ii) the *dilemma design*, i.e., adjustment of *dilemma length, expression style*, etc. (subsection *Dilemma Design (1)—Formulation*), (iii) the *dilemma conceptualization*, i.e., thorough adaptation to the conceptual factors of *Personal Force, Benefit Recipient, Evitability*, and *Intentionality* (subsection *Dilemma Design (2)—Conceptual Factors*), and (iv) the formulation of the question eliciting the moral judgment (subsection *The Question Prompting the Moral Judgment*). In the end, we have produced 23 dilemmas with two versions each, one personal and one impersonal, 46 dilemmas in total.

### Instructions to the participant

To increase verisimilitude, we suggest that instructions at the beginning of the experiment ideally emphasize that participants are going to read short stories about difficult situations as they are likely to appear in the news or in the radio (for instance: “*in the following you will read a series of short stories about difficult interpersonal situations, similar to those that we all see on the news every day or may read about in a novel*”) (Christensen and Gomila, 2012, p. 14). This may help to put the participants “in context” for the task that awaits them. In addition, instructions could include a remark about the fact that participants will be offered one possible solution to the situation, and that their task will be to judge whether the proposed solution is acceptable, *given the information available* (such as: “*for each of the difficult situations a solution will be proposed. Your task is to judge whether to accept or not this solution”*). Indeed, the closure of options or alternatives is important. However, in previous dilemma sets, some dilemmas have included expressions such as “*the only way to avoid* [death of more people] *is to* [action proposal],” while other dilemmas did not. Whereas this is important information, including that same sentence in all dilemmas could make the reading rather repetitive and result in habituation. On the other hand, including it only in some dilemmas could bias participants' responses to these dilemmas with respect to the others. Therefore, we suggest presenting it only in the general instructions to the participants.

### Dilemma Design (1)—formulation

Control for formal characteristics of dilemma formulation includes:

#### Word count

Word count across dilemma categories: in the original sets the dilemmas were rather long. This can entail an excessively long experimental session, resulting in participant fatigue. In Moore et al. (2008) an effort was made to control for mean word count: the *Personal* moral dilemmas (PMD) had 168.9 words and *Impersonal* moral dilemmas 169.3 (IMD). The maximum word count of a dilemma was 254 and the minimum was 123. We shortened the dilemmas removing information that was not strictly necessary and equalized the expression style of personal and impersonal versions of each dilemma. For instance, technical terms and long, non-familiar words were removed. Now the first three sentences of each dilemma are almost the same for both versions of a dilemma (personal and impersonal). For instance, the English version of the new dilemma set has a mean word count of 130 words in the *Personal* and 135 in the *Impersonal* moral dilemmas. Our maximum number of words in a dilemma is 169 and the minimum 93. See the Supplementary Material for the word counts for each translation.

#### Framing effects

A framing effect consists in that people may judge one and the same situation differently, just because of the way it is described (Tversky and Kahneman, 1981; Petrinovich et al., 1993). Specifically, a clear risk of framing effects concerns the use of “kill” in some dilemmas, but “save” in others. People feel more inclined to choose inaction when *kill* is used, and more inclined toward action when *save* is emphasized (Petrinovich and O'Neill, 1996). To avoid this, in all dilemmas the words *kill* and *save* are used in the second paragraph where the participant is given the information about the proposed action (i.e., the moral transgression) and its consequences. Conversely, the words are removed from the question (e.g., *Rescue 911* scenario: instead of *Is it appropriate for you to kill this injured person in order to save yourself and everyone else on board?* the action verbs *throw* and *keep* were used). It is important to highlight the trade-off between cost (throw someone) and benefit (keep yourself and more people in the air) in the questions of all dilemmas. This was not accounted for in any of the previous dilemma sets.

#### Situational antecedents

In the original dilemma sets, keeping the situational antecedent used to present the characters constant was not accounted for. Thus, in the *Personal* version of the *Nuclear reactor* dilemma the situational antecedent could bias the participants' responses: *you are the inspector of a nuclear power plant that you suspect has not met its safety requirements. The plant foreman and you are touring the facility when one of the nuclear fuel rods overheats*… Later, it is this same foreman you are asked to consider to push into the fuel rod assembly. The participant was given knowledge about a badly kept nuclear plant, with an in-charge individual who did not bother to make the plant meet the safety requirements. This makes it easier to sacrifice the plant foreman to save the city than to sacrifice another, random, innocent person—which is the option to consider in all other dilemmas. Hence, prior information about the state of the power plant was removed, so that the foreman has no overt responsibility for the nuclear accident which is about to happen. Now he is a “random” person to be sacrificed, like in the other dilemmas. The *Nobel Prize* dilemma had a similar problem. A situational antecedent made the person in a position to be sacrificed (here, your *fellow researcher*) appear a greedy bad person, so that it may be easier to sacrifice him than another *innocent* fellow researcher. The dilemma was reformulated so that the fellow researcher appeared not to know that the potential buyers would use the invention as a weapon and only the protagonist explicitly knows it and is now again the only person with the possibility to prevent greater harm from happening. In total, four dilemmas were modified to keep constant the situational antecedents of the characters in the dilemmas.

#### Trade-off

Trade-off across dilemmas: previous sets mixed different kinds of moral transgressions, like stealing or lying. It is important not to mix them with killing, in order to avoid the risk of a non-desired carry-over effect between dilemmas. For instance, stealing, lying, or lack of respect, may elicit less severe judgments when *killing* is also present in other dilemmas of the set, than when it's not. Therefore, all dilemmas now raise the conflict between the option to *kill* a person in order to *save* a larger number of people, and the option of doing nothing and letting that larger number of people die.

#### Number of individuals

Number of individuals saved if the moral transgression is carried out: in the set there now are the following categories (i) 5–10 people, (ii) 11–50 people, (iii) 100–150 people and (iv) “thousands” of people or “masses” of people. It is an important variable to control for. A utilitarian response should become easier as more people are saved. Conversely, if moral judgment is purely deontological, the number of people saved is totally irrelevant. This is an interesting question to have as a working hypothesis. Using different amounts of “saved individuals” in the formulations of the dilemmas allows researchers to explore at which point the positive consequences outweigh the transgression required to obtain them. For instance, it has been shown that attachment (“closeness of relationship”) to the victim determines moral judgment more than the number of beneficiaries involved. Still, this question needs further research, once closeness is controlled for (Tassy et al., 2013). In this work, however, no specific analysis of this variable will be made, as it exceeds the limits of this norming study.

#### Information

Information supplied about the certainty of the consequences for the story character impersonated by the participant: in the Tycoon and Nobel Prize dilemmas it said that “*if you decide to* [action of the dilemma], *nobody will ever find out*.” This implies information about the future which cannot really be given with certainty, while at the same time contrasting with other stories where no such commitments about the future are made. This kind of information can bias moral judgment and confuse it with legal punishment (or its lack). Therefore, this information was removed altogether from the dilemmas. Similarly, dilemmas were excluded that cannot be understood without the assumption of an extraordinary ability or an unlikely event (such as the Cliffhanger)^3^.

### Dilemma Design (2)—conceptual factors

On the grounds of the literature about moral judgment (Christensen and Gomila, 2012), four main factors need to be controlled for in moral dilemma formulation: *Personal Force, Benefit Recipient* (who gets the benefit), *Evitability* (whether the death is avoidable, or not), and *Intentionality* (whether the harm is willed and used instrumentally or a side-effect).

#### Personal force

Initially, Greene et al. (2001, 2004) defined a *Personal* moral dilemma as one in which the proposed moral transgression satisfied three criteria: (i) the transgression leads to serious bodily harm; (ii) this harm befalls a particular person or group of people; and (iii) the harm is not the result of deflecting an existing threat onto a different party. Subsequently, Cushman et al. (2006) remarked that the crucial feature in a personal dilemma is whether physical contact between the victim and the aggressor is involved; a point also emphasized by Abarbanell and Hauser (2010), while Waldmann and Dieterich (2007) focused on the *Locus of Intervention* (focus on the victim or on the threat) as the difference between personal and impersonal dilemmas. Another proposal contended that the difference between *Personal* and *Impersonal* is whether the action is mechanically mediated or not (Royzman and Baron, 2002; Moore et al., 2008). In more recent work, Greene et al. have tried to offer an integrative definition (Greene, 2008; Greene et al., 2009). Specifically, these authors propose that a *Personal* moral transgression occurs when (i) the force that impacts the victim is generated by the agent's muscles, (ii) it cannot be mediated by mechanisms that respond to the agent's muscular force by releasing or generating a different kind of force and applying it to the other person, and (iii) it cannot be executed with guns, levers, explosions, gravity…

However, it seems as if this redefinition is driven by an effort to keep the interpretation of the initial results, which results in a circular argument: that “personal” dilemmas induce deontological judgments by emotional activation, while “impersonal” ones induce utilitarian judgments by rational calculation. Yet, it is not yet clear which aspect of the personal involvement influences moral judgment through emotional activation, nor is it clear which kind of moral relevance emotions elicited by one's involvement may have in the judgment. Similar considerations apply to the introduction of the distinction between “high-conflict” and “low-conflict” dilemmas (Koenigs et al., 2007), which also seem based on ex-post-facto considerations.

A principled way to clarify this distinction is in terms of the causal role of the agent in the production of the harm. What makes a dilemma impersonal is that the agent just initiates a process that, through its own dynamics, ends up causing the harm; while a dilemma is personal when the agent is required not just to start the action, but to carry it out by herself. According to this view, the presence of mediating instruments, by itself, does not make a dilemma personal or impersonal. It depends of the kind of active involvement of the agent they require and amounts to a difference in her responsibility of the caused harm, and in the resulting (felt) emotional experience of it. This can account for the different moral judgments to Personal and Impersonal Dilemmas, which are observed despite the fact that the same consequences occur. The best philosophical explanation of this difference is Anders (1962)'s reflection on the mass murders on the Second World War. He contended that these acts were made possible by the technical innovations that reduced the active involvement of soldiers in the killing to pushing a button to release a bomb. It is not just that the new arms were of massive destruction, but that their use was easier for us humans. Killing with one's hands is not just slower, but harder.

In the present dilemma set, the Personal dilemmas have been revised accordingly. Personal Moral Dilemmas now require that the agent is directly involved in the production of the harm. Impersonal Moral Dilemmas are those in which the agent is only indirectly involved in the process that results in the harm.

#### Benefit recipient

Self-interest is a well-known influence in moral judgments (Bloomfield, 2007). People will be more prone to accept an action whose consequences benefit themselves (i.e., the agent herself) than one that benefits others, maybe complete strangers. This “Self-Beneficial” vs. “Other Beneficial” contrast has been introduced more clearly in the revised set. We reformulated the *Modified Euthanasia* dilemma due to a confound in the trade-off specification. Therefore, as the dilemma had to be an *Other-beneficial* dilemma, now the key secret evidence the soldier could reveal if tortured is the location of a particularly important base camp (and not the camp of the protagonist's group).

#### Evitability

This variable regards whether the death produced by the moral transgression is described as *Avoidable* or *Inevitable*. Would the person “to be sacrificed” have died anyway (*Inevitable* harm), or not (*avoidable* harm)? Transgressions that lead to inevitable consequences are more likely to be morally acceptable, by the principle of lesser evil (Hauser, 2006; Mikhail, 2007). In the dilemma *Rescue 911*, a technical error in a helicopter puts the protagonist in the situation of having to decide to throw off one of her patients for the helicopter to lose weight. Without that sacrifice the helicopter would fall and everybody—*including that one patient*—would die. Conversely, the dilemma can also be formulated in such a way that the individual to be sacrificed otherwise would not have been harmed (*Avoidable* death), such as in the classical trolley dilemmas, where neither the bystander nor the innocent railway worker on the side track would have been harmed if the protagonist had not changed the course of events. This distinction has now been made more explicit in the dilemmas (for examples of work where this variable was discussed, see Moore et al., 2008; Huebner et al., 2011).

#### Intentionality

This factor refers to whether the harm is produced instrumentally, as something willed, or whether it happens as an unforeseen side-effect, as collateral damage, to an action whose goal is positive. This variable concerns the doctrine of the double effect that has been shown to be psychologically relevant (Foot, 1967; Hauser, 2006; Mikhail, 2007). Causing harm is more acceptable when it is produced as collateral damage, than when it is the goal of an action. Accordingly, *Accidental* harm refers to the case where the innocent victim of the dilemma dies as a non-desired *side effect* of the moral transgression that the protagonist carries out to save others. Conversely, *Instrumental* harm occurs when the protagonist intentionally uses the harm (i.e., the death) of the innocent victim *as a means* (i.e., instrumentally) to save the others.

The reformulation of the dilemmas and the fine-tuning according to this factor is particularly relevant and one of the main contributions of this norming paper. In the modified set of Moore et al. (2008), all *Personal* dilemmas were *Instrumental*, while *Impersonal* dilemmas included six *Instrumental* and six *Accidental*. The present set now allows a full factorial design including *intentionality*. To introduce *Accidental* vs. *Instrumental* harm in Personal dilemmas attention was paid to key aspects of the causal chain of the dilemma leading to the proposed salvation of the greatest number of people. First, the exact *intention* that the protagonist has in the very moment of committing the moral transgression was identified (*does she carry out an action with the intention to kill or not?*). Second, a differentiation was made between whether the harm is *directly* produced by the protagonist, or *indirectly* triggered by her action (do the positive consequences (the salvation of many) follow *directly* from the victim's death, or by some other event, an *independent mechanism* which was triggered by the protagonist's actions but not directly by her, nor directly willed by her?). The final point concerned by what means the larger number of people is saved (are they saved directly by the death of the victim, or for a different reason?).

**Figure 1:**
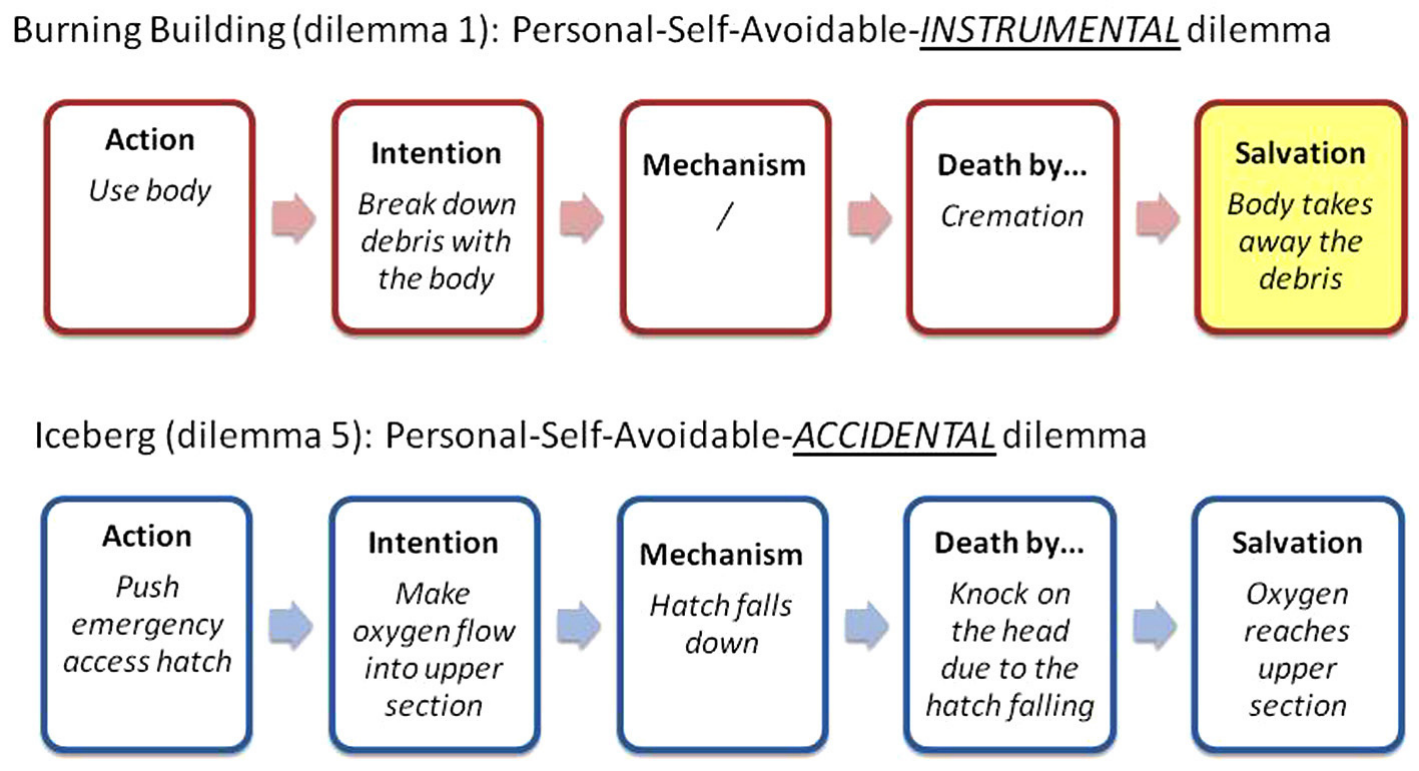
**Example of the causal chain of the proposed moral transgression that leads to the salvation**. In the *Instrumental version* of the Burning Building dilemma the proposed *action* is “to use the body of the victim.” The *intention* is “use the body to break down burning debris.” The victim *dies* directly by the fire and there is no independent *mechanism* in between. A larger number of people are saved due to the fact that the burning debris was eliminated *with the victim*. The harm to the victim was thus used *as a means* to save others. Said in different words, the body of the victim was literally used *instrumentally* with the intention to free the trapped group. Conversely, in the *Accidental version* of the Iceberg dilemma, the *action* of the protagonist is “to push the emergency access hatch.” The *intention* behind that action is “to make the oxygen flow to the upper section of the boat.” The victim dies *due to a knock on the head* by an independent *mechanism* which is the *falling down of the hatch*. Thus, the victim dies as a *side-effect* of the act of salvation that the protagonist carries out with the intention to get oxygen to the upper section of the boat.

Following this rationale, for a better comprehension of the *Intentionality* factor, the moral transgression is divided into a 5-part causal chain. This helps to disentangle the *Accidental*-*Instrumental* dichotomy (see Figure 1). The first thing to identify is the *action* by the protagonist (*what exactly does she do?*). Second, which is the exact *intention* behind that action (*why exactly does she do it?*)? Third, does the victim die by the intervention of some intermediate (and protagonist-*independent*) *mechanism* or is the death directly due to the action of the protagonist (*does she kill directly or by an independent mechanism?*)? Fourth, how does the innocent victim *die* (*how does she die?*)? Fifth, how is the larger number of people *saved* (*are they saved due to the death of the victim or for some other reason?*)?

**Figure 2:**
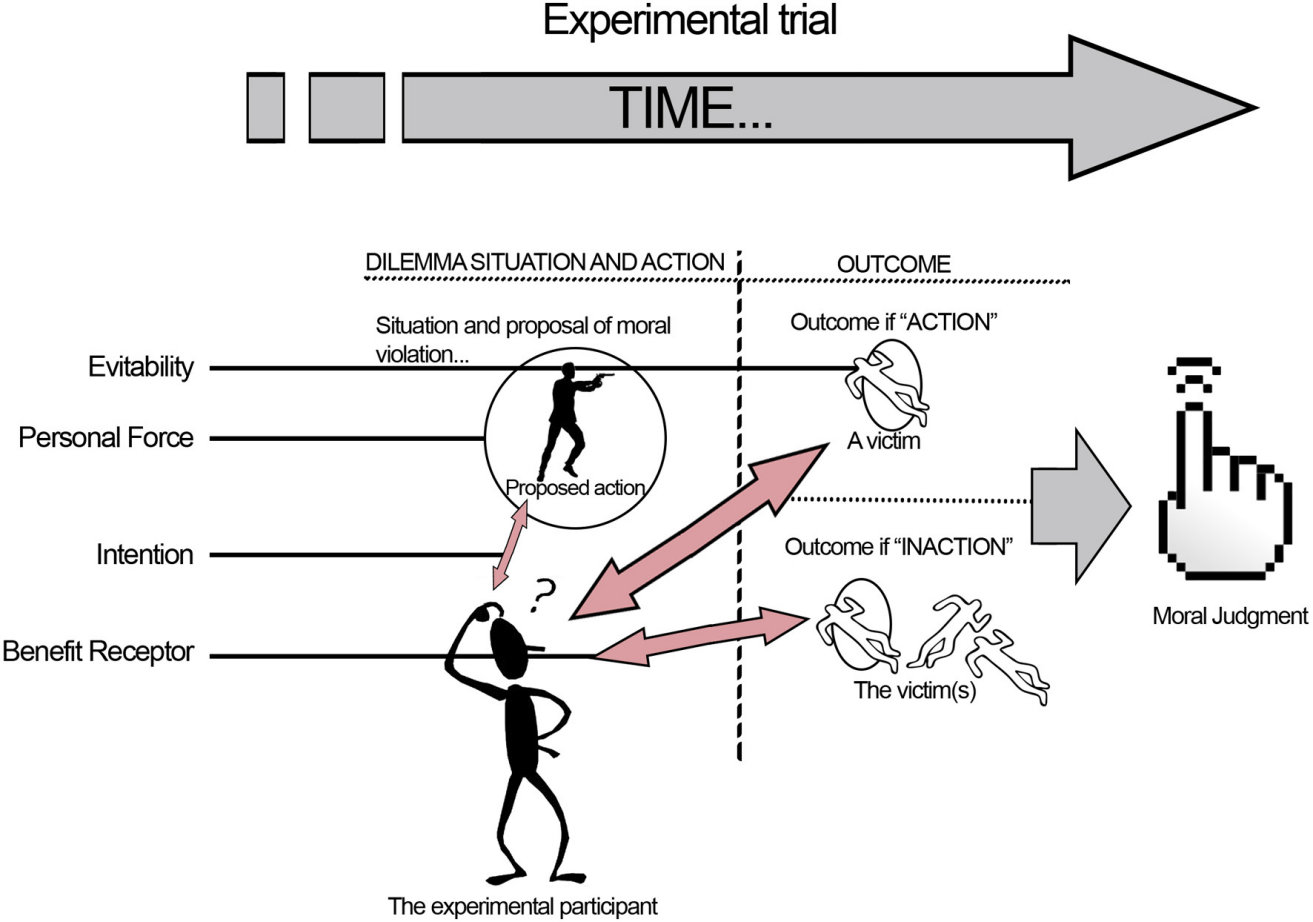
**The four factors in the dilemma set, adapted from Christensen and Gomila (2012), reproduced with permission**. (1) *Personal Force*: the kind of imaginary involvement with the situation: Personal, as direct cause, or Impersonal, as an indirect agent in the process of harm. (2) *Benefit Recipient*: concerns whether the protagonist's life is at stake (Self-Beneficial action), or not (Other-Beneficial action). (3) *Evitability*: regards whether the victim would die alongside the other individuals in the group if the moral transgression is not carried out (Inevitable death, the person would die anyway), or not (Avoidable death, the person would not die if no action is taken). (4) *Intentionality*: if the action is carried out intentionally with the explicit aim to kill the person as a means to save others, this is Instrumental harm (it explicitly *needs* the death of that person to save the others). If the innocent person dies as a non-desired side-effect of the action by some independent mechanism and not directly by the action of the protagonist, the harm is Accidental.

To summarize the four factors *Personal Force, Benefit Recipient, Evitability*, and *Intentionality*, the illustration in Figure 2 provides a schematic overview over how the four factors are presented to the participant during the course of a moral dilemma.

### The question prompting the moral judgment

The formulation of the final question to elicit the moral judgment after reading the dilemma has also given rise to some controversy. The problem of the influence that the type of question exerts on participant's moral judgments has been addressed empirically (e.g., O'Hara et al., 2010). Four question formats were used: *wrong, inappropriate, forbidden*, and *blameworthy* and found that people judged moral transgressions more severely when the words “wrong” or “inappropriate” were part of the formulation, than when the words “forbidden” or “blameworthy” were used. Another study found different behavioral effects following the questions *Is it wrong to…?* vs. *Would you?* (Borg et al., 2006). The question *Would you…?* resulted in faster RTs in judging moral scenarios as compared to judgments of non-moral scenarios, while the question *Is it wrong to…?* did not show any differences in RT comparing the moral to the non-moral condition. In view of these findings, it seems that deciding *what to do* is not processed in the same way as deciding whether an action is *right* or *wrong*, and that in moral dilemmas is the first that matters.

In recent work, two groups of researchers have addressed the related issue of whether “what we say is also what we do.” Thus, it was found that answering the question *Is it acceptable to…?* vs. the question *Would you…?* resulted in differential response tendencies (Tassy et al., 2013). However, another study showed that increasing the contextual information available to the participant resulted in more coherence between what they said they would do and what they actually did (Feldman Hall et al., 2012). In any case, it is clear that consistency is required.

For the present dilemma set a direct question was used *Do you [action verb] so that*… to emphasize the consequences of the choice made by the agent. Scales (Likert, Visual Analogical…) were used instead of a dichotomous answer format, as a way to uncover the degree of conflict experienced.

### Summary: the revised set

The revised set consists of 46 dilemmas, of which 23 are *Personal* and 23 are *Impersonal*. As can be observed in Table 1, we maintained the original dilemma numbers so that it is easy to compare across sets. In 23 of the 46 dilemmas, the protagonist's life is in danger and the moral violation results in saving not only a greater number of individuals, but also the protagonist herself (*Self-Beneficial* dilemmas), whereas in the remaining 23, the protagonist's life is not in danger (*Other-Beneficial* dilemmas). In turn, there are 11 *Personal* and 11 *Impersonal Self*-*Beneficial* dilemmas, and 12 *Personal* and 12 *Impersonal Other-Beneficial* dilemmas.

**Table 1:**
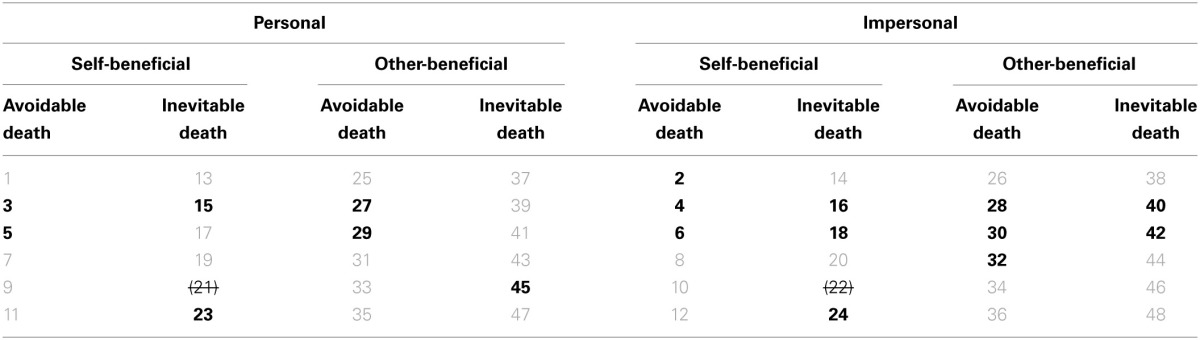
**Revised dilemmas**.

*The numbers refer to the dilemma number as given in the stimulus set (Supplementary Material). The colors refer to instrumental harm (gray) and accidental harm (black). We have kept the numbers (dilemma numbers) as in Moore et al. (2008) to facilitate comparisons between the sets. Please note that there is not the same number of dilemmas in each of the 16 categories. Please see our discussion of this matter in the section Arousal and Valence Norming Experiment on limitations. The dilemmas 21 and 22 were removed (Cliffhanger dilemmas, see in the section Dilemma Design (1)—Formulation for reference)*.

There are 24 dilemmas where the death is *Avoidable* and 22 where it is *Inevitable*. Finally, there are 18 dilemma scenarios with *Accidental* harm (7 *Personal* and 11 *Impersonal; 10 Self-Beneficial and 8 Other-Beneficial; 10 Avoidable and 8 Inevitable*) and 28 with *Instrumental* harm (16 *Personal* and 12 *Impersonal; 12 Self-Beneficial and 16 Other-Beneficial; 14 Avoidable and 14 Inevitable*). See Table 1 for a summary. Please note that it was not possible to provide the same number (quantity) of dilemmas in each of the 16 categories because we relied on the materials of the former set. Refer to our discussion of this matter in the Supplementary Material (A) on limitations.

## Arousal and valence norming experiment

Peoples' moral judgment has been shown to be sensitive to the affective impact of a dilemma in the individual (Moretto et al., 2010; Navarrete et al., 2012; Ugazio et al., 2012). However, no dilemma set has so far been assessed in terms of the affective arousal the individual dilemmas elicit in normal population as they are read—i.e., even if no moral judgment is required. Therefore, data points for affective arousal and valence were obtained for each dilemma of this set.

We know that peoples' moral judgments vary as a function of the four conceptual factors *Personal Force, Benefit Recipient, Evitability*, and *Intentionality*. However, how peoples' affective responses (valence and arousal) are modulated by these factors remains to be established. Besides, because inter-individual differences in emotional sensitivity and empathy can affect the subjective experience of arousal, participants in this experiment were assessed on these variables by means of self-report measures.

### Methods

#### Participants

Sixty-two undergraduate psychology students participated in this study in exchange for a course credit in one of their degree subjects (43 females, 19 males; age range = 18–48 years; *m* = 21.0, *SD* = 5.35). Participants completed four self-report measures. First, the *Interpersonal Reactivity Index* (IRI) (Davis, 1983), which has four scales that focus on perspective taking, tendency to identify with fictitious characters, emotional reactions to the negative experiences of others, and empathic concern for others. Second, the *Questionnaire of Emotional Empathy* (Mehrabian and Epstein, 1972) that conceives empathy as the vicarious emotional response to the perceived emotional experience of others. It explicitly understands empathy as different from Theory of Mind (ToM) and focuses on *emotional empathy* where high scores indicate a high responsiveness to other peoples' emotional reactions. Third, the *Questionnaire of Emotional Sensitivity* (EIM) (Bachorowski and Braaten, 1994), which refers to the intensity with which a person experiences emotional states irrespectively of their affective valence. Fourth, participants completed the *Toronto Alexithymia Scale* (TAS) in which a high score means difficulties in understanding and describing emotional states with words (Taylor et al., 1985). For results on the self-report measures, see Table 2. All were native Spanish speakers.

**Table 2:** **Participant characteristics in terms of emotional sensitivity, empathy, and alexithymia**.

**Instrument**	**Mean**	***SD***
EIM (Bachorowski and Braaten, 1994)	164.75	18.77757859
Emotional empathy (Mehrabian and Epstein, 1972)	46.7	25.71703913
IRI (Davis, 1983)	55.7	8.095405686
TAS (Taylor et al., 1985)	18.3	14.78237613

#### Materials

The forty-six moral dilemmas were arranged to be presented in random order in the stimulus presentation program DirectRT (www.empirisoft.com) v. 2006.2.0.28. The experiment was set up to run on six PCs [Windows XP SP3 PC (Intel Pentium Dual Core E5400, 2.70 GHz, 4 GB RAM)] and stimuli were displayed on 19″ screens (with a resolution of 1440 × 900 p; color: 32 bits; refresh rate: 60 Hz). Data were analyzed using the statistical package SPSS v. 18 (www.ibm.com).

#### Procedure

Participants signed up for the experiment in class after having completed the four self-report scales. The day of the experiment, participants provided demographic data regarding gender, age, and level of study. Informed consent was obtained from each participant prior to participation in any of the tasks and questionnaire procedures.

Participants were instructed as outlined in the section *Instructions to the Participant*. Each dilemma was presented in white Arial font, pt 16, on a black screen. By key press, the first paragraph of the dilemma appeared. With the next key press, the second paragraph appeared^4^. Participants read at their own pace, advancing from one screen to the other by pressing the space bar. With the third key press, the first two paragraphs of the dilemma disappeared and two Likert scales appeared on subsequent screens, the first asking participants to indicate their level of arousal (1 = not arousing at all; 7 = very arousing) and the second asking them to indicate the perceived valence of the dilemma (1 = very negative; 7 = very positive). The ratings were made by means of key press on the number keyboard of the computer. Four practice dilemmas were added in the beginning of the task. Data from these trials were discarded before data analysis.

The experiment was carried out in a laboratory of the university suited for experiments with six individual PCs separated in individual booths. Participants carried out the task in groups of 1–6 people. Viewing distance was of approximately 16 inches from the screen. The study was approved by the University's Ethics Committee (COBE280213_1388).

### Results

Factorial Repeated Measure (RM) 2 × 2 × 2 × 2 Analysis of Variances (ANOVA) were computed on subjective arousal and valence ratings (Likert scale data), and on the RT of the arousal ratings. The factors were (1) Personal Force (Personal vs. Impersonal harm); (2) Benefit Recipient (Self-Beneficial vs. Other-Beneficial); (3) Evitability (Avoidable vs. Inevitable harm); and (4) Intentionality (Accidental vs. Instrumental harm). As effect sizes we report Pearson's *r*, where 0.01 is considered a small effect size, 0.3 a medium effect and 0.5 a large effect (Cohen, 1988).

To rule out any effect of Gender in the results, the above ANOVA was computed with the between-subjects factor Gender. There was no effect of gender in any of the interactions with the four factors, neither in the arousal ratings: Personal Force^*^gender: *F*_(1, 60)_ = 1.47; *p* = 0.230; Benefit Recipient^*^gender: *F*_(1, 60)_ = 0.774; *p* = 0.383; *Evitability*: *F*_(1, 60)_ = 0.079; *p* = 0.780; Intentionality^*^gender: *F*_(1, 60)_ = 0.101, *p* = 752; nor in the valence ratings: *Personal Force* ^*^ *gender*: *F*_(1, 60)_ = 0.004; *p* = 0.949; Benefit Recipient^*^gender: *F*_(1, 60)_ = 0.346; *p* = 0.558; *Evitability*: *F*_(1, 60)_ = 0.019; *p* = 0.890; Intentionality^*^gender: *F*_(1, 60)_ = 0.184, *p* = 0.670, nor in the RT. Therefore, data of female and male participants were aggregated.

#### Arousal

All 16 dilemma categories were rated as being felt as of moderate to high arousal (range: *m* = 5.58–6.24; see Table 3). Two of the four factors showed significant effects on the arousal ratings. First, there was a significant main effect of *Personal Force* [*F*_(1, 61)_ = 6.031; *p* = 0.017; *r* = 0.30], *PMD* being rated as more arousing (*m* = 5.92; *SD* = 0.12), than *IMD* (*m* = 5.83; *SD* = 0.12). The second main effect was of *Benefit Recipient* [*F*_(1, 61)_ = 47.57; *p* < 0.001; *r* = 0.66], *Self-Beneficial* Dilemmas being rated as more arousing (*m* = 6.02, *SD* = 0.12) than *Other-Beneficial* Dilemmas (*m* = 5.70, *SD* = 0.13). See Figure S3. There were no significant main effects of *Evitability* [*F*_(1, 61)_ = 0.368; *p* = 0.546], nor of *Intentionality* [*F*_(1, 61)_ = 0.668; *p* = 0.417]. See Table S1 for the means and Figure S3 in the Supplementary Material.

**Table 3:** **RM ANOVA of the RT of the arousal ratings**.

**RM ANOVA—Main effects on RT AROUSAL (Personal Force, Benefit Recipient, Evitability, Intentionality)**					
	**Mean (ms)**	***SE***	***F-*test**	***p***	***r***
			**(1, 61)**		
**PERSONAL FORCE**					
Personal (PMD)	2564.46	112.96	5.796	0.019	0.36
Impersonal (PMD)	2716.77	123.19			
**BENEFIT RECIPIENT**					
Self-beneficial	2506.66	119.52	20.783	<0.001	0.88
Other-beneficial	2774.57	115.66			
**EVITABILITY**					
Avoidable	2648.79	116.83	0.085	0.771	ns
Inevitable	2632.44	117.71			
**INTENTIONALITY**					
Accidental	2623.86	118.10	0.258	0.613	ns
Instrumental	2657.37	119.02			

*The values represent miliseconds*.

There was a significant interaction of Benefit Recipient^*^Intentionality [*F*_(1, 61)_ = 15.24; *p* < 0.001; *r* = 0.44]. This indicates that *Intentionality* had different effects on participants' ratings of arousal depending on whether the dilemma was *Self-Beneficial* or *Other-Beneficial*. Figure S4 illustrates the results. Paired *t*-tests showed that when *Self-Beneficial Harm* was *Accidental* the dilemma was rated as more arousing than when it was *Instrumental* [*t*_(61)_ = 3.690, *p* < 0.001, *r* = 0.43]. For *Other-Beneficial Harm*, the pattern was reversed, as the *Instrumental Harm* dilemmas were more arousing than the *Accidental Harm* dilemmas [*t*_(61)_ = −1.878, *p* = 0.065, *trend effect, r* = 0.05]. When comparing the *Accidental* and *Instrumental Harm* conditions, we found that *Self-Beneficial, Accidental Harm* dilemmas resulted in higher arousal ratings than when dilemmas were *Other-Beneficial* [*t*_(61)_ = 7.626, *p* < 0.001, *r* = 0.49]. The same pattern emerged when the harm was *Instrumental*; it was judged as more arousing when it was *Self-Beneficial*, than when it was *Other-Beneficial* [*t*_(61)_ = 3.494, *p* = 0.001, *r* = 0.17]. If correcting for multiple comparisons using the Bonferroni method, this would mean accepting a new significance level of α = 0.05/4 → α^*^ = 0.0125. This should be taken into account when considering the result with the trend effect.

#### Valence

Descriptive statistics of the valence ratings confirmed that all 16 dilemma categories were rated as being of negative valence (range: *m* = 1.71–2.23; see Table S1).

There were significant main effects of *Personal Force* [*F*_(1, 61)_ = 28.00; *p* < 0.001; *r* = 0.57] and of *Benefit Recipient* [*F*_(1, 61)_ = 31.509; *p* ≤ 0.001; *r* = 0.58]. *PMD* were rated as significantly more negative (*m* = 1.905, *SD* = 0.065) than *IMD* (*m* = 2.054; *SD* = 0.068). Likewise, *Self*-*Beneficial* Dilemmas were rated as significantly more negative (*m* = 1.884, *SD* = 0.068) than *Other*-*Beneficial* Dilemmas (*m* = 2.075; *SD* = 0.067). The two other factors did not show main effects [*Evitability F*_(1, 61)_ = 1.201; *p* = 0.277; and *Intentionality F*_(1, 61)_ = 0.135; *p* = 0.715]. See Table S1.

There were two significant interactions. The first was Personal Force^*^Intentionality [*F*_(1, 61)_ = 7.695, *p* = 0.007; *r* = 0.33]. The Figure S5 shows that *Intentionality* had different effects on how people rated the valence of *PMD* and *IMD*. Paired *t*-tests showed that *Accidental* harm was rated as significantly more negative than *Instrumental* harm in *Impersonal* Moral dilemmas [*t*_(61)_ = −2.297, *p* = 0.025, *r* = 0.08], while no such difference was found between *Accidental* and *Instrumental* harm for *Personal* Moral dilemmas [*t*_(61)_ = 1.441, *p* = 0.155, *r* = 0.03]. See Figure S5. If correcting for multiple comparisons using the Bonferroni method, this would mean accepting a new significance level of α = 0.05/4 → α^*^ = 0.0125. This should be taken into account when considering the result of the first *t*-test (*p* = 0.025).

The second significant interaction was Benefit Recipient^*^Intentionality [*F*_(1, 61)_ = 6.041, *p* = 0.017; *r* = 0.30]. This indicates that intention had different effects on the valence ratings depending on whether the dilemma was *Self*- or *Other*-*Beneficial*. Paired *t*-tests showed that for *Self-Beneficial* Dilemmas, harm was judged significantly more negative when it was *Accidental* as compared to *Instrumental harm* [*t*_(61)_ = −2.300, *p* = 0.025, *r* = 0.08]. No such difference in valence ratings of *Accidental* and *Instrumental* harm for *Other-Beneficial* dilemmas [*t*_(61)_ = 1.296, *p* = 0.200, *r* = 0.03]. See Figure S6. If correcting for multiple comparisons using the Bonferroni method, this would mean accepting a new significance level of α = 0.05/4 → α^*^ = 0.0125. This should be taken into account when considering the result of these *t*-tests (*p* = 0.017 and *p* = 0.025).

The assessment of valence was only carried out to confirm that all dilemmas were of a strongly negative valence. This has hereby been confirmed and no other analysis will be carried out involving this feature of the dilemmas. All values for both arousal and valence are available for each dilemma in the excel spreadsheet that accompanies this manuscript (Supplementary Material).

#### Reaction time

A RM ANOVA was carried out on the RT of the arousal ratings with the factors *Personal Force, Benefit Recipient, Evitability*, and *Intentionality*. Main Effects were found for *Personal Force* and *Benefit Recipient*, no interactions were significant. See Table 3.

**Figure 3:**
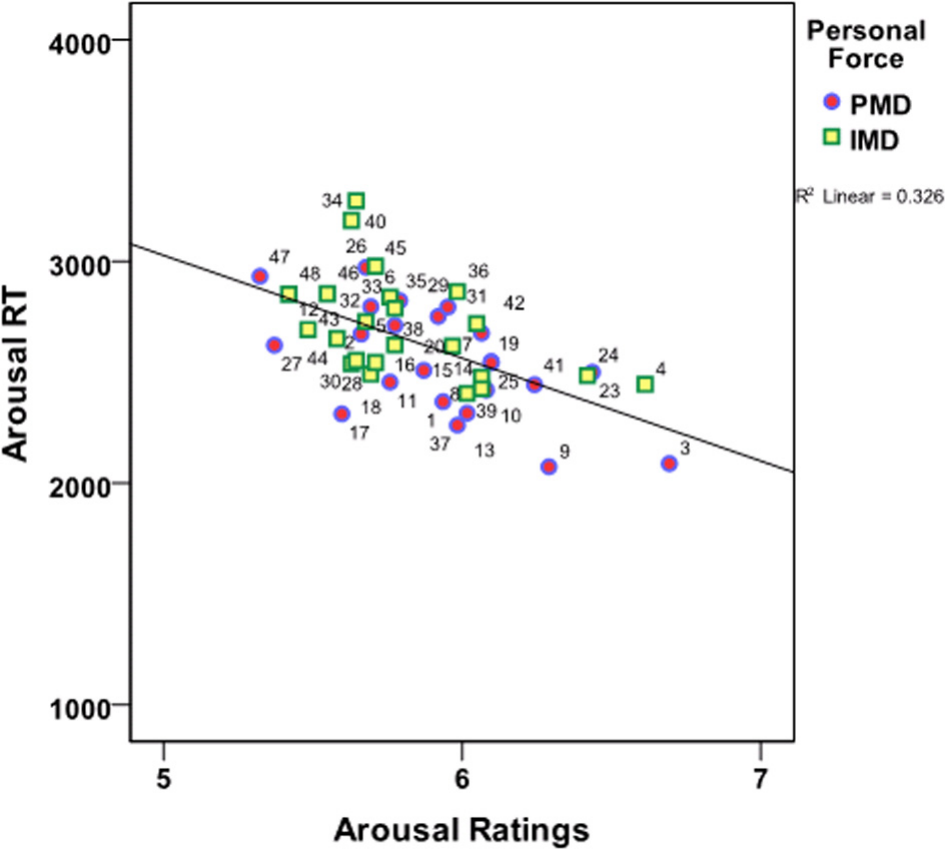
**Correlation between Arousal ratings and the RT**. Color coding: *Personal Moral Dilemmas* (PMD; Blue/Red, circles); *Impersonal Moral Dilemmas* (IMD; Green/Yellow, squares). Arousal ratings are 1 = Not arousing, calm; 7 = Very arousing, on the x-axis. RT is in milliseconds (ms) on the y-axis. The numbers refer to the dilemma numbers in the dilemma set.

Next, a regression analysis was conducted to ascertain how much of the variance in the RT of the arousal ratings was explained by the arousal ratings. This procedure was executed for each of the 16 dilemma categories. Summary Table 4 shows that except for four of the categories, the arousal ratings significantly explained between 6 and 38% of the variance in the RT. Figure 3 shows how the overall correlation between the variables indicates that the more arousing a dilemma was, the faster participants indicated their rating. The correlation coefficient between the mean arousal ratings and the mean RT of arousal ratings was, *p* < 0.001; *r* = −0.434.

**Table 4:** **Summary table of the regression analysis of arousal ratings as predictors of the arousal ratings' RT for each of the 16 dilemma categories**.

**Variables**	***B***	***SE B***	***R*^2^**	***B***	***P***
PMD_Self_Avo_Acc	−773.62	176.50	0.243	−0.493	0.000
PMD_Self_Avo_Instr	−336.08	134.03	0.095	−0.308	0.015
PMD_Self_Ine_Acc	−181.10	144.65	0.025	−0.160	0.215 (ns)
PMD_Self_Ine_Instr	−692.58	113.55	0.380	−0.619	0.000
PMD_Other_Avo_Acc	−130.67	150.71	0.012	−0.111	0.389 (ns)
PMD_Other_Avo_Instr	−231.73	143.76	0.042	−0.204	0.112 (ns)
PMD_Other_Ine_Acc	−276.63	136.91	0.062	−0.252	0.048
PMD_Other_Ine_instr	−495.32	140.80	0.171	−0.414	0.001
IMD_Self_Avo_Acc	−348.19	129.55	0.107	−0.328	0.009
IMD_Self_Avo_Instr	−582.35	126.31	0.261	−0.511	0.000
IMD_Self_Ine_Acc	−572.35	153.15	0.189	−0.435	0.000
IMD_Self_Ine_Instr	−382.88	174.58	0.074	−0.272	0.032
IMD_Other_Avo_Acc	−516.66	154.98	0.156	−0.395	0.002
IMD_Other_Avo_Instr	−486.55	150.54	0.148	−0.385	0.002
IMD_Other_Ine_Acc	−140.19	180.26	0.010	−0.100	0.440 (ns)
IMD_Other_Ine_Instr	−339.32	146.90	0.082	−0.286	0.024

*Abbreviations → IMD, Impersonal Moral Dilemmas; PMD, Personal Moral dilemmas; Self, Self-Beneficial; Other, Other-Beneficial; Avo, Avoidable; Ine, Inevitable; Acc, Accidental; Instr, Instrumental*.

#### Inter-individual differences: emotional sensitivity and empathy

To ensure that the results of our arousal ratings were not driven by inter-individual differences, participants had been assessed on a series of emotion-related questionnaires. Of the four questionnaires, the level of empathy measured with the questionnaire by Mehrabian and Epstein had a significant effect on arousal ratings and on arousal rating RT. The overall correlation coefficients for arousal ratings and Empathy scores was *r* = 0.289; *p* = 0.025 and for arousal RT and empathy scores it was *r* = −0.325; *p* = 0.011. The higher the empathy scores, the higher the arousal ratings to the dilemmas in general, and the shorter the RT (negative correlation coefficient).

### Summary: arousal and valence norming experiment

For a dilemma to be very negatively arousing (i.e., ratings very negative in valence and high in arousal), the proposed moral transgression had to be described as up-close and *Personal*. Besides, dilemmas where the protagonist's own life was at stake were perceived as more negatively arousing than those dilemmas where other peoples' lives were at stake. In particular, if the death of the innocent victim happened *accidentally* as a *non-desired side-effect*, the dilemma was perceived as more negatively arousing, specifically, if the protagonist's life was at stake, than if the accidental death of the victim happened in the intent to save other people. In detail:

#### Affective arousal and valence

- there were significant main effects of the factors *Personal Force* and *Benefit Recipient* both for arousal and valence ratings: *Personal* and *Self-Beneficial* dilemmas were perceived as more arousing and more negative than *Impersonal* and *Other-Beneficial* dilemmas, respectively;
- there were significant interactions between the two above factors and the factor *Intentionality*. *Intentionality* influences perceived arousal in such way that *Self-Beneficial* dilemmas (as compared to *Other-Beneficial* dilemmas) were rated as more arousing when harm happened as a non-desired side-effect (*Accidental* harm), while *Instrumental* harm (harm used as a means) was equally arousing in both *Self*- and *Other-Beneficial* dilemmas. Furthermore, when harm was *Personal* (up-close and corporal), as compared to *Impersonal* (distant and abstract), and used as a means (*Instrumental* harm), dilemmas were rated as more negative, than if harm was *Impersonal*. Conversely, participants found *Accidental* harm equally negative when it was *Personal* (up-close and corporal) and *Impersonal* (distant and abstract).

#### Reaction time

RT to a moral judgment task has previously been suggested as an indicator of emotional involvement. The more arousing a dilemma was, the faster participants were in making their rating.

#### Inter-individual differences

There was a correlation between inter-individual differences in empathy assessed by means of the *Questionnaire of Emotional Empathy* (Mehrabian and Epstein, 1972) and the arousal ratings. It showed that the higher the levels of empathy, the more arousing were the dilemmas to the participant. This makes sense because this instrument describes sensitivity to others' emotional states. It specifically conceives empathy as the vicarious emotional response to the perceived emotional experience of others and understands empathy as different from ToM and perspective-taking, and focuses on emotional empathy where high scores indicate a high responsiveness to other peoples' emotional reactions. However, apart from this correlation between arousal ratings and empathy level, no other individual differences had an effect on perceived arousal (the other variables we assessed were gender, IRI, emotional sensitivity, alexithymia). We therefore conclude that—at least in this sample of Spanish Undergraduates- the arousal ratings of this dilemma set are rather robust across individual differences.

### Discussion of arousal and valence norming experiment

While all dilemmas are rated similarly as negative in valence, significant differences were found in how they were rated in terms of felt arousal. This means, first, that at least part of the emotional involvement in moral judgment of the dilemmas can be due to the arousal triggered when reading the situational description. And second, results showed that differences in arousal are due to how the different conceptual factors are manipulated. Thus, Personal Force and Self-Beneficial dilemmas give rise to higher arousal ratings than Impersonal and Other-Beneficial ones. Prima facie this suggests that arousal has something to do with identification of the experimental participant with the perspective of the main character in the dilemmatic situation: it's when one feels more directly involved in the conflict, because of the action to be carried out or the consequences for oneself that the action will have, that one feels more aroused—even without having to make a judgment. However, this prima facie interpretion is too simplistic, for three reasons.

In the first place, it is clear that Personal Force dilemmas highlight the personal involvement in physically producing the harm. Similarly, Self-Beneficial dilemmas give rise to higher arousal ratings only when the harm produced is Accidental, rather than Instrumental. The latter case is one of self-interest: we experience less conflict when what's to be done is for our own benefit. Yet, it becomes difficult when a benefit cannot be produced without collateral damage. Third, whereas Self-Beneficial dilemmas take longer to be rated (than Other-Beneficial), Personal Force ones are rated faster than Impersonal ones. Jointly, these results suggest that arousal ratings can have several etiologies, and therefore cannot be interpreted simply as indication of degree of imaginary involvement with the situation or as a measure of experienced conflict. Both these causes need to be considered.

## Dilemma validation study—moral judgment experiment

To validate this moral dilemma set, a moral judgment task was set up to confirm the 4-factor structure in the dilemmas; i.e., the four conceptual factors *Personal Force, Benefit Recipient, Evitability*, and *Intentionality*.

Furthermore, to explore how the intentionality factor is understood by participants, two versions of the dilemma set were prepared: one version remained as has been described so far, while in the other the question eliciting the moral judgment included an “accidental harm specification” in the accidental harm dilemmas. For instance, in the dilemma Burning Building, the question is *Do you put out the fire by activating the emergency system, which will leave the injured without air, so you and the five other people can escape?* The sentence *which will leave the injured without air* is the accidental harm specification. This makes it clear to the reader the consequences of the proposed action. The analysis of this variable is included here, but in future researchers can choose to leave the accidental harm specification out of the question.

Additional analyses include (i) the analysis by Greene et al. (2001, 2004) that gave rise to the Dual Process Hypothesis of Moral Judgment (DPHMJ), (ii) an additional analysis of the *Intentionality* factor, and (iii) an analyses of how interindividual differences influence moral judgment.

### Methods

#### Participants

Forty-three undergraduate psychology and educational science students participated in this study in exchange for a course credit in one of their degree subjects (30 females and 13 males; age range = 18–54 years; *m* = 20.65, *SD* = 5.52). None of them had seen the dilemmas before. See Table 5 for participant characteristics including self-report measures of (i) the IRI (Davis, 1983), (ii) the *Questionnaire of Emotional Empathy* (Mehrabian and Epstein, 1972), (iii) the *Questionnaire of Emotional sensitivity* (EIM) (Bachorowski and Braaten, 1994), (iv) the TAS (Taylor et al., 1985), (v) the personality questionnaire *Big Five* (McCrae and Costa, 1999), and (vi) the Thinking Style Questionnaire, *Need For Cognition Scale* (NFC) (Cacioppo et al., 1984). All participants were native Spanish speakers.

**Table 5:** **Participant characteristics**.

**Instrument**	**Factors**	**Mean**	***SD***
EIM (Bachorowski and Braaten, 1994)		165	18.53
Emotional empathy (Mehrabian and Epstein, 1972)		48.58	23.41
IRI (Davis, 1983)		54.60	6.99
TAS (Taylor et al., 1985)		16.58	12.88
Big Five (McCrae and Costa, 1999)
	$|\begin{array}{l} \text{ Neuroticism}\\ \text{ Extraversion}\\ \text{ Openness to experience}\\ \text{ Agreeableness}\\ \text{ Consciencousness} \end{array}$	12.30	11.65
		21.58	9.70
		15.81	10.76
		13.72	9.84
		22.58	12.63
NFC (Cacioppo et al., 1984)		17.44	18.84

#### Materials

Forty-six standard moral dilemmas and four practice dilemmas were presented in random order with the stimulus presentation program DirectRT (www.empirisoft.com) v. 2006.2.0.28. The experiment was set up to run on six PCs (Windows XP SP3 PC (Intel Pentium Dual Core E5400, 2.70 GHz, 4 GB RAM) and stimuli were displayed on 19″ screens (with a resolution of 1440 × 900 p; color: 32 bits; refresh rate: 60 Hz).

#### Procedure

As in the previous experiment described in the section *Arousal and Valence Norming Experiment*. Additionally: after the second screen, the first two screens disappeared and the question appeared. The question eliciting the moral judgment was “*Do you [action verb] so that….”* A 7-point Likert scale was displayed below the question with the labels “*No, I don't do it*” under the number “1” and “*Yes, I do* it” under the number “7.” Half of the participants (22 participants) saw the question “*Do you [action verb] so that…,”* while the other half (21 participants) saw a question that furthermore involved the *Accidental* harm specification in the case of the *Accidental* harm dilemmas, such as in: “*do you [action verb] which will [mechanism that will lead to the death] so that…” (Type of Question)*. The ratings were made by means of key press on the using the number keys of the keyboard (top row of numbers) of the computer. Four practice dilemmas were added in the beginning of the task. Data from these trials were discarded before data analysis. The study was approved by the University's Ethics Committee (COBE280213_1388).

### Results

A factorial RM 2 × 2 × 2 × 2 ANOVA was computed with the Within-Subject factors Personal Force (PMD vs. IMD), Benefit Recipient (Self-Beneficial vs. Other Beneficial), Evitability (Avoidable vs. Inevitable harm), and Intentionality (Accidental vs. Instrumental harm). Question Type (with vs. without the Accidental harm specification) was the Between-Subject factor. As effect sizes we report Pearson's r, where 0.01 is considered a small effect size, 0.3 a medium effect and 0.5 a large effect (Cohen, 1988).

#### Subjective ratings: moral judgment

There was no significant main effect of the between-group factor *Type of Question* (with or without accidental harm specification) [*F*_(1, 41)_ = 0.164, *p* = 0.688] and there were no significant interactions between the Between-Subjects factor *Type of Question and* the four within-subject factors: Personal Force^*^Question Type [*F*_(1, 41)_ = 0.09; *p* = 0.766; *ns*]; Benefit Recipient^*^Question Type [*F*_(1, 41)_ = 0.296; *p* = 0.589; *ns*]; Evitability^*^Question Type [*F*_(1, 41)_ = 0.010; *p* = 0.921; *ns*]; Intentionality^*^Question Type [*F*_(1, 41)_ = 0.013; *p* = 0.911; *ns*]. This means that the two question formats (*with* and *without* the *Accidental* harm specification) are equivalent and do not affect the moral judgment a person makes. This means that the accidentality of the harm is understood from the narrative without the need to explicitly state it to the individual. Thus, data was aggregated for subsequent analyses.

There were significant main effects of all four Within-Subject factors: *Personal Force* [*F*_(1, 41)_ = 54.97; *p* < 0.001; *r* = 0.75]; *Benefit Recipient* [*F*_(1, 41)_ = 4.347; *p* = 0.043; *r* = 0.31]; *Evitability* [*F*_(1, 41)_ = 69.984; *p* < 0.001; *r* = 0.79]; and *Intentionality* [*F*_(1, 41)_ = 12.971; *p* = 0.001; *r* = 0.49]. Participants were less likely to commit harm in *PMD* (*m* = 4.069; *SD* = 0.124) than in *IMD* (*m* = 4.717; *SD* = 0.113) and they were more likely to commit a moral transgression to save themselves (*m* = 4.508; *SD* = 0.103), than to save others (*m* = 4.278; *SD* = 0.111). When the suggested harm was *Inevitable*, people were more likely to commit it (*m* = 4.633; *SD* = 0.124) than when harm was *Avoidable* (*m* = 4.152; *SD* = 0.103). Finally, when the death of the victim was *Accidental*, participants were more likely to commit the moral transgression (*m* = 4.549; *SD* = 0.125) than when it was *Instrumental* (*m* = 4.236; *SD* = 0.112). See Figures S7A–D.

Five of the six possible two-way interactions between the four factors were significant. See Table 6 for a summary of the means and interaction coefficients. Table 7 shows the *t*-tests to break down the interactions. Figure S8 summarizes the interactions graphically. If correcting for multiple comparisons using the Bonferroni method, this would mean accepting a new significance level of α = 0.05/4 → α^*^ = 0.0125 for breaking down each interaction. This should be taken into account when considering the result of the *t*-test in Table 7D (Self-Beneficial Accidental vs. Instrumental harm; *p* = 0.022).

**Table 6:** **Summary table of the interactions (dependent variable: moral judgment, Likert scale rating; range: 1;7)**.

	**Factors**		**Descriptives**		**Interaction coefficients**		
**Interactions**	**Factor 1**	**Factor 2**	**Mean**	***SD***	***F*_(1, 41)_**	***p***	***r***
Personal Force ^*^ Beneficiency	Personal	Self	4.076	0.142	18.248	<0.001	0.55
		Other	4.061	0.129			
	Impersonal	Self	4.939	0.139			
		Other	4.494	0.119			
Personal Force ^*^ Evitability	Personal	Avoidable	3.890	0.110	8.864	0.008	0.42
		Inevitable	4.248	0.147			
	Impersonal	Avoidable	4.415	0.112			
		Inevitable	5.018	0.123			
Personal Force ^*^ Intention	Personal	Accidental	4.326	0.141	14.582	<0.001	0.51
		Instrumental	3.812	0.131			
	Impersonal	Accidental	4.773	0.129			
		Instrumental	4.660	0.114			
Beneficiency ^*^ Evitability	Self	Avoidable	4.222	0.135	1.663	0.204	ns
		Inevitable	4.793	0.146			
	Other	Avoidable	4.082	0.110			
		Inevitable	4.474	0.132			
Beneficiency ^*^ Intention	Self	Accidental	4.416	0.137	40.202	<0.001	0.70
		Instrumental	4.599	0.140			
	Other	Accidental	4.683	0.146			
		Instrumental	3.872	0.118			
Evitability ^*^ Intention	Avoidable	Accidental	4.410	0.112	12.990	<0.001	0.49
		Instrumental	3.894	0.112			
	Inevitable	Accidental	4.689	0.151			
		Instrumental	4.577	0.121			

**Table 7:** **Follow-up *t*-tests to break down the interactions in the moral judgment task**.

	**Mean**	***SE***	***t-*test(42)**	***p***	***r***
(A) Tests to break down the interaction Personal Force^*^Benefit Recipient					
**PERSONAL MORAL DILEMMAS**					
Self-beneficient	4.076	0.142	0.134	0.894	ns
Other-beneficient	4.061	0.129			
**IMPERSONAL MORAL DILEMMAS**					
Self-beneficient	4.939	0.139	3.535	0.001	0.48
Other-beneficient	4.494	0.119			
(B) Tests to break down the interaction Personal Force^*^Evitability					
**PERSONAL MORAL DILEMMAS**					
Avoidable	3.890	0.110	−4.742	<0.001	0.59
Inevitable	4.248	0.147			
**IMPERSONAL MORAL DILEMMAS**					
Avoidable	4.415	0.112	−9.159	<0.001	0.82
Inevitable	5.018	0.123			
(C) Tests to break down the interaction Personal Force^*^Intentionality					
**PERSONAL MORAL DILEMMAS**					
Accidental	4.326	0.141	4.681	<0.001	0.59
Instrumental	3.812	0.131			
**IMPERSONAL MORAL DILEMMAS**					
Accidental	4.773	0.129	1.265	0.213	ns
Instrumental	4.660	0.114			
(D) Tests to break down the interaction Benefit Recipient^*^Intentionality					
**SELF-BENEFICAL DILEMMAS**					
Accidental	4.416	0.137	−2.397	0.021	0.35
Instrumental	4.610	0.140			
**OTHER-BENEFICAL DILEMMAS**					
Accidental	4.683	0.146	5.605	<0.001	0.65
Instrumental	3.872	0.118			
(E) Tests to break down the interaction Evitability^*^Intentionality					
**AVOIDABLE HARM**					
Accidental	4.411	0.112	5.853	<0.001	0.67
Instrumental	3.894	0.112			
**INEVITABLE HARM**					
Accidental	4.689	0.151	0.977	0.334	ns
Instrumental	4.578	0.121			

First, the *Benefit Recipient* variable had a differential effect on the moral judgment for *PMD* and *IMD* (Figure S8A). Participants were more likely to commit harm if the harm was carried out to safe themselves (*Self*-*Beneficial*, as compared to *Other*-*Beneficial*), however, only if the dilemma was *Impersonal*. If harm was *Personal*, participants were equally likely to commit the harm both when it was *Self*- or *Other-Beneficial*.

Second, also the *Evitability* variable had a differential effect on the moral judgment for *PMD* and *IMD* (Figure S8B). Participants made more deontological responses for PMD in general; however, they were more likely to commit harm when the death of the innocent person was *Inevitable* (as compared to *Avoidable*).

Third, also the *Intentionality* variable affected how participants judged *PMD* and *IMD* (Figure S8C). Again participants were overall more likely to make a deontological moral judgment in PMD than in IMD, however, participants were less likely to commit the moral transgression when harm was *Instrumental* (as compared to *Accidental*), but specifically only in the case of *PMD*.

Fourth, the *Intentionality* variable affected how participants judged *Self*- and *Other*-*Beneficial* dilemmas (Figure S8D). If the proposed harm was *Instrumental*, participants were less likely to commit it when the dilemma involved harm toward *Others* (as compared to harm toward the participant herself), while for accidental harm, participants were less likely to commit harm if it was accidental to save herself, than if it was to save others.

Fifth, *Intentionality* also affected how participants judged *Avoidable* and *Inevitable dilemmas* (*Evitability* factor), (Figure S8E). When harm was *Avoidable* (as compared to *Inevitable*), participants were less likely to commit it when the harm described in the dilemma was *Instrumental* than when it was *Accidental*. However, participants were equally likely to commit harm to both *Accidental* and *Instrumental* harm dilemmas when the harm described in the dilemma was *Inevitable*.

That there was no interaction between *Benefit Recipient* and *Evitability* means that participants were equally likely to commit harm, irrespective of whether death was *Avoidable* or *Inevitable* for *Self-* or *Other-Beneficial* dilemmas.

#### Reaction time

There was one significant main effect [*Intentionality*: *F*_(1, 41)_ = 13.252; *p* = 0.001; *r* = 0.49] and one significant interaction [Intentionality^*^Question Type: F_(1, 41)_ = 13.629; *p* = 0.001; *r* = 0.50]. Participants in general needed longer to make moral judgments about actions involving *Accidental* harm (*m* = 5803.223; *SD* = 424.081) than of actions involving *Instrumental* harm (*m* = 5185.185; *SD* = 394.389). The interaction indicates that *Intentionality* had a differential effect on RT depending on the *Question Type*. The group that had the question *with* the *Accidental* harm specification, needed significantly longer to respond to *Accidental* harm (*m* = 6356.081; *SD* = 578.441) than the group *without* such specification (*m* = 5250.365; *SD* = 620.309). No such difference appeared between the groups for *Instrumental* harm (*m* = 5112.582; *SD* = 537.941 and *m* = 5259.065; *SD* = 576.878, respectively).

Due to the fact that the only main effect and interactions that appear significant in the analysis of the RT data is the factor that regards the Between-Subject variable *Type of Question*, this effect was explored more closely. Therefore, the RM ANOVA was computed again, first with the participants in the *With* condition and afterwards with the participants in the *Without* condition. Again the factor *Intentionality* was significant in the *With* condition [*F*_(1, 22)_ = 21.208; *p* < 0.001; *r* = 0.70], but not in the *Without* condition [*F*_(1, 19)_ = 0.002; *p* = 0.964]. Hence, the effect was merely driven by the higher number of words in the questions in the *With* condition.

To ensure that RT was not conditioned by the word count of the questions in general, a regression was computed with word count in the question as a predictor and RT as the dependent variable. No significant relationship was found (*B* = −27.695; *B*_*SD*_ = 30.711; β = −0.234; *p* = 0.382). Hence, the word count of the questions did not influence the RT of participants except in this particular case of the *Intentionality* factor. Apart from this problematic effect, there were no other significant main effects or interactions.

**Figure 4:**
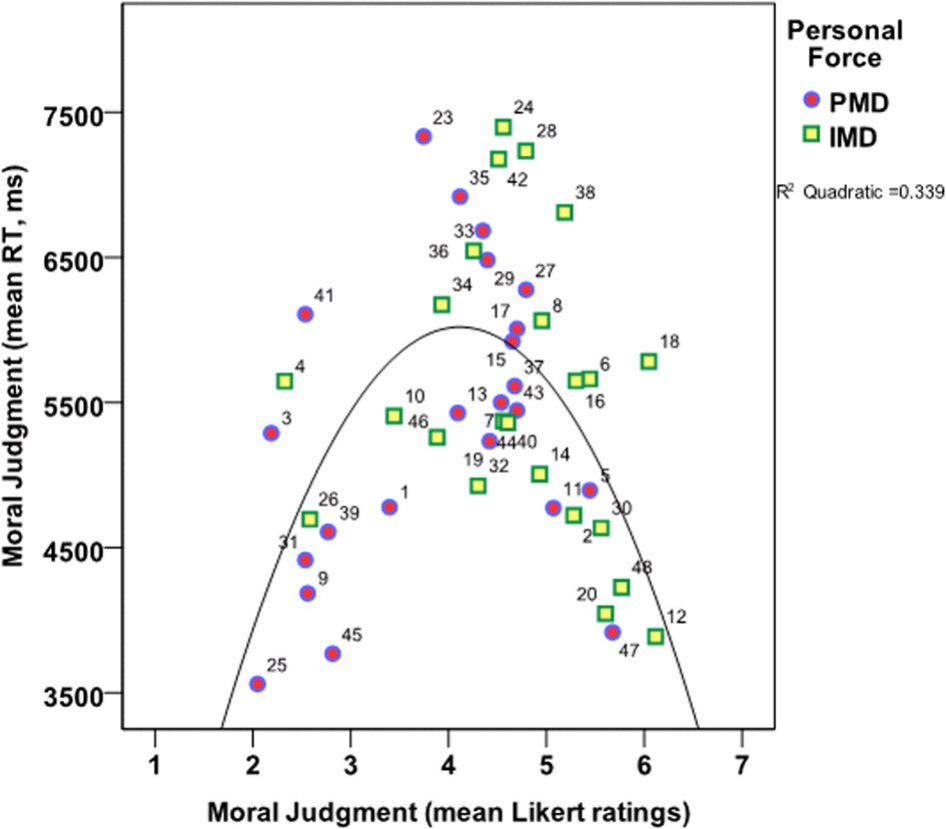
**Curvilinar relationship between *Moral Judgment* and RT**. Color coding: *Personal Moral Dilemmas* (Blue/Red, circles); *Impersonal Moral Dilemmas* (Green/Yellow, squares). Mean Likert scale responses: 1 = *No, I don't do it*, i.e., *deontological* moral judgment; 7 = *Yes, I do it*, i.e., *utilitarian* moral judgment. RT is in milliseconds (ms). PMD, Personal Moral Dilemmas; IMD, Impersonal Moral Dilemmas.

As much research in the field of moral judgment with moral dilemmas suggests a relation between the type of moral judgment (deontological *vs*. utilitarian) and RT, this matter was explored further. First, a curvilinear regression was computed with *Moral Judgment* as predictor and the RT as dependent variable. The resulting model was significant [*F*_(1, 41)_ = 11.015; *p* < 0.001; *r* = 0.46] and moral judgment accounted for 33.9% of the variance in the RT. Both for very deontological (Likert ratings toward 1) and very utilitarian moral judgments (Likert ratings toward 7) participants were faster than when making a more intermediate moral judgment (Likert ratings around 4). See the illustration of the relation between moral judgment and RT in Figure 4.

To assess RT as a function of the response given (deontological vs. utilitarian in absolute terms, not in a scale from 1 to 7 as presented above) as in Greene et al. (2001, 2004), the Moral Judgment values of the 7-point Likert scale were dichotomized. Judgments of values between 1 and 3 were considered “deontological,” and values between 5 and 7 were considered “utilitarian.” Values of 4 were discarded. Mean RT was calculated as a function of this re-coding. Subsequently, the ANOVA from Greene et al. (2001, 2004) 2 × 2 (*Response Type* and *Personal Force*) was carried out. No significant main effects were found [*Response Type*: *F*_(1, 42)_ = 0.402; *p* = 0.529; *Personal Force*: *F*_(1, 42)_ = 0.197; *p* = 0.659].

In previous analyses, the factor *Intentionality* has been shown to be of key relevance in moral judgment. Therefore, another 2 × 2 ANOVA with the variables *Response Type* and *Intentionality* was run. There was a significant main effect of *Intentionality* (*p* = 0.015) and a significant interaction of Response Type^*^Intentionality (*p* = 0.018), see Table 8 and Figure S9. Breaking down the interaction it was shown that participants took longer to make a deontological moral judgment when harm was then produced *accidentally*, than if it was *instrumental* (*p* = 0.003). No such difference was found for utilitarian moral judgments (*p* = 0.681), see Figure S9.

**Table 8:** Main Effects and Interactions of the RM ANOVA Question Type^*^Intentionality.

**(A) RM ANOVA—Main effects on RT MORAL JUDGMENT as a function of RESPONSE TYPE**					
	**Mean**	***SE***	***F*_(1, 41)_**	***p***	***r***
**QUESTION TYPE**					
Deontological response	5680.779	427.726	0.005	0.946	ns
Utilitarian response	5661.827	441.793			
**INTENTIONALITY**					
Accidental harm	6009.467	449.472	6.499	0.015	0.37
Instrumental harm	5333.139	415.105			
**INTERACTIONS**					
Response Type^*^Intentionality			6.010	0.018	0.65
**(B) Tests to break down the interaction**					
	**Mean**	***SE***	***t-*test(42)**	***p***	***r***
**DEONTOLOGICAL RESPONSE**					
Accidental harm	6434.148	571.955	3.313	0.003	0.46
Instrumental harm	4927.411	393.270			
**UTILITARIAN RESPONSE**					
Accidental harm	5584.787	424.480	−0.414	0.681	ns
Instrumental harm	5738.867	528.586			

*Mean Likert scale responses: 1 = No, I don't do it, i.e., deontological moral judgment; 7 = Yes, I do it, i.e., utilitarian moral judgment. RT is in milliseconds (ms)*.

#### Inter-individual differences: gender

There was a significant interaction between the factor *Benefit Recipient* and the participants' gender [*F*_(1, 61)_ = 10.079; *p* = 0.003; *r* = 0.37]; male participants were more ready to commit a harm in the case of *Self*-*Beneficial* dilemmas (*m* = 5.137; *SD* = 0.215), than female participants (*m* = 4.235; *SD* = 0.142). In the *Other-Beneficial* dilemma category, no such gender differences were found (males: *m* = 4.439; *SD* = 0.203; females: *m* = 4.208; *SD* = 0.133). This effect is reported for the sake of completeness of the scientific record. However, first, we did not specifically contemplate this effect, so we did not have equal numbers of male and female participants. Second, we do not aim to make any assumptions about gender differences based on such preliminary data. There is no sound scientific evidence that supports why there should be gender differences in moral judgment, nor of what kind these may be, nor what should be the evolutionary basis for them. This is a sensitive issue that deserves thorough investigation that goes far beyond the scope of this paper. Therefore, we assume that there are no genuine gender differences in moral judgment in participants of one same culture and have chosen to analyze the data of female and male participants together.

Two other studies have reported an effect of gender in their data (Fumagalli et al., 2009, 2010). However, the dilemma set used in these studies was the originally used by Greene et al. (2001, 2004) which has important methodological shortcomings (as pointed out by this paper; for a review see Christensen and Gomila, 2012), which is why ideally such claims on gender differences should really not be made. For such claims to be based on solid grounds a study should be designed controlling variables of empathy and other personality factors between genders, and of course, have an equal sample size of each gender.

#### Inter-individual differences: thinking style, personality traits, emotional sensitivity

To test the influence of inter-individual differences on moral judgment a regression was computed with all of the scores of the questionnaires assessing inter-individual differences in the model predicting the mean moral judgment of the participants. As shown in Table S2, the resulting regression model was significant [*F*_(10)_ = 2.954; *p* = 0.011; *r* = 0.47] and explained 50.5% of the variance in the moral judgments. However, only three of the 10 predictor variables were significant: *Emotional Sensitivity* (*p* = 0.018), and two of the Big Five factors, *Agreeableness* (*p* = 0.046) and *Conscientiousness* (*p* = 0.001). The higher the scores in the *EIM*, the more deontological were the moral judgments (participants with higher scores in the *EIM* were less susceptible to commit the proposed harm). For the two factors of the Big Five, the pattern was reverse: the higher the scores, the more utilitarian were the judgments (participants with higher scores in these two dimensions were more likely to commit the proposed harm). However, considering the *Beta* coefficient, it can be observed that these effects were—although existent—rather small.

### Arousal and moral judgment

**Figure 5:**
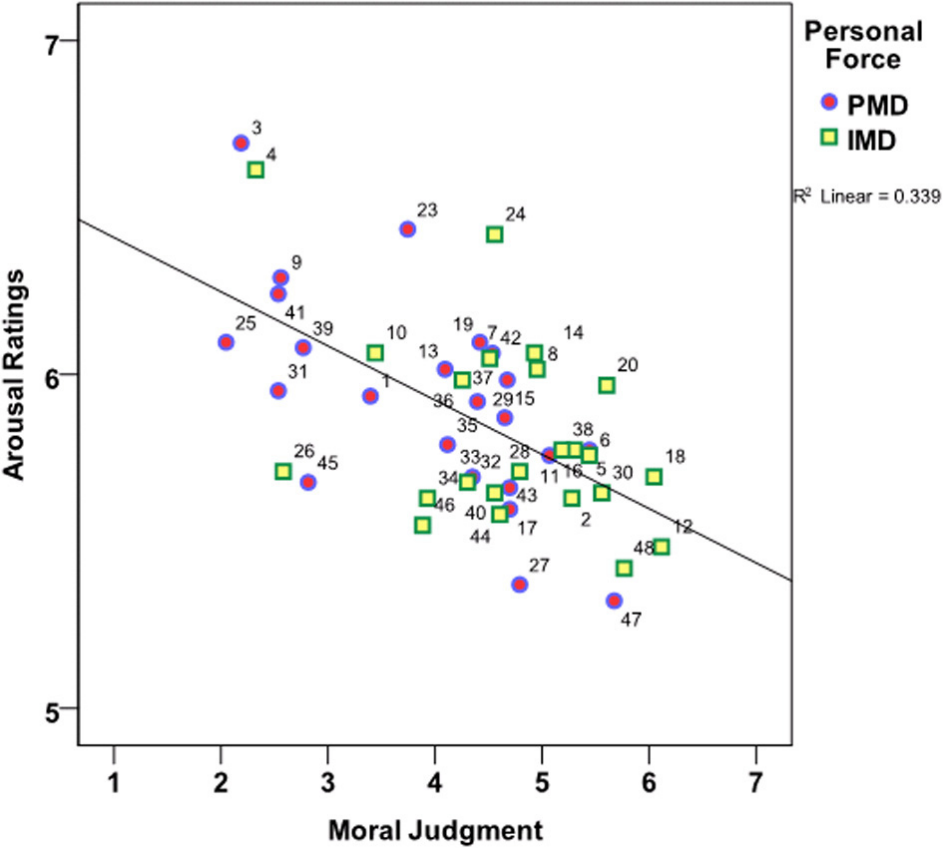
**Relationship between level of arousal of a dilemma and the moral judgment made to that dilemma. Color/shape coding: *Personal Moral Dilemmas* (Blue/Red, circles); *Impersonal Moral Dilemmas* (Green/Yellow, squares)**. Mean Likert scale responses: 1 = *No, I don't do it*, i.e., *deontological* moral judgment; 7 = *Yes, I do it*, i.e., *utilitarian* moral judgment. Mean Arousal scale responses: 1 = *Not arousing, calm*; 7 = *Very arousing*.

In order to determine whether the levels of arousal of the dilemmas rated by one group of participants, would be related to the moral judgments of a different group of participants, the dataset was transposed and dilemmas treated as cases. A simple regression was conducted with the arousal ratings as predictor variable and the moral judgments as dependent variable. The resulting model was significant [*F*_(1, 44)_ = 22.613; *p* < 0.001; *r* = 0.58], showing that the level of arousal of a dilemma predicted 33.9% of the variance in the moral judgment variable. Figure 5 shows that the more arousing a dilemma was, the more likely participants were to refrain from action (i.e., not committing the moral transgression). See Table S3 for the model parameters.

### Summary: moral judgment experiment

With this fine-tuned set of moral dilemmas it was confirmed that the four factors *Personal Force, Benefit Recipient, Evitability*, and *Intention*ality determined participants' moral judgment:

First, participants tended to exhibit a deontological response style (i.e., they refrained from committing harm) when harm was described as *Personal* (as compared to *Impersonal*), *Other-Beneficial* (as compared to *Self -Beneficial*), *Avoidable* (as compared to *Inevitable*), and *Instrumental* (as compared to *Accidental*). In other words, when harm was abstract and spatially and intentionally separated from the agent, participants were more likely to commit this moral transgression than if the harm was described as up-close and gave an impression of “bloody hands.”

Second, participants more readily sacrificed the life of another person if their own life was at stake than if the moral transgression would merely save other people. Besides, if harm to the victim would have happened anyway, irrespective of whether the moral transgression was carried out by the agent or not (as in “*or one person of 5 is killed or they all die*”), participants were more likely to incur in the moral transgression.

Third, participants more readily committed harm if harm happened as a non-desired side-effect of the action of the agent, it was more readily committed by the participants than if the proposed harm would result in using the death of the victim as a means to salvation of the others.

As regards the interactions between the factors:

First, the interaction between *Personal Force* and *Benefit Recipient* indicated that participants were equally likely to commit a moral transgression when the proposed harm involved “bloody hands,” both when the harm would result in salvation of oneself or of others. However, when the proposed harmful action was abstract and distant, participants made a difference in their moral judgment, depending on whether the salvation regarded themselves or others. Abstract harm commission made a utilitarian response more likely when it was executed to save themselves.

Second, the interaction between *Personal Force* and *Intentionality* indicated that harm that happened as a non-desired side-effect of the moral transgression was consented equally in IMD, both when harm was accidental and when it was instrumental. However, in PMD, if harm was used as a means (instrumentally), this made participants' moral judgments more deontological than when harm was accidental.

Third, the interaction between *Benefit Recipient* and *Intentionality* indicated that for Self-Beneficient Dilemmas, when harm happened as a non-desired side-effect of the proposed action, participants were less likely to commit the moral transgression, than when it was instrumental. Conversely, when the harm would benefit others, the pattern was reverse: more deontological moral judgments when harm was instrumental, than when it was accidental.

Fourth, the interaction between *Personal Force* and *Evitability* indicates that for both IMD and PMD, avoidable harm resulted in more deontological moral judgments than did inevitable harm.

Fifth, the interaction between *Evitability* and *Intentionality* indicates that both when harm to the victim could have been avoided, harm as a side-effect was more readily consented, than was the use of harm as a means. For inevitable harm no such difference between accidental and instrumental harm commission was found.

Furthermore, we found that the more arousing a dilemma was, the more likely it was that participants would choose a deontological response style.

Finally, there was no main effect of *Type of Response* found by Greene et al. (2001, 2004), indicating that with this optimized dilemma set deontological responding is not faster than utilitarian responding. Neither was there an interaction between Type of Response^*^Personal Force. However, with an additional ANOVA with the factors *Type of Response* and *Intentionality* it was shown that there was a significant main effect of *Intentionality*. Yet, more importantly, there was an interaction between *Type of Response* and *Intentionality*. This indicates that for dilemmas people were judging deontologically, it took them particularly long to make that judgment in the case when the proposed harm would result in accidental harm to the victim.

### Discussion of the moral judgment experiment

Summing up, results here show that that we are more prone to behave for our benefit, if the harm will take place in any case and producing the harm is not very demanding. Conversely, we are going to experience a conflict—indexed by a longer response—when we are forced to do the harm ourselves, or to do harm as collateral damage to benefit others. Moral principles can be broken but only in well-justified situations (when consequences are “big enough”). It's not that we are deontological or utilitarian thinkers, we are neither: moral judgments are better viewed from the point of view of casuistics, the particularist approach to morals that takes the details of each case into account. Any small detail may matter to our moral judgment. Results show, in any case, that rules are not applied algorithmically or in a strict order (Hauser, 2006).

## Overall discussion

Apart from providing normative values of valence, arousal, moral judgment and RT for 46 moral dilemmas^5^, the results of this dilemma validation study challenge the DPHMJ proposed by Greene et al. (2001, 2004). According to this hypothesis, deontological moral judgments (refraining from harm) are fast and emotion-based, while utilitarian moral judgments (deciding to commit the harm) are slow as a result of deliberate reasoning processes. The assumptions of the DPHMJ were based on a reaction time finding where an interaction between the *Type of Response* given (deontological vs. utilitarian) and the *Personal Force* (Personal vs. Impersonal) showed that when harm was consented in a *Personal* Moral Dilemma (utilitarian response), RT was significantly longer than when harm was not consented (deontological response). No such difference in the response time was found for *Impersonal* Moral Dilemmas. However, in our study, while we also found that higher arousal correlates with deontological judgment (in line with Moretto et al., 2010), we failed to find the relationship with RT: both deontological and utilitarian decisions can be made equally fast, and both to personal and impersonal dilemmas, depending on the other factors involved. To put it another way, a fast judgment takes place when, either a deontological reason guides the judgment, or when utilitarian considerations clearly dominate. Therefore, while we agree that the dilemmas that take longer are those where the experienced conflict is greater, conflict, however, has a more complex etiology. In particular, judgment takes longer when people are torn between utilitarian considerations of the greater good (saving many), and the suffering produced in others as an accidental side-effect. An increased RT is likely to have been caused by reasoning processes in order to explore a way to avoid the conflict, in either case.

As a matter of fact, the DPHMJ's central result concerning personal vs. impersonal dilemmas has already been challenged. McGuire et al. (2009) reanalyzed the data sets from Greene and colleagues and removed what they called “poorly endorsed items” (those dilemmas not designed carefully enough). After this procedure by McGuire et al., the key effect disappeared from the data (McGuire et al., 2009). Similarly, Ugazio et al. (2012), on their part, showed that both deontological *and* utilitarian responding could actually be triggered by *different emotions* with different motivational tendencies. In their study, disgust induction (an emotion that triggers *withdrawal* tendencies) resulted in more deontological moral judgments (i.e., refraining from harm), while anger induction (an emotion that triggers *approach* tendencies) resulted in more utilitarian moral judgments (i.e., committing harm). This finding doesn't fit the Dual Process account either, because the study shows how different *emotional* phenomena trigger both deontological *and* utilitarian moral judgment tendencies.

Therefore, we propose that a potentially more suitable account of moral judgment is one that gives a different role to emotions in moral judgment, specifically, to the importance of the arousal response which is triggered in the individual by the dilemmatic situation along the way suggested by the Affect Infusion Model (AIM) by Forgas (1995). This model posits that (i) arousal properties of the situation, (i) the motivational features of the emotions triggered by it, *and* (iii) the associated cognitive appraisal mechanisms, all play a crucial role in every judgment. This model also posits that *affect infusion* is a matter of degree: any judgment is also dependent on previous knowledge of the individual about the event or situation he or she is about to judge; this implies that it is dependent on deliberate reasoning *as well as* on the magnitude of the emotional arousal triggered by the event or situation.

See the Supplementary Material for a summary of limitations of the method.

## Conclusion

In this work, we have followed Hauser et al. view of moral dilemmas: “… the use of artificial moral dilemmas to explore our moral psychology is like the use of theoretical or statistical models with different parameters; parameters can be added or subtracted in order to determine which parameters contribute most significantly to the output” (Hauser et al., 2007). We have tried to control for the variables known to influence moral judgment, in order to find out which ones matter most, and how they interact.

One main result of this work is that, when dilemmas are validated, Greene's main effect of personal dilemmas partly disappears, for a more complex pattern, which casts doubt on the view that some moral judgments are the result of a deliberation, while others, the deontological ones, are reached emotionally. While higher arousal is related to deontological judgments, it is not true that deontological judgments are faster than utilitarian ones. Deontological judgments may take longer than utilitarian ones if, after taking time to weight the options, and to look for a way to minimize the transgression, one cannot find a way to choose not to violate one's principles.

Research with moral dilemmas holds fascinating possibilities to study the grounding psychological principles of human moral cognition. Contrary to the criticisms brought up against this methodology, and in line with an increasing number of other researchers, we believe that it is specifically the artificial nature of moral dilemmas that make this methodology so valuable. In any case, the scenarios described to us in moral dilemmas are not more artificial than the stories narrated in novels and movies where life-and death-decisions change the course of supposedly inevitable events. Besides, other abundant channels of information of that kind are the news on TV, radio, in the papers, and on the internet. They inform us of atrocities that happened around the corner of our house while we were sleeping, or of heartbreaking life-threatening situations that some individual in a war swept country has had to go through… Are moral dilemmas really all that unreal and artificial to us?

## Author note

All authors: Human Evolution and Cognition (IFISC-CSIC) and Department of Psychology, University of the Balearic Islands, Carretera de Valldemossa, km. 7.5, Building: Guillem Cifre de Colonya, 07122 Palma, Spain. Nadine K. Gut current affiliation: School of Psychology and Neuroscience, University of St Andrews, St Mary‘s Quad, South Street, St Andrews, KY16 9JP, UK; Strathclyde Institute of Pharmacy and Biomedical Sciences, University of Strathclyde, 161 Cathedral Street, Glasgow, G4 0RE, UK.

### Conflict of interest statement

The authors declare that the research was conducted in the absence of any commercial or financial relationships that could be construed as a potential conflict of interest.

## Footnotes

^1^Please note that study with a preliminary version of the revised set has recently been published (Christensen et al., 2012).

^2^For a detailed description of the dilemmas, see also Moore et al. (2008). For clarity it should be said that these 48 dilemmas are made up of 24 different short stories, which have a personal and an impersonal version each.

^3^We also considered removing the Bike Week Dilemma due to the act of acrobatics that it involves, but finally left it in. However, we encourage researchers to potentially reconsider this.

^4^Please note: in this arousal and valence norming procedure participants did not see the question. This was to avoid confounds between the arousal and valence judgments and a moral judgment.

^5^Supplementary Material accompanies this manuscript with all data points presented in this work.
