# Supplementary material for: Moral judgment reloaded: a moral dilemma validation study
Source: Front Psychol. 2014 Jul 1;5:607. doi: 10.3389/fpsyg.2014.00607 (PMC4077230; doi:10.3389/fpsyg.2014.00607)
Supplement: Supplementary file 1 [file DataSheet1.PDF]

Figure Legends

Figure 1. Example of the causal chain of the proposed moral transgression that leads to the salvation. For instance, in the *Instrumental version* of the **Burning Building** dilemma the proposed *action* is "to use the body of the victim". The *intention* is "use the body to break down burning debris". The victim *dies* directly by the fire and there is no independent *mechanism* in between. A larger number of people is saved due to the fact that the burning debris was eliminated *with the victim*. The harm to the victim was thus used *as a means* to save the others, or, said in different words, the body of the victim was literally used *instrumentally* to free the trapped group. Conversely, in the *Accidental version* of the **Iceberg** dilemma, the *action* of the protagonist is "to push the emergency access hatch". The *intention* behind that action is "to make the oxygen flow to the upper section of the boat". The victim dies *due to a knock on the head* by an independent *mechanism* which is the fal*ling down of the hatch*. Thus, the victim dies as a *side-effect* of the act of salvation that the protagonist carries out with the intention to get oxygen to the upper section of the boat.

Figure 2. The four factors in the dilemma set, adapted from Christensen & Gomila (2012). (1) *Personal Force*: the kind of imaginary involvement with the situation: Personal, as direct cause, or Impersonal, as an indirect agent in the process of harm. (2) *Benefit Receptor*: concerns whether the protagonist's life is at stake (Self-Beneficial action), or not (Other-Beneficial action). (3) *Evitability*: regards whether the victim is part of the group of people that would die if the moral transgression is not carried out (Inevitable death, the person would die anyway), or not (Avoidable death, the person would not die if no action is taken). (4) *Intention*ality: If the action is carried out

intentionally with the explicit aim to kill the person as a means to save others, this is Instrumental harm (it explicitly *needs* the death of that person to save the others). If the innocent person dies as a non-desired side-effect of the action by some independent mechanism and not directly by the action of the protagonist, the harm is Accidental.

Figure 3. Correlation between Arousal ratings and the RT. Color coding: *Personal Moral Dilemmas* (PMD; Blue/Red, circles); *Impersonal Moral Dilemmas* (IMD; Green/Yellow, squares). Arousal ratings are 1 = Not arousing, calm; 7 = Very arousing, on the x-axis. RT is in milliseconds (ms) on the y-axis. The numbers refer to the dilemma numbers in the dilemma set.

Figure 4. Curvilinar relationship between *Moral Judgment* and RT. Color coding: *Personal Moral Dilemmas* (Blue/Red, circles); *Impersonal Moral Dilemmas* (Green/Yellow, squares). Mean Likert scale responses: 1 = *No, I don't do it*, i.e. *deontological* moral judgment; 7 = *Yes, I do it*, i.e. *utilitarian* moral judgment. RT is in milliseconds (ms). PMD = Personal Moral Dilemmas; IMD = Impersonal Moral Dilemmas.

Figure 5. Relationship between level of arousal of a dilemma and the moral judgment made to that dilemma. Color coding: *Personal Moral Dilemmas* (Blue/Red, circles); *Impersonal Moral Dilemmas* (Green/Yellow, squares). Mean Likert scale responses: 1 = *No, I don't do it*, i.e. *deontological* moral judgment; 7 = *Yes, I do it*, i.e. *utilitarian* moral judgment. Mean Arousal scale responses: 1 = *Not arousing, calm*; 7 = *Very arousing*.
