# Supplementary material for: Moral judgment reloaded: a moral dilemma validation study
Source: Front Psychol. 2014 Jul 1;5:607. doi: 10.3389/fpsyg.2014.00607 (PMC4077230; doi:10.3389/fpsyg.2014.00607)
Supplement: Supplementary file 2 [file DataSheet2.PDF]

SUPPLEMENTARY MATERIAL (A) FOR

Christensen, J.F., Flexas, A., Gut, N.K., Calabrese, M., & Gomila, A. (*submitted*). Moral Judgment Reloaded: A Moral Dilemma validation study.

Additional tables and figures, and a summary of limitations

MOTIVATION TO USE MORAL DILEMMAS 2

TABLE S1 3

TABLE S2 3

TABLE S3 3

FIGURE S1 4

FIGURE S2. 4

FIGURE S3 5

FIGURE S4. 5

FIGURE S5. 6

FIGURE S6 6

FIGURE S7 7

FIGURE S8 8

FIGURE S9 9

LIMITATIONS 10

REFERENCES IN THE SUPPLEMENTARY MATERIAL 11

Motivation to use moral dilemmas

This methodology of moral dilemmas has been accused of (i) over-intellectualizing moral judgment; (ii) not addressing the everyday world of the selected subject population; and (iii) being simply based on unrealistic situations completely unlikely to occur to the "normal" (Western!) individual (see also Henrich, Heine, & Norenzayan (2010) on the pitfalls of mainstream subject populations).

While some of these qualms have some ground, they do not constitute insuperable roadblocks: (i) dilemmas can be made more realistic and truth-like; (ii) it is not uncommon to find oneself in a moral conflict, in fact this is rather a normal experience of human life; (iii) moral dilemmas are not at all alienated from experimental participants' "normal" life (many dilemmatic real-life scenarios receive extensive media coverage); and besides (iv) to test our moral intuitions in novel and/or unlikely circumstances is also a way to investigate their complex interactions (as the initial quote by Zimbardo points out). Thus, we advocate that moral dilemmas are a useful methodology, indeed with several advantages: first, they allow the inclusion of a large number of variables in the formulation, making possible a more holistic approach to what it is that triggers one moral judgment or another. Second, this approach allows the inclusion of all these variables under a high level of experimental control because the dilemmas are exactly the same for each individual participant, and not subjected to the variability that may occur when different individuals –and even actors– intervene in an experiment.

Table S1
Means and SD for Arousal and Valence ratings of the 16 categories (see supplementary material for means and SD for each dilemma)

	Arousal		***Valence***	
Variables	***Mean***	***SD***	***Mean***	***SD***
PMD_Self_Avo_Acc	6.2339	.80326	1.7097	.58358
PMD_Self_Avo_Instr	6.0121	1.07260	1.8871	.73785
PMD_Self_Ine_Acc	6.1532	1.14730	1.7258	.67555
PMD_Self_Ine_Instr	5.9032	1.12496	1.8172	.69523
PMD_Other_Avo_Acc	5.6452	1.22604	2.1129	.78625
PMD_Other_Avo_Instr	5.8831	1.07574	1.8992	.63288
PMD_Other_Ine_Acc	5.6613	1.27965	2.2258	.93070
PMD_Other_Ine_instr	5.8613	.99216	1.8613	.51034
IMD_Self_Avo_Acc	6.0000	1.13561	1.8280	.58126
IMD_Self_Avo_Instr	5.8548	1.11395	1.9731	.73749
IMD_Self_Ine_Acc	5.9624	.92349	2.0645	.66349
IMD_Self_Ine_Instr	6.0161	.97916	2.0645	.79178
IMD_Other_Avo_Acc	5.6774	1.02067	2.1720	.77704
IMD_Other_Avo_Instr	5.7742	1.03627	2.1022	.75769
IMD_Other_Ine_Acc	5.7581	1.10664	1.9409	.61114
IMD_Other_Ine_Instr	5.5806	1.04705	2.2849	.62680

Note: Abbreviations → IMD=Impersonal Moral Dilemmas, PMD=Personal Moral dilemmas; Self=Self-Beneficial, Other=Other-Beneficial, Avo=Avoidable, Ine=Inevitable; Acc=Accidental; Instr=Instrumental.

Table S2
Regression model predicting average Moral Judgment

Model:	*R^2*	*B*	*SE B*	*β*	*P*
	.511				
(Constant)		6.313	1.249		.000
Emotional Sensitivity (Bachorowski & Braaten, 1994)		-.015	.006	-.428	.018*
Emotional empathy (Mehrabian & Epstein, 1972)		.006	.004	.224	.188
Empathy (Davis, 1983)		-.017	.014	-.187	.246
Alexythmia (Taylor et al., 1985)		.012	.008	.245	.147
Big5 Neuroticism (McCrae & Costa, 1999)		-.016	.009	-.290	.079
Big5 Extraversion		.008	.010	.126	.434
Big5 Openness to Experience		-.010	.009	-.177	.244
Big5 Agreeableness		.027	.013	.353	.046*
Big5 Conscientiousness		.029	.008	.589	.001*
Thinking Style (Cacioppo, et al., 1984)		-.004	.005	-.130	.364

Dependent variable: mean Moral Judgment

Table S3
Model coefficients for the regression model of Arousal Ratings and Moral Judgment

Model	*B*	*SE B*	*R^2*	*β*	*P*
(Constant)	16.585	2.585			.000
Arousal Ratings	-2.090	.440	.339	-.583	.000

Dependent Variable: Moral Judgment

Figure S1. Overview of the 4 factors for the 2 x 2 x 2 x 2 design. The numbers refer to the levels as entered in the ANOVA.

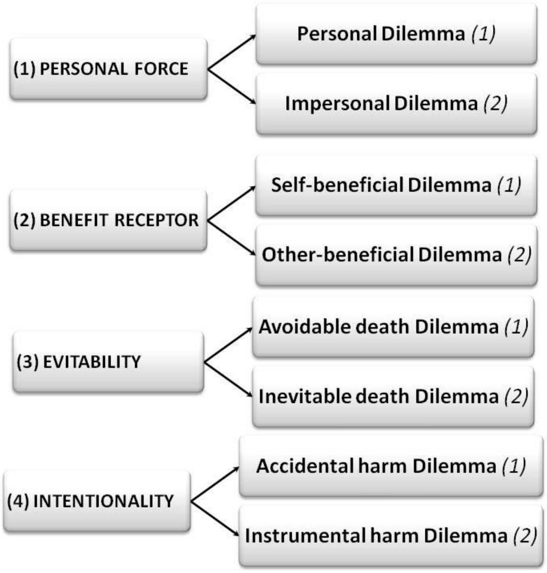

Figure S2. Illustration of the 16 dilemma categories, derived from the 2 x 2 x 2 x 2 design. The numbers refer to the variable denomination in the RM ANOVA.

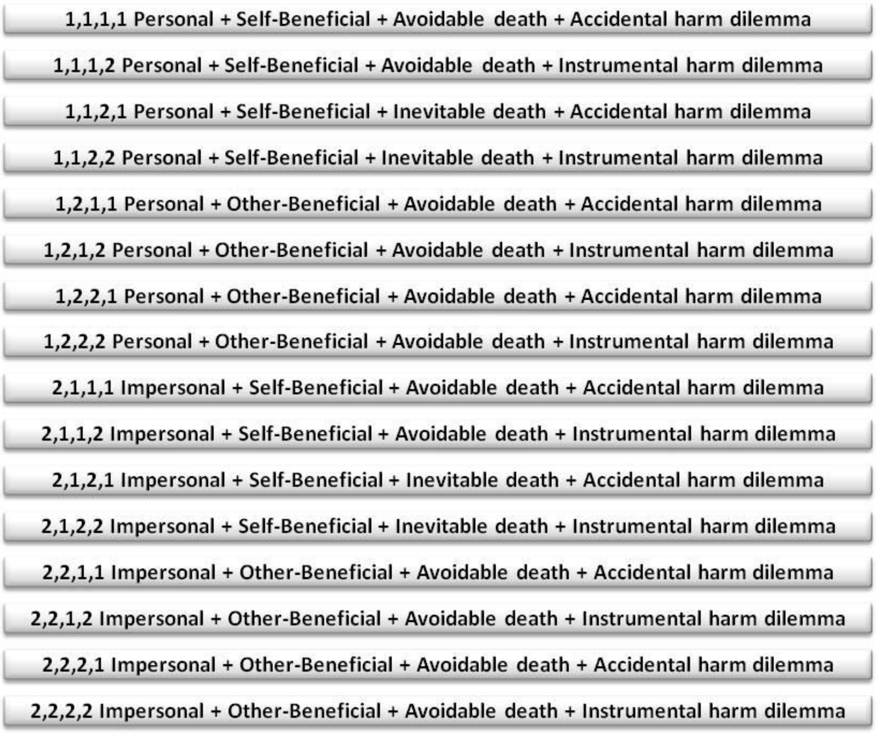

Figure S3. Arousal. Bars represent the means of the Likert scale responses (on the y-axis) of the two levels of each of the factors with a significant main effect: 1 = Not arousing, calm; 7 = Very arousing. Error-bars indicate SE. * = p < .05; ** = p < .001. (A) Main Effect of Personal Force. (B) Main Effect of Benefit Recipient.

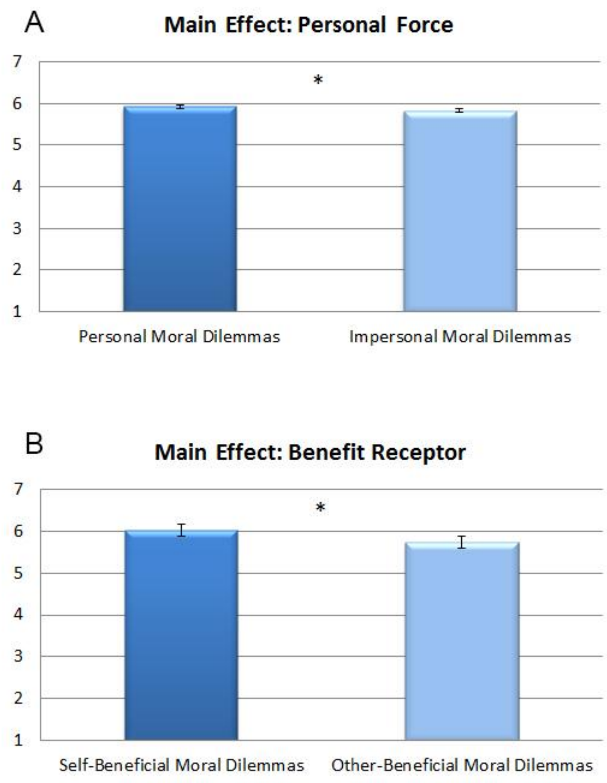

Figure S4. Arousal. Bar diagram of the Interaction Benefit Recipient*Intentionality. Mean Likert response on the y-axis: 1 = Not arousing, calm; 7 = Very arousing. Error-bars indicate SE. * = p < .05; ** = p < .001. The horizontal line designates the t-test between the two conditions on either side of it.

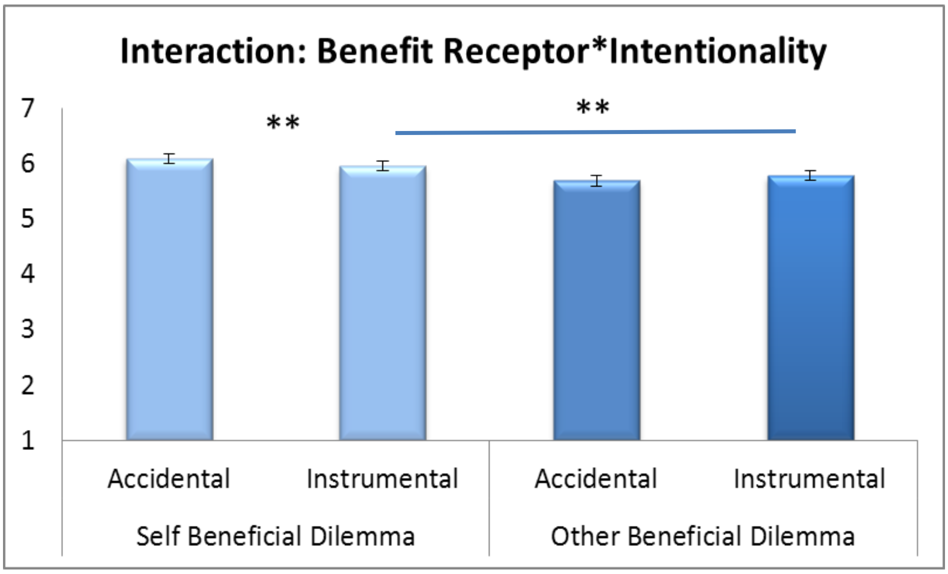

Figure S5. Valence. Bar diagram shows the interaction Personal Force*Intentionality. Mean Likert response on the y-axis: 1 = Very negative; 7 = Very positive. Error-bars indicate SE. * = p < .05; ** = p < .001.

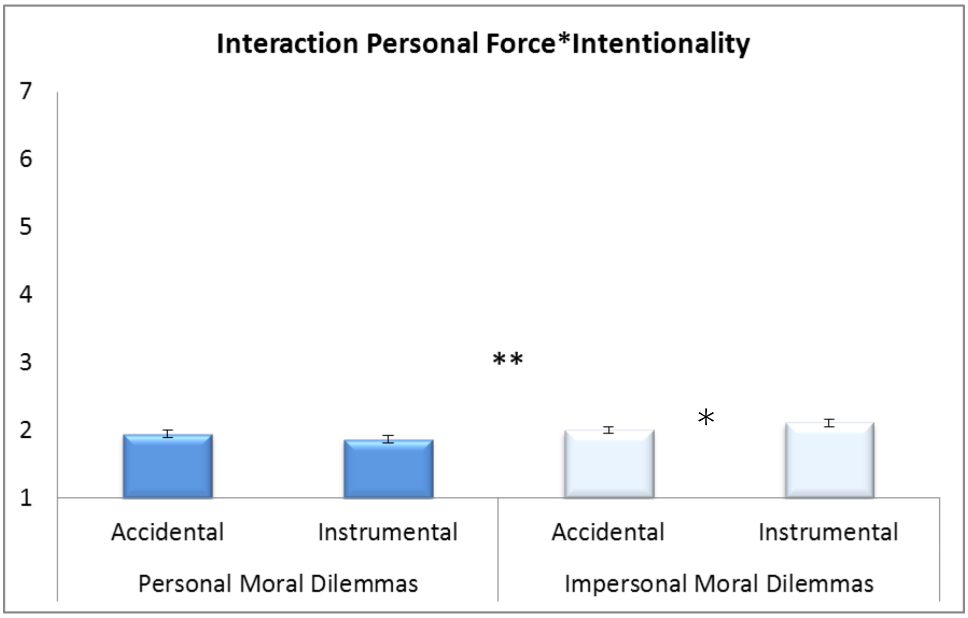

Figure S6. Valence. Bar diagram shows the interaction Benefit Recipient*Intentionality. Mean Likert responses on the y-axis: 1 = Very negative; 7 = Very positive. Error-bars indicate SE. * = p < .05; ** = p < .001. The horizontal lines designate the t-tests between the two conditions on either side of it.

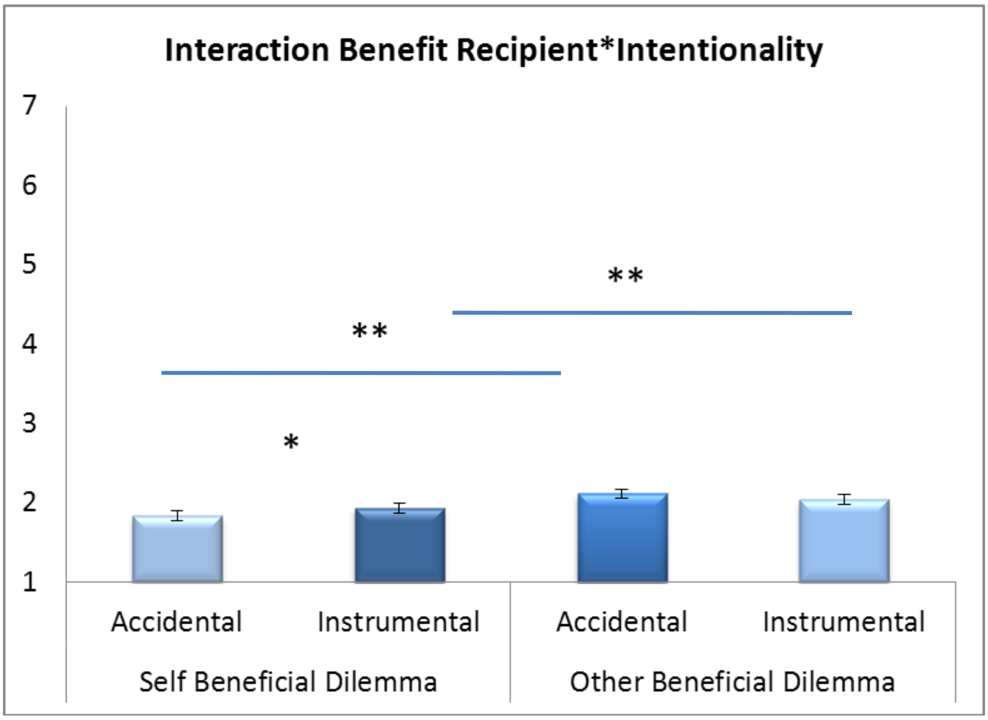

Figure S7. Mean Moral judgment in the 4 factors. Likert scale responses of the two levels of each of the factors with a significant main effect on the y-axis: 1 = No, I don't do it, i.e. deontological moral judgment; 7 = Yes, I do it, i.e. utilitarian moral judgment. Error-bars indicate SE. * = p < .05; ** = p < .001.

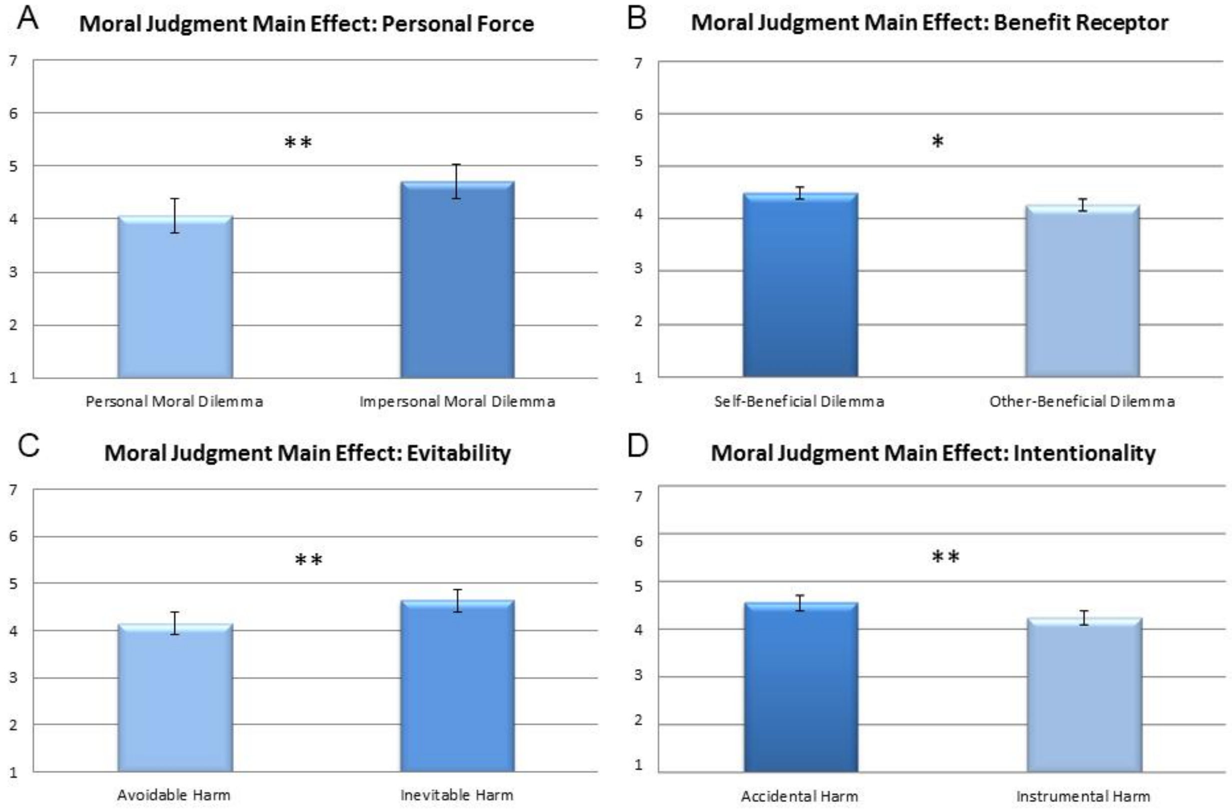

Figure S8. Interactions of the 4 factors in the variable moral judgment. Mean Likert scale responses on the y-axis: 1 = No, I don't do it, i.e. deontological moral judgment; 7 = Yes, I do it, i.e. utilitarian moral judgment. Error-bars indicate SE. * = p < .05; ** = p < .001. (A) Personal Force*Benefit Recipient. (B) Personal Force*Evitability. (C) Personal Force*Intentionality. (D) Benefit Recipient*Intentionality. (E) Evitability*Intentionality. The horizontal lines designate the t-tests between the two conditions on either side of it.

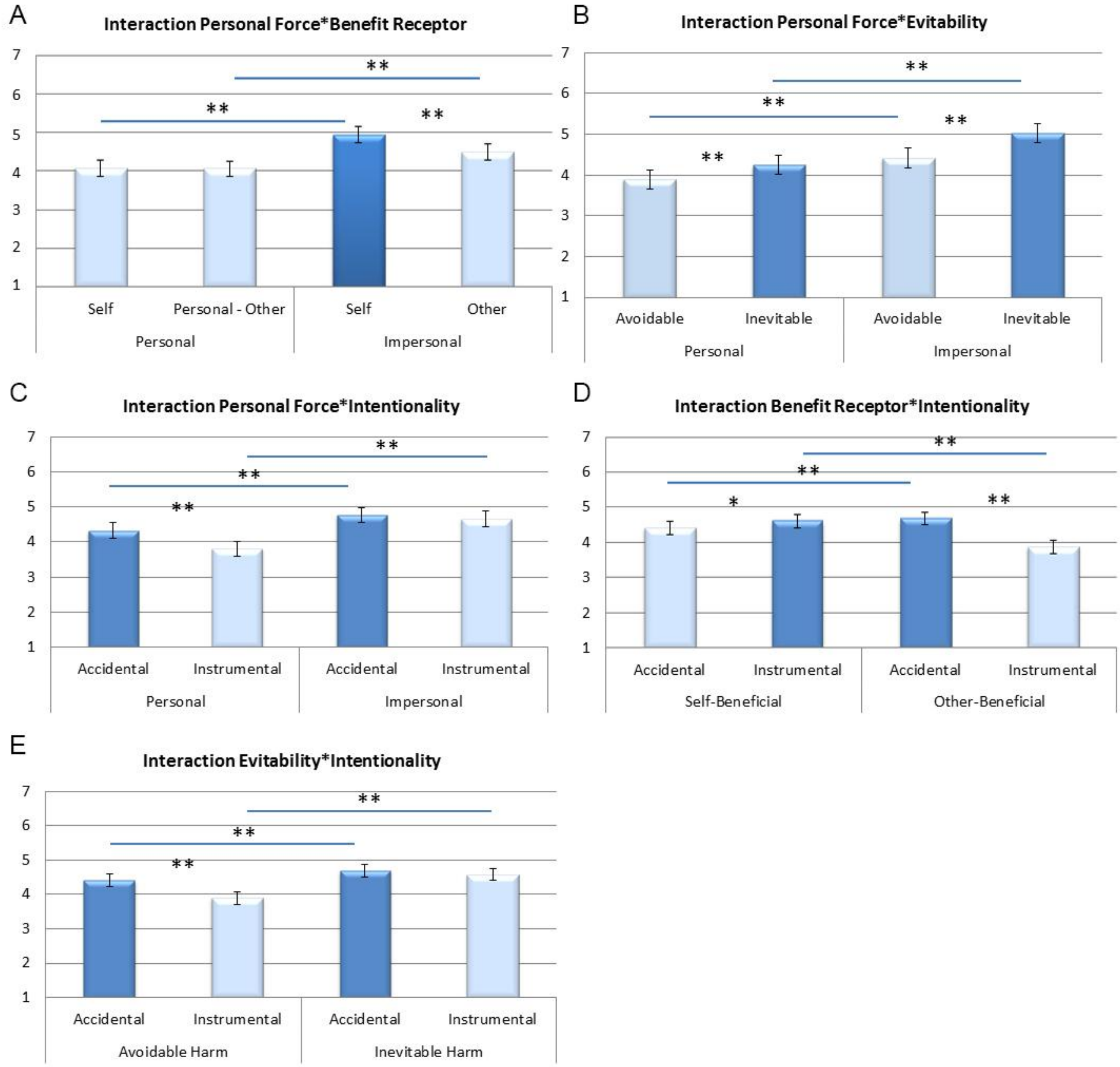

Figure S9. Interaction graph Type of Response*Intentionality. Error-bars indicate SE. ** = p = .003; † = p = .06, marginally significant. Mean Likert scale responses: 1 = No, I don't do it, i.e. deontological moral judgment; 7 = Yes, I do it, i.e. utilitarian moral judgment. RT is in milliseconds (ms) on the y-axis. The horizontal lines designate the t-tests between the two conditions on either side of it.

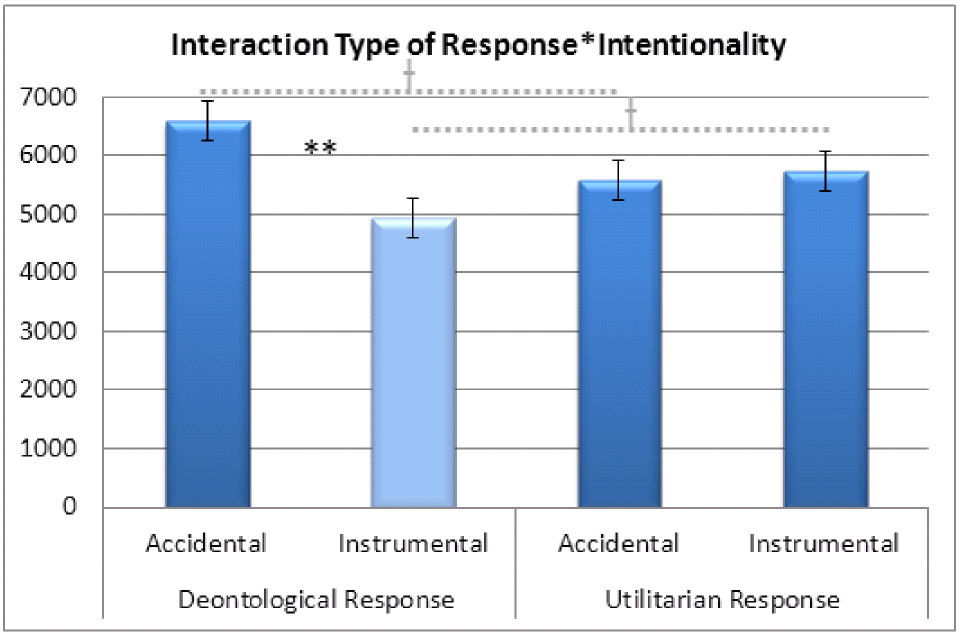

Limitations

First, the way arousal and valence were prompted in the experimental task may explain the high correlation between the two (see section 4). However, dilemmas could not have been presented in two repetitive blocks, having participants rate the dilemmas twice, once in arousal and once in valence due to the length of the task and the risk of participant fatigue. Besides, this problem was mediated by making the Likert scales opposite to minimize any facilitation effect that might occur (i.e. dilemmas were mostly “highly arousing”, meaning ratings towards 7 on that scale, while they were generally also “very negative”, meaning ratings towards 1 on that second scale).

Second, the methodology of how to present the question and the Likert scale on the screens may deserve a second look. In experiment 2 we certainly detected a methodological shortcoming. The Likert scale that participants used to indicate their moral judgment was displayed below the question while participants were reading the question. Hence, the danger exists that their RT effect was driven by the simple fact that the additional number of words resulted in a longer RT. By a separate regression analysis we made sure that RT was not generally influenced by the number of words of the question. For future studies, however, we recommend to follow the procedure proposed by Cushman (2008), who suggests to make an assessment of the mean reading time to the texts and to use this as an indicator for correct responding. In the case of the moral judgment task, maybe the question could be displayed during a fixed period of time before the participant at all could pass on to the Likert scale and make the rating.

Third, a skeptic may remark that the RT interaction we found between the *Response Type* (deontological *vs.* utilitarian) and *Intentionality* (Accidental *vs.* Instrumental Harm) could have been driven by the RT effect discussed above and in subsection 4 due to the two different question formats. However, if that were the case, the difference between *Accidental* and *Instrumental Harm* should also have been found for the utilitarian moral judgments, not only for the deontological ones. Taken together with the finding that the regression showed no overall effect of question-word-number on RT, this is as an indicator that the effect in the interaction is genuine. Nevertheless, we highlight this potential limitation so that future studies can take this matter into account.

Fourth, another criticism is that there was no baseline condition, e.g. *non-dilemmas* to contrast the judgments with. The reasons for this are three. First, there is still no convincing literature that defines what such thing as a *non-dilemma* is. Second, adding more short-stories to a dilemma set means to increment experiment-time, and with this participant fatigue. Third, by the same logics explained earlier, other types of dilemmas including, for instance, other types of harm could result in non-controllable carry over effects in the key dilemmas due to the mere fact that the contrast between the dilemmatic and non-dilemmatic situations is so big. Hence, the crucial conceptual differences between the dilemmas might be washed out when including “*non-dilemmas”*. Besides, there appears to be no need for another separate dilemma category as all hypothesized main effects and interactions were confirmed with the set as it is now, i.e. without any “*non-dilemmas*”.

Fifth, an important short-coming of this dilemma set is that there is not the same number of dilemmas in each category, and in one category (*Personal-Other-Beneficial-Inevitable-Accidental harm*) there is only one dilemma (see table 1). However, in view of the results from the present study, the grounds for future studies in the field is laid out and we encourage researchers to create further dilemmas following the rationale presented in this paper.

References in the supplementary material

Cushman, F. (2008). Crime and punishment: Distinguishing the roles of causal and intentional analyses in moral judgment. *Cognition, 108*(2), 353-380. doi:10.1016/j.cognition.2008.03.006

Henrich, Joseph, Heine, Steven J., & Norenzayan, Ara. (2010). The weirdest people in the world? *Behavioral and Brain Sciences, 33*(2-3), 61-135. doi: 10.1017/s0140525x0999152x

Zimbardo, Philip. (2007). *The Lucifer Effect*. New York: Random House Trade Paperbacks.
