# Supplementary material for: Moral judgment reloaded: a moral dilemma validation study
Source: Front Psychol. 2014 Jul 1;5:607. doi: 10.3389/fpsyg.2014.00607 (PMC4077230; doi:10.3389/fpsyg.2014.00607)
Supplement: Supplementary file 3 [file DataSheet3.PDF]

SUPPLEMENTARY -A- MATERIAL FOR

Christensen, J.F., Flexas, A., Gut, N.K., Calabrese, M., & Gomila, A. (*submitted*). Moral Judgment Reloaded: A Moral Dilemma validation study.

DILEMMAS IN 6 LANGUAGES

1. The moral dilemma set in six languages

Models on human moral cognition assume that some types of harm trigger universal moral intuitions and that these intuitions and their underlying psychological and neural mechanisms can be studied by means of moral cognition tasks, of which moral judgment is one (Greene, 2008; Greene, 2009; Greene, 2009; Greene, et al., 2009; Greene, et al., 2004; Haidt, 2001; Haidt, Graham, 2007; Moll, de Oliveira-Souza, & Eslinger, 2003; Moll, Oliveira-Souza, Zahn, Grafman, 2008). However, this assumption has not yet been systematically tested in an intercultural setting to be based on solid grounds.

So far, there is first evidence to suggest cultural variation in moral judgment for some variables, while other variables trigger particular kinds of moral judgment consistently throughout the few cultures studied (Abarbanell & Hauser, 2010; Cohen, Malka, Rozin, & Cherfas, 2006; Cohen & Rankin, 2004; Cohen, Siegel, & Rozin, 2003; Nucci & Turiel, 1993; O'Neill & Petrinovich, 1998). This makes the availability of a standard stimulus set to assess such cross-cultural variations rather pressing in the quest for an integrative moral theory.

1.1. Dilemma translation procedure

To facilitate cross-cultural studies on moral judgment with a standard set of moral dilemmas, we supply a translation of the present set in six languages: English, French, German, Spanish, Catalan and Danish. Obviously, we do not pretend that these six are sufficient to access all cultures of the world. However, with these six languages at hand, the dilemma set may be accessible to more countries where they can subsequently be translated into their own languages.

FRENCH is the spoken language not only in France, and in some regions of Switzerland, Belgium and Canada; it also makes a translation into many African languages more accessible, as French is a co-official language in many countries of Africa and Oceania. Similarly, **SPANISH** provides access to large parts of Central and South America and some parts of Africa (Ceuta, Mejilla, Equatorial Guinea), a part from being the official language in Spain (including Balearic and Canary Islands) with a number of total speakers of about 406 millions. **GERMAN** is spoken by about 80 million Germans, 7.6 million Swiss, 8.2 Austrians, and 75000 German speaking nationals of Belgium, but has also been extensively taught in Eastern European States and Russia (former members of the Soviet Union), and is still spoken by a large part of the population of Namibia (former German South-West Africa). **CATALAN** is a co-official language in Spain and has about 12 million speakers over Spain, France, Andorra and Italy (L'Alguer in Sardinia). **DANISH** is spoken by about 5 million Danes in Denmark and by about another 500.000 individuals across Greenland, the Faeroes and Iceland where Danish is a co-official language. It is also co-official language within the Danish minority of *Sydsleswig* (since 1920 called *Schleswig-Holstein*, North Germany). It is obvious that **ENGLISH** holds the lead in number of speakers all over the world; however, we believe

that the other five languages provide a means for further translation into languages and dialects maybe not so easily accessible through English (Lewis, 2009). This is recommendable as it appears that the English-speaking population of the world is the one most studied throughout the history of empirical research in psychology (Henrich, Heine, & Norenzayan, 2010). We encourage researchers to translate the dilemma set into other languages and to adapt it to regional dialects –and to make the resulting sets available on the homepage of this journal or otherwise to the scientific community.

1.2. Adaptation of the set to each of the 6 languages

The translations into all languages were carried out by native speakers of each of the languages, or of very proficient L2 speakers (i.e. non-native speakers with a high level of proficiency). All were members of our research group, which means that they had the required knowledge to translate according to the relevant conceptual design parameters. Furthermore, each set was then double checked by a second native speaker of each language who was naïve to the purposes of the study), who compared the translation with the English or Spanish texts as a pseudo back-translation process.

Further details about the different sets involve the following: first, all versions of dilemmas should always be adapted in terms of regional and dialectal differences as to how things are expressed. The English version is currently formulated in American format. Similarly, in Spanish particular action verbs have different meanings in Spain and in Central or South America. The Spanish set is in the Spanish dialect of Spain. Likewise, Swiss, Belgian, Canadian, Maghreb or French (France) needs regional adaptations. It is now formulated in French (France).

Second, researchers should be aware that in the Latin languages, the nouns have gender. This means that the participant perspective should be formulated according to whether the experimental participant is female or male. In this set, dilemmas are formulated for both female and male participants. We recommend that researchers prepare a female and a male version of the experimental paradigm.

Third, for German, French, Catalan and Spanish a decision must be made whether to use the informal (Du/Tu/Tu/Tu) or formal (Sie/Vous/Vostè/Usted) form in the address. In Danish and English this is irrelevant as the formal addresses are archaïc. Following our strictly subjective impression of cultural adequacy, for now, the French dilemmas are formulated with the formal address, while the German, Catalan and Spanish versions are formulated with the informal address. Researchers should be sensitive to the cultural relevance of formal and informal addresses and simply adapt the set according to the subject population they aim to study. See the table for the word counts in each language and the supplementary material for the dilemma sets in each language (supplementary material S1-S6) and for the word counts for each dilemma in each language (supplementary material S7).

Table
Mean Word count for all languages across dilemma categories

	Personal Moral Dilemmas							
	**Self-Beneficial**				**Other-Beneficial**			
	**Avoidable Harm**		**Inevitable Harm**		**Avoidable Harm**		**Inevitable Harm**	
Language	*Accidental*	*Instrumental*	*Accidental*	*Instrumental*	*Accidental*	*Instrumental*	*Accidental*	*Instrumental*
**Spanish**	132	134	156.5	121.75	132	114.75	119	128.2
**Catalan**	131.5	132.75	161	113.5	125	112.75	106	124
**French**	136	134.25	152	127.5	145	118.5	128	146.4
**English**	150	145.75	165.5	136	145.5	123.5	135	143.8
**German**	138.5	133.75	157.5	121	134	114	124	142
**Danish**	129.5	132	149.5	116.25	129.5	115.25	130	136.6
	**Impersonal Moral Dilemmas**							
	**Self-Beneficial**				**Other-Beneficial**			
	**Avoidable Harm**		**Inevitable Harm**		**Avoidable Harm**		**Inevitable Harm**	
Language	*Accidental*	*Instrumental*	*Accidental*	*Instrumental*	*Accidental*	*Instrumental*	*Accidental*	*Instrumental*
**Spanish**	133.33	153	141	129	125.67	122.67	133.5	142
**Catalan**	130.67	150	138.33	120.33	128	122	129.5	134.5
**French**	134.67	148	146	135.33	135.67	129.67	157	159.25
**English**	142.67	164	152.33	141.67	139.67	132.33	142.5	159.5
**German**	133	149.67	144.33	130.67	126.67	124	143	153.5
**Danish**	130	139.33	138.33	126.33	123.67	129.33	148.5	148.25
