# Supplementary material for: Moral judgment reloaded: a moral dilemma validation study
Source: Front Psychol. 2014 Jul 1;5:607. doi: 10.3389/fpsyg.2014.00607 (PMC4077230; doi:10.3389/fpsyg.2014.00607)
Supplement: Supplementary file 5 [file DataSheet5.DOC]

**Catalan**

**DILEMMES**

**1) Personal - Instrumental**

Tu i cinc persones més esteu atrapades dins d'un edifici en flames. Hi ha una única sortida d'emergència a través de la qual tots podríeu escapar, però està bloquejada per enderrocs ardents. Una altra persona ferida intenta arrossegar-se per una escletxa que queda a la base d'aquesta sortida. Tu i les altres cinc persones que tens darrere no teniu temps de fer el mateix.

Si uses la persona ferida per desbloquejar el pas a través dels enderrocs podreu escapar. Això segur que la matarà però vos salvarà a tu i a les cinc persones que duus a darrera.

Desbloqueges el pas usant la persona ferida perquè pogueu escapar tu i les altres cinc persones?

**2) Impersonal- Accidental**

Tu i cinc persones més estau atrapades dins d'un edifici en flames. Hi ha una única sortida d'emergència a través de la qual tots podríeu escapar, però està bloquejada per enderrocs ardents. Una altra persona ferida intenta arrossegar-se per una escletxa que queda a la base d'aquesta sortida. Tu i les altres cinc persones que tens darrere no teniu temps de fer el mateix.

Si actives el sistema d'emergència s'eliminarà l'oxigen del passadís, apagant el foc però deixant al ferit sense aire. Això segur que el matarà, però vos salvareu tu i les cinc persones que et vénen darrera.

Apagues el foc activant el sistema d'emergència, cosa que deixarà al ferit sense aire, per a què tu i les altres cinc persones pogueu escapar?

**3) Personal – accidental**

Soldats enemics han ocupat el teu poble i mataran a tots els civils majors de dos anys. Tu i deu veïns us refugieu en dues habitacions del soterrani d'una gran casa. Sentiu veus de soldats que han entrat a cercar coses de valor. El teu bebé comença a plorar fort. Els seus plors cridaran l'atenció dels soldats, que li perdonaran la vida, però vos mataran a tu i als altres deu refugiats.

Si li tapes la boca amb la mà esmorteiràs els seus plors, però el bebé se quedarà sense aire. Això el matarà però vos salvarà a tu i als deu veïns.

Esmorteeixes els seus plors mantenint la mà a la boca del teu bebé per a que els soldats no us trobin a tu i als deu veïns?

**4) Impersonal- accidental**

Soldats enemics han ocupat el teu poble i mataran a tots els civils majors de dos anys. Tu i deu veïns us refugiau en dues habitacions del soterrani d'una gran casa. Sentiu veus de soldats que han entrat a cercar coses de valor. El teu bebé comença a plorar fort. Els seus plors cridaran l'atenció dels soldats, que li perdonaran la vida, però us mataran a tu i als altres deu refugiats.

Si actives una estruendosa caldera que hi ha es produirà una incòmoda calor per als adults, que serà mortal per al teu bebé, però esmorteïrà els seus plors, cosa que vos salvarà a tu i als deu veïns.

Esmorteeixes els plors del teu bebé, activant la caldera que l'asfixiarà, per a què no vos trobin a tu i als deu veïns?

**5) Personal – accidental**

Ets part de la tripulació d'un submarí que navega sota un gran iceberg. Una explosió ha fet malbé la nau, ferint a diversos companys i col·lapsant l'únic accés entre les parts superior i inferior. Tu i altres deu supervivents estau a la secció superior, que no té suficient oxigen per mantenir-vos amb vida fins a sortir a la superfície. A la secció inferior, on hi ha suficient oxigen, jeu inconscient un únic company.

Si empenys la comporta d'emergència entre ambdues seccions, obriràs el pas de l'aire. Ara bé, la comporta caurà sobre el company d'abaix, matant-lo, però això vos salvarà a tu i als altres deu supervivents.

Obres el pas de l'aire empenyent la comporta que caurà sobre el company, per a què tu i els altres deu supervivents tengueu suficient oxigen?

**6) Impersonal- accidental**

Ets part de la tripulació d'un submarí que navega sota un gran iceberg. Una explosió ha danyat la nau, ferint a diversos companys i col·lapsant l'únic accés entre les parts superior i inferior. Tu i altres deu supervivents estau en la secció superior, que no té suficient oxigen per mantenir-vos amb vida fins a sortir a la superfície. A la secció inferior, on hi ha suficient oxigen, jeu inconscient un únic company.

Si prems un interruptor d'emergència s'obrirà una comporta entre ambdues seccions, deixant passar l'aire. No obstant això, la comporta caurà sobre el company, matant-lo, però això vos salvarà a tu i als altres deu supervivents.

Obres el pas de l'aire prement l'interruptor, cosa que farà caure la comporta sobre el company, per a que tu i els deu supervivents tengueu suficient oxigen?

**7) Personal** – **instrumental**

Tu i deu submarinistes formau part d'un equip de les Nacions Unides que desactiva mines submarines de la IIª Guerra Mundial. Un membre del teu equip s'ha ferit i la sang ha atret a diversos taurons. Tens un fusell submarí, però només un arpó, i hi ha molts taurons. El submarinista que sagna està nedant cap a l'última gàbia protectora i hi arribarà abans que tu i els altres. Els taurons, seguint la sang, s'apropen massa per a què tu i els altres submarinistes pogueu escapar.

Si dispares al submarinista ferit, això el matarà i els taurons s'aturaran a menjar-se'l, però tu i els altres submarinistes podreu salvar-vos.

Fas que els taurons es mengin al submarinista ferit disparant-li, per a que tu i els altres nou submarinistes pogueu arribar a la gàbia protectora?

**8) Impersonal-Instrumental**

Tu i deu submarinistes formau part d'un equip de les Nacions Unides que desactiva mines submarines de la IIª Guerra Mundial. Un membre del teu equip s'ha ferit i la sang ha atret a diversos taurons. Tens un fusell submarí, però només un arpó, i hi ha molts taurons. El submarinista que sagna està nedant cap a l'última gàbia protectora i hi arribarà abans que tu i els altres. Els taurons, seguint la sang, s'apropen massa per a que tu i els altres submarinistes pogueu escapar.

Si detones per control remot una de les mines, això el matarà i els taurons s'aturaran a menjar-se'l, però tu i els altres submarinistes vos podreu salvar.

Fas que els taurons es mengin el submarinista ferit detonant per control remot la mina que té devora, per a què tu i els altres nou submarinistes pogueu arribar a la gàbia protectora?

**9) Personal – instrumental**

Treballes en un orfenat d'un país en guerra qu’acull deu orfes. Soldats rebels s'han fet amb el govern i estan matant a tots els que no recolzen el seu règim i a més duen a terme una campanya genocida contra un determinat grup ètnic. Tu i els deu orfes no recolzau la rebel·lió i a més un dels infants és del grup ètnic perseguit. No hi ha forma de que tu i els orfes pogueu escapar i segur que els soldats vos mataran a tots.

Si asfixies amb un coixí l'infant del grup ètnic perseguit mentres dorm, els soldats creuran que recolzes el seu règim. Això el matarà, però vos salvarà a tu i als altres nou infants.

Fas que els soldats creguin que recolzes el seu règim asfixiant l'infant, per a salvar la vida dels altres nou infants i la teva?

**10) Impersonal – Instrumental**

Treballes en un orfenat d'un país en guerra qu’acull deu orfes. Soldats rebels s'han fet amb el govern i estan matant a tots els que no recolzen el seu règim i a més duen a terme una campanya genocida contra un determinat grup ètnic. Tu i els deu orfes no recolzau la rebel·lió i a més un dels infants pertany al grup ètnic perseguit. No hi ha forma de que tu i els orfes pogueu escapar i segur que els soldats vos mataran a tots.

Si ordenes a l'infermera que li doni al nen del grup ètnic perseguit una sobredosi letal de píndoles per dormir, els soldats creuran que recolzes el seu règim. Això el matarà, però vos salvarà a tu i als altres nou nens.

Fas que els soldats creguin que recolzes el seu règim ordenant a l'infermera que doni una sobredosi al nen per a que vos perdonin la vida a tu i als altres nou nens?

**11) Personal – instrumental**

Ets treballador/a de la salut voluntari/ària en un poble rural africà. Un home d'un poble veí ha contret el virus de l'Ébola, que és extremadament contagiós, incurable i gairebé sempre mortal. Miraculosament, ha sobreviscut un mes, per la qual cosa deu ser immune als efectes mortals del virus. No obstant això, ell creu erròniament que el teu centre de salut pot curar-lo per complet. El veus aproximant-se i saps que si entra al poble contagiarà als 119 habitants innocents que, a diferència d'ell, moriran.

Si li dispares amb la pistola del centre evitaràs que vos contagïi el virus a tu i a la resta del poble. Això el matarà, però et salvarà a tu i als 119 habitants del poble.

Impideixes que l’home entri al poble, disparant-li per a què no vos contagïi a tu i als 119 habitants del poble?

**12) Impersonal-Instrumental**

Ets treballador/a de la salut voluntari/ària en un poble rural africà. Un home d'un poble veí ha contret el virus de l'Ébola, que és extremadament contagiós, incurable i gairebé sempre mortal. Miraculosament, ha sobreviscut un mes, per la qual cosa deu ser immune als efectes mortals del virus. No obstant això, ell creu erròniament que el teu centre de salut pot curar-lo per complet. El veus aproximant-se i saps que si entra al poble contagiarà als 119 habitants innocents que, a diferència d'ell, moriran.

Si avises al consell del poble li dispararan per prevenir que vos contagïi el virus a tu i la resta del poble. Això el matarà, però et salvarà a tu i als 119 habitants del poble.

Impideixes que l’home entri al poble avisant al consell que li dispari, per a que no vos contagïi a tu i als 119 habitants?

**13) Personal – instrumental**

Ets l'únic/a metge/ssa d'un helicòpter de rescat en una inundació devastadora. Has rescatat nou persones ferides en estat crític i les mantens vives. De sobte, el pilot et notifica que el motor està fallant i que cal reduir càrrega o l'helicòpter s'estavellarà i tots morireu. Tu no pots abandonar l’helicòpter perquè els ferits han de menester la teva cura, ni pots llençar el feixuc equipament mèdic perquè està engramponat.

Si llences un dels ferits reduiràs càrrega suficient per mantenir l’helicòpter en l’aire. Això el matarà però vos salvarà a tu, al pilot i als altres vuit ferits.

Redueixes càrrega llençant un dels ferits, per mantenir l'helicòpter en l'aire i salvar les altres deu persones a bord?

**14) Impersonal-Instrumental**

Ets l'únic/a metge/ssa d'un helicòpter de rescat en una inundació devastadora. Has rescatat nou persones ferides en estat crític i les mantens vives. De sobte, el pilot et notifica que el motor està fallant i que cal reduir càrrega o l'helicòpter s'estavellarà i tots morireu. Tu no pots abandonar l’helicòpter perquè els ferits han de menester la teva cura, ni llençar el feixuc equipament mèdic perquè està engramponat.

Si talles el cable pel que puja la darrera persona rescatada reduiràs pes suficient per mantener l’helicòpter en l’aire. Això el matarà, però vos salvarà a tu, al pilot i als altres vuit ferits a bord.

Redueixes pes tallant el cable pel qual ascendeix el darrer dels ferits per mantener l'helicòpter en l’aire i salvar les altres deu persones a bord?

**15) Personal – accidental**

Ets enginyer/a en l'Estació Espacial Internacional que orbita la Terra. De sobte es declara un incendi a la bodega de càrrega. El sistema anti-incendis automàtic funciona obrint la comporta exterior de la bodega per a que l'oxigen surti i així apagar el foc, però només quan la comporta interior està bloquejada. El problema és que hi ha un mecànic a l'interior de la bodega. Veus que no té temps de llevar-se el pesat vestit d'astronauta, i que intenta passar per la comporta interior tal com està, però que hi quedarà atascat, causant que el foc s'extengui i us mati a tots.

Si empenys el mecànic cap a dins la bodega podràs bloquejar la comporta interior, i així s'activarà el sistema anti-incendis. Això enviarà el mecànic a l'espai, matant-lo, però vos salvarà a tu i als nou astronautes restants.

Empenys el mecànic per a poder bloquejar la comporta interior i així activar el sistema anti-incendis, cosa que enviarà al mecànic a l'espai, per a que el foc no vos arribi a tu i els altres nou astronautes?

**16) Impersonal – accidental**

Ets enginyer/a en l'Estació Espacial Internacional que orbita la Terra. De sobte es declara un incendi a la bodega de càrrega. El sistema anti-incendis automàtic funciona obrint la comporta exterior de la bodega per a que l'oxigen surti i així apagar el foc, però només quan la comporta interior està bloquejada. El problema és que hi ha un mecànic a l'interior de la bodega. Veus que no té temps de llevar-se el pesat vestit d'astronauta, i que intenta passar per la comporta interior tal com està, però que hi quedarà atascat, causant que el foc s'extengui i us mati a tots.

Si prems l'interruptor que bloqueja la comporta interior abans que el mecànic hi arribi, s'activarà el sistema anti-incendis. Això enviarà al mecànic a l'espai, matant-lo, però vos salvarà a tu i als nou astronautes restants.

Apagues el foc prement l'interruptor que bloqueja la comporta, cosa que enviarà al mecànic a l'espai, per a què el foc no vos arribi a tu i als altres deu astronautes?

**17) Personal – instrumental**

Ets inspector/a en una planta d'energia nuclear. El director i tu l'esteu visitant quan un dels conductes d'abastiment es sobreescalfa. El sistema refrigerador d'emergència falla i està a punt de començar una reacció en cadena que resultarà en fusió nuclear. La radiació alliberada serà letal per a la ciutat propera, matant a milers de persones.

Si empenys el director al lloc on conflueixen els conductes d'abastiment, el seu cos absorbirà prou calor per detenir la reacció en cadena. Això el matarà, però vos salvarà a tu i als milers d'habitants de la ciutat.

Refredes els conductes d'abastiment empenyent el director al lloc on conflueixen per a evitar un accident nuclear que vos mataria a tu i a milers de persones?

**18) Impersonal – accidental**

Ets inspector/a en una planta d'energia nuclear. El director i tu l'esteu visitant quan un dels conductes d'abastiment es sobreescalfa. El sistema refrigerador d'emergència falla i està a punt de començar una reacció en cadena que resultarà en fusió nuclear. La radiació alliberada serà letal per a la ciutat propera, matant a milers de persones.

Si insertes manualment nitrogen líquid en la càmara d'abastiment s'eliminarà suficient calor per detenir la reacció en cadena. Això matarà a un treballador atrapat a la càmara, però vos salvarà a tu i als milers d'habitants de la ciutat.

Elimines calor insertant nitrogen líquid a la càmara d'abastiment on hi ha un treballador, per a evitar un accident nuclear que vos mataria a tu i a milers de persones?

**19) Personal – instrumental**

Ets l'expert/a en explosius d'una companyia encarregada de demolir un gratacel. Estàs examinant l'última càrrega quan veus a un noi que és a punt de detonar accidentalment una de les càrregues. L'explosió resultarà en l'esfondrament sense control de l'edifici a sobre teu, del noi i de la multitud d'espectadors. El noi està diversos pisos per sota de tu i no pot sentir-te.

Si deixes caure un pesat bloc de formigó sobre el seu cap, això el matarà, però vos salvarà a tu i a la multitud perque evitarà l'explosió.

Atures al noi deixant caure el bloc sobre el seu cap per a què l'edifici no s'esfondri sobre tu i la multitud d'espectadores?

**20) Impersonal-Instrumental**

Ets l'expert/a en explosius d'una companyia encarregada de demolir un gratacel. Estàs examinant l'última càrrega quan veus a un noi que és a punt de detonar accidentalment una de les càrregues. L'explosió resultarà en l'esfondrament sense control de l'edifici a sobre teu, del noi i de la multitud d'espectadors. El noi està diversos pisos per sota de tu i no pot sentir-te.

Si actives el diferencial tornant l'electricitat a l'edifici, el noi s'electrocutarà perquè està tocant un circuït obert. Això el matarà, però vos salvarà a tu i a la multitud perque evitarà l'explosió.

Atures al noi, fent que s'electroci mitjançat l'activació del diferencial, per a què l'edifici no s'esfondri sobre tu i la multitud d'espectadors?

21) **Personal – instrumental**

Ets treballador/a de la construcció. Tu i el teu equip treballeu sobre una bastida en un gratacel. De sobte la bastida s'esfondra parcialment. Tu i cinc companys estau suspesos d'un travesser penjant, però peseu massa perquè resisteixi. Un altre company patina del travesser i s'aferra del teu braç. L'estructura és a punt de cedir.

Si copeges repetidament aquest company perquè et deixi anar i caigui, això restarà suficient pes per mantenir la bastida. La caiguda el matarà, però vos salvarà a tu i als altres cinc companys.

Alliberes pes copejant aquest company, que caurà, perquè tu i els altres cinc companys pogueu quedar penjant del travesser?

**22) Impersonal-Instrumental**

Ets treballador/a de la construcció. Tu i el teu equip treballeu sobre una bastida en un gratacel. De sobte la bastida s'esfondra parcialment. Tu i cinc companys estau suspesos d'un travesser penjant, però peseu massa perquè resisteixi. Un altre company patina del travesser i s'aferra d'una altra part de la bastida. L'estructura és a punt de cedir.

Si lleves el passador que subjecta la part on és aquest company ell caurà. Això restarà suficient pes per mantenir la bastida. La caiguda matarà al company, però vos salvarà a tu i als altres cinc companys.

Alliberes el pes del company llevant el passador per a que tu i els altres cinc companys pogueu quedar penjant del travesser?

**23) Personal – accidental**

Ets conductor/a d'un autocar de guarderia d'excursió al zoo. De camí, un defecte en les rodes provoca que us precipiteu des d'un pont a un riu brau. Tu i tres nens esteu encara en el vehicle que s'enfonsa. La resta han sigut arrossegats pel riu. Agafes els dos nens que tens devora i nedes cap a la sortida, però el tercer nen s'aferra a la teva cama. No ets prou fort/a per lluitar contra el corrent i nedar amb els tres nens.

Si et desfas del nen que s'ha aferrat de la teva cama podràs lluitar contra el corrent, però el nen quedarà enrera, ofegant-se. Això el matarà però vos salvarà a tu i als altres dos nens.

Lluites contra el corrent desfent-te del nen, cosa que farà que el nen s'ofegui, per a què tu i els altres dos nens pogueu arribar a la superfície?

**24) Impersonal-accidental**

Ets conductor/a d'un autocar de guarderia d'excursió al zoo. De camí, un defecte en les rodes provoca que us precipiteu des d'un pont a un riu brau. Tu i tres nens esteu encara en el vehicle que s'enfonsa. La resta han sigut arrossegats pel riu. Agafes els dos nens que tens devora i nedes cap a la sortida, però està bloquejada amb alguns seients destrossats.

Si lleves els seients alliberaràs la sortida però obstaculitzaràs el passadís i el tercer nen quedarà atrapat, ofegant-se. Això el matarà però vos salvarà a tu i als altres dos nens.

Alliberes la sortida posant els seients al passadís, cosa que atraparà al tercer nen, per a què tu i els altres dos nens pogueu arribar a la superfície?

**(25) Personal – instrumental**

Ets doctor/a. Tens cinc pacients que moriran si no reben el trasplantament inmediat d'un òrgan, distint en cada cas. Una altra persona arriba a l'hospital després d'un seriós accident de cotxe. El seu estat és crític, però és probable que puguis salvar-la mitjançant una llarga i complicada operació.

Si talles a propòsit la seva artèria caròtida durant la cirurgia causaràs la seva mort sense que ningú ho sàpiga i disposaràs dels òrgans per als altres cinc pacients. Això matarà a la víctima de l'accident però salvarà als altres cinc pacients.

Obtens els òrgans tallant l'artèria caròtida de la víctima de l'accident per a poder realitzar els trasplantaments als altres cinc pacients?

**26) Impersonal-Instrumental**

Ets doctor/a. Tens cinc pacients que moriran si no reben el trasplantament inmediat d'algun òrgan, distint en cada cas. Una altra persona arriba a l'hospital després d'un seriós accident de cotxe. El seu estat és crític, però és probable que puguis salvar-la. Durant la llarga i complicada operació veus que una infermera va a injectar-li una medicació inadequada, letal en aquest cas.

Si deixes que l'infermera injecti la medicació errònia, la víctima de l'accident morirà i disposaràs dels òrgans necessaris per als altres cinc pacients. Això matarà a la víctima però salvarà als cinc pacients.

Obtens els òrgans deixant que l'infermera injecti la medicació errònia a la víctima de l'accident per a poder realitzar els trasplantaments als altres cinc pacients?

**27) Personal – accidental**

Ets un estibador que subjecta els cables de la grua per descarregar els vaixells mercants. Tu, amb altres companys, subjecteu aquests cables a un contenidor i munteu sobre ell per assegurar que es descarrega adequadament. De sobte veus la llum vermella que indica que el cable és a punt de fallar. T'adones que la culpa la tenen dos companys que s'estan barallant. Fan balancejar el contenidor perillosament sobre cinc companys que són al moll.

Si empenys els qui es barallen per separar-los evitaràs que el contenidor caigui, però un d'ells no porta el seu arnès de seguretat i caurà. Això el matarà però salvarà als cinc companys d'abaix.

Atures el balanceig del contenidor empenyent els qui es barallen, cosa que farà que el company sense arnès caigui, per a evitar que el contenidor es desplomi sobre els cinc companys d'abaix?

**28) Impersonal – accidental**

Ets un estibador que subjecta els cables de la grua per descarregar els vaixells mercants. Tu, amb altres companys, subjecteu aquests cables a un contenidor i munteu sobre ell per assegurar que es descarrega adequadament. De sobte veus la llum vermella que indica que el cable és a punt de fallar. Si ho fa sobre la coberta, el contenidor caurà sobre cinc tripulants.

Si prems el botó de seguretat, el contenidor caurà novament a la bodega. Vosaltres quedareu suspesos en l'aire pels arnesos de seguretat, però hi ha un tripulant encara a la bodega. El contenidor caurà sobre ell i el matarà, però se salvaran els cinc tripulants de la coberta.

Prems el botó de seguretat per amollar el contenedor, cosa que farà que caigui sobre el tripulant de la bodega, per a evitar que caigui sobre els cinc tripulants de la coberta?

**29) Personal – accidental**

S'ha escampat una epidèmia per tot el planeta, matant milers de persones. Ets investigador/a mèdic/a i has desenvolupat dues substàncies en el laboratori. Per error ambdues estan etiquetades com a vacuna. Una d'elles certament és la vacuna, que produïda en massa salvarà milers de vides, l'altra és una substància letal.

Si injectes aquestes dues substàncies, sense saber quina és quina, als teus dos ajudants de laboratori, podràs identificar la vacuna. Un dels ajudants morirà, però podràs salvar milers de vides amb la vacuna.

Identifiques la vacuna injectant les dues substànces als teus ajudants, cosa que farà que un d'ells sigui enverinat, per a què es puguin vacunar milers de persones?

**30) Impersonal-accidental**

S'ha escampat una epidèmia per tot el planeta, matant milers de persones. Ets investigador/a mèdic/a i has desenvolupat dues substàncies en el laboratori. Per error ambdues estan etiquetades com a vacuna. Una d'elles certament és la vacuna, que produïda en massa salvarà milers de vides, l'altra és una substància letal.

Si permets que un infermer injecti aquestes dues substàncies, sense saber quina és quina, a dos pacients, podràs identificar la vacuna. Un dels pacients morirà, però podràs salvar milers de vides amb la vacuna.

Identifiques la vacuna permetent que un infermer injecti les dues substànces a dos pacients, cosa que farà que un d'ells sigui enverinat, per a que es puguin vacunar milers de persones?

**31) Personal – instrumental**

Un tramvia fora de control es dirigeix a tota velocitat per les vies cap a cinc treballadors, que moriran si el tramvia segueix el seu curs. Tu estàs al costat de les vies però massa lluny per avisar-los. Al teu costat hi ha un desconegut molt gran.

Si empenys el desconegut a les vies, el tramvia descarrilarà en atropellar-lo i no seguirà el seu curs cap als treballadors. Això matarà el desconegut, però salvaràs als cinc treballadors.

Fas descarrilar el tramvia empenyent el desconegut a les vies per a que el tramvia no arribi als cinc treballadors?

**32) Impersonal – accidental**

Un tramvia fora de control es dirigeix a tota velocitat per les vies cap a cinc treballadors, que moriran si el tramvia segueix el seu curs. Tu estàs al costat de les vies però massa lluny per avisar-los. Al teu costat hi ha una palanca de control que pot reconduir el tramvia a una altra via on hi ha un únic treballador.

Si acciones la palanca, redirigiràs el tramvia a la via on hi ha un treballador i no seguirà el seu curs cap als cinc treballadors. Això matarà a aquest treballador, però salvaràs als altres cinc.

Redirigeixes les vies accionant la palanca, cosa que farà que un treballador sigui atropellat, per a que el tramvia no segueixi el seu curs cap als altres cinc treballadors?

**33) Personal – instrumental**

Tu i un company d'investigació heu descobert una nova font d'energia que és barata, neta i segura. Podria acabar amb la contaminació i la pobresa al món. Ara bé, el teu col·lega vol vendre el descobriment, i saps que avui planeja contactar amb potencials compradors. També saps amb seguretat que aquests intentaran emprar l'energia com a un arma horrible, però el teu company no te creu.

Si enverines el teu company amb un verí que normalment es troba al vostre laboratori, evitaràs que el descobriment caigui en males mans. Aquesta substància matarà el teu company però salvaràs milers de persones.

Impedeixes la venda del descobriment enverinant al teu company per a que la font d'energia no s'utilitzi com a arma contra milers de persones?

**34) Impersonal-Instrumental**

Tu i un company d'investigació heu descobert una nova font d'energia que és barata, neta i segura. Podria acabar amb la contaminació i la pobresa al món. Ara bé, el teu col·lega vol vendre el descobriment, i saps que avui planeja contactar amb potencials compradors per correu electrònic. També saps amb seguretat que aquests intentaran emprar l'energia com un arma horrible, però el teu company no te creu.

Si alliberes un gas inflamable en el laboratori, hi haurà una explosió en el moment en què el teu company encengui el seu ordinador. Això el matarà però salvaràs milers de persones.

Fas explotar l'ordinador del teu company alliberant el gas inflamable per evitar que el descubriment no s'utilitzi com a arma contra milers de persones?

**35) Personal – instrumental**

Ets un/a expert/a motorista que participa en un esdeveniment de motos. Mentres vas a davant d'un grup de 10 motoristes veus com un altre motorista que va més envant està perdent el control de la seva moto. Mentre acceleres per posar-te al seu costat t'adones que sofrirà una caiguda en qualsevol moment, provocant un accident en cadena en el que poden morir els deu motoristes que vénen darrera teu.

Si empenys aquest motorista fora de la carretera s'estavellarà contra els arbres, però evitaràs l'accident en cadena. A la vostra velocitat, això el matarà, però salvaràs els deu motoristes.

Evites l'accident en cadena empenyent el motorista fora de la carretera i salvar així la vida dels altres deu motoristes?

**36) Impersonal-instrumental**

Ets un/a expert/a motorista que participa en un esdeveniment de motos. Mentres vas a davant d'un grup de deu motoristes veus com un altre motorista que va més envant està perdent el control de la seva moto i cau. El pots esquivar fàcilment, però els deu motoristes darrera teu hi xocaran i moriran en l'accident en cadena que es produïrà.

Si deixes que la teva moto atropelli el motorista caigut, mentres saltes hàbilment cap a la cuneta, alertaràs a temps a la resta. Això matarà el caigut, però salvaràs els altres deu motoristes.

Alertes de l'accident deixant que la teva moto atropelli l'accidentat i saltant a la cuneta, per a què els deu motoristes que et segueixen tenguin temps d'esquivar-lo també?

**37) Personal – instrumental**

Ets el líder d'un petit grup de soldats. Torneu d'una missió en territori enemic quan un dels teus homes cau en una trampa que li engrona la seva cama, i li fa una ferida molt greu. No pots alliberar-lo sense que mori, però si l'abandoneu les tropes enemigues el torturaran fins que reveli la posició d'un important campament aliat. Després atacaran l'esmentat campament i mataran els deu homes que composen l'escamot establert allà. El soldat et suplica que no l'abandonis, però les tropes enemigues s'apropen i és perillós quedar-vos per a tu i els teus homes perquè heu esgotat la munició.

Si tu mateix apunyales el soldat atrapat evitaràs que sigui torturat i reveli la informació que portaria a la mort dels deu soldats aliats. Això el matarà però salvaràs els deu membres de l'escamot.

Impedeixes que el soldat atrapat sigui torturat apunyalant-lo tu mateix per a què no reveli la localització dels deu integrants de l'escamot aliat?

**38) Impersonal – Instrumental**

Ets el líder d'un petit grup de soldats. Torneu d'una missió en territori enemic quan un dels teus homes cau en una trampa que li engrona la seva cama, i li fa una ferida molt greu. No pots alliberar-lo sense que mori, però si l'abandoneu les tropes enemigues el torturaran fins que reveli la posició d'un important campament aliat. Després atacaran l'esmentat campament i mataran els deu homes que composen l'escamot establert allà. El soldat et suplica que no l'abandonis, però les tropes enemigues s'apropen i és perillós quedar-vos per a tu i els teus homes perquè heu esgotat la munició.

Si assenyales la posició del soldat atrapat amb una bengala, l'àrea serà bombardejada i així evitaràs que el soldat sigui torturat i reveli la informació que portarà a la mort de l'escamot aliat. Això el matarà però salvaràs els deu integrants de l'escamot.

Impedeixes que el soldat atrapat sigui torturat programant el bombardeig de la zona on està per a què no reveli la localització dels deu soldats aliats?

**39) Personal – instrumental**

Ets celador/a en un hospital durant el torn de nit. Descobreixes que s'ha vessat un producte químic en una habitació amb sis pacients. Aquest producte és altament tòxic i si es deixa en terra s'evapora creant un gas verinós que matarà a tots els pacients de l'habitació. No es pot fregar amb tovalloles o baietes convencionals. En canvi, la pell humana l'absorbeix ràpidament.

Si col·loques un dels pacients sobre el producte la seva pell l'absorbirà i així evitaràs que s'evapori. Aquest pacient morirà però salvaràs els altres cinc de l'habitació.

Evites l'evaporació tòxica col·locant un pacient sobre el producte per a què no s'intoxiquin els altres cinc pacients?

**40) Impersonal – accidental**

Ets celador/a en un hospital durant el torn de nit. Descobreixes que s'ha vessat un producte químic en una habitació amb cinc pacients. Aquest producte és altament tòxic i si es deixa en terra s'evapora creant un gas verinós que matarà a tots els pacients de l'habitació. No es pot fregar amb tovalloles o baietes convencionals.

Si actives el sistema de ventilació el gas verinós serà extret de l'habitació. Però acabarà en una sala del pis superior on hi ha un altre pacient que no pots evacuar a temps. Això matarà a aquest pacient, però salvarà els altres cinc.

Extreus el gas de l'habitació activant el sistema de ventilació, cosa que farà que acabi a la sala de dalt on hi ha un pacient, per a què no s'intoxiquin els altres cinc pacients?

**41) Personal – instrumental**

Estàs dins d'un bot de rems amb un guia turístic en un llac d'Alaska. Tres nens han caigutel seu bot aprop teu i ara corren perill de morir congelats. Rescateu els nens però un d'ells pesa molt i si el mantens a bord segur que us enfonsareu i tots morireu. Ni tu ni el guia podeu abandonar el bot perquè tu estàs remant i el guia està reanimant a un dels nens.

Si tires per la borda el nen feixuc sereu capaços d'arribar a la vorera. Això el matarà, però salvaràs els altres dos.

Elimines pes de la barca tirant al nen feixuc per a poder arribar a la vorera amb els altres dos nens?

**42) Impersonal- accidental**

Estàs dins d'un bot de rems amb un guia turístic en un llac d'Alaska. Tres nens han caigut del seu bot aprop teu i ara corren perill de morir congelats. Després de rescata dos nens te n'adones que l pes d'aquest tercer nen causarà que s'esfonsi la barca i tots morireu. Ni tu ni el guia podeu abandonar el bot perquè tu estàs remant i el guia està fent reanimant a un dels nens.

Si remes cap a la vorera deixant el tercer nen a l'aigua evitaràs que s'esfonsi la barca. Això el matarà, però salvaràs els altres dos.

Evites l'enfonsament de la barca deixant un nen enrera, cosa que farà que es congeli, per a poder arribar a la vorera amb els altres dos nens?

**43) Personal – instrumental**

Treballes en una mina. L'única sortida és pujar en uns cubicles que pengen d'un cable elevador on hi caben onze miners. Heu acabat la jornada i tots ascendiu a la superfície en els cubicles. Mentre esperes torn, t’adones que el cable que els subjecta és a punt de trencar-se.

Si volques el darrer cubicle, el miner que l'ocupa caurà. Així reduiràs prou la càrrega per a què el cable aguanti, i els altres deu miners arribin a la sortida. Això matarà al miner del cubicle volcat però salvarà als altres deu.

Redueixes la càrrega del cablevolcant el darrer cubicle, fent caure un miner, per a què els altres deu arribin a la sortida?

**44) Impersonal- instrumental**

Treballes en una mina. L'única sortida és pujar en uns cubicles que pengen d'un cable elevador i que poden acomodar onze miners. Heu acabat la jornada i tots ascendiu a la superfície en els cubicles. Mentre esperes torn, t’adones que el cable que els subjecta és a punt de trencar-se.

Si prems el botó d'emergència s'amollarà el darrer cubicle del cable. Així es reduïrà prou pes per a què el cable aguanti els cubicles dels altres deu miners. Això matarà el miner del cubicle desenganxat però salvarà als altres deu.

Redueixes la càrrega del cable per a què no es trenqui, prement el botó que amollarà el darrer cubicle i que els altres deu miners arribin a la sortida?

**45) Personal – accidental**

Ets un/a electricista contractat/da per arreglar un problema a casa d'un ric magnat de l'acer que està malalt terminal per un càncer. Recentment s'ha anunciat que la seva gran fortuna l'herederà a l'hospital infantil local. Saps que hi ha 109 nens en aquest hospital que podrien salvar-se amb aquests diners, però només si arriben immediatament.

Si asfixies el magnat mentres dorm tapant la seva boca i nas amb les teves mans enguantades avançaràs la donació vital per als nens. Això matarà al magnat, però salvaràs les vides de 109 nens.

Mates aquest home asfixiant-lo per a que la donació arribi als 109 nens malalts a temps?

**46) Impersonal-Instrumental**

Ets un/a electricista contractat/da per arreglar un problema a casa d'un ric magnat de l'acer que està malalt terminal per un càncer. Recentment s'ha anunciat que la seva gran fortuna l'herederà a l'hospital infantil local. Saps que hi ha 109 nens en aquest hospital que podrien salvar-se amb aquests diners, però només si arriben immediatament

Si crees un curt-circuit enlloc d'arreglar el problema elèctric, el suport vital del magnat fallarà i avançaràs la donació pels nens. Això matarà al magnat, però salvaràs les vides de 112 nens.

Amates aquest magnat fent que falli el suport vital per a que la donació arribi als 112 nens malalts a temps?

47) **Personal – instrumental**

Ets oficial de l'exèrcit durant una guerra. Els teus soldats han trobat un espia d'alt rang atrapat i ferit en un edifici parcialment esfondrat. T'han informat per ràdio que els soldats enemics pretenen fer un atac sorpresa sobre una de les ciutats properes, matant milers de civils que hi viuen, si tu i els teus soldats no ho impediu. Segur que l'espia sap quina ciutat serà atacada però l'has interrogat i no t'ho diu. No durarà molt de temps viu per les seves ferides.

Si el tortures, l'espia revelarà la informació que et permetrà salvar la ciutat. La tortura el matarà però salvaràs a milers de civils.

Fas que l'espia et reveli la informació torturant-lo per a poder impedir l'atac sobre milers de civils?

**48) Impersonal-Instrumental**

Ets oficial de l'exèrcit durant una guerra. Els teus soldats han trobat un espia d'alt rang atrapat i ferit en un edifici parcialment esfondrat. T'han informat per ràdio que els soldats enemics pretenen fer un atac sorpresa sobre una de les ciutats properes, matant milers de civils que hi viuen si tu i els teus soldats no ho impediu. Segur que l'espia sap quina ciutat serà atacada però l'has interrogat i no t'ho diu. No durarà molt de temps viu per les seves ferides. Les rates han començat a mossegar-lo i ell agonitza.

Si permets que les rates continüin aquesta tortura, l'espia revelarà la informació que et permetrà salvar la ciutat. Això el matarà però salvaràs a milers de civils.

Fas que l'espia et reveli la informació deixant que les rates segueixin torturant-lo per a poder impedirl'atac sobre milers de civils?

**49) Personal – Others Beneficial – Inevitable (instrumental)**

Ets metge/ssa. Un dels teus pacients, que ha estat diagnosticat de SIDA, és a punt de rebre una transfusió abans de donar-li l'alta de l'hospital. T'ha confessat, sota la confidencial relació metge-pacient, que té intenció de contagiar al major nombre de persones possible, començant aquesta mateixa tarda en sortir de l'hospital. No tens forma legal d'aturar-lo perquè la policia no pot fer res en base ana informació protegida.

Si contamines la seva transfusió amb un verí irrastrejable evitaràs que pugui infectar a altres. Això el matarà però salvaràs a molts.

Evites el contagi de SIDA enverinant al pacient per a què no infecti a altres persones?

**50) Impersonal – Others Beneficial – Inevitable (instrumental)**

Ets metge/ssa. Un dels teus pacients, que ha estat diagnosticat de SIDA, és a punt de rebre una transfusió abans de donar-li l'alta de l'hospital. T'ha confessat, sota la confidencial relació metge-pacient, que té intenció de contagiar al major nombre de persones possible, començant aquesta mateixa tarda en sortir de l'hospital. No tens forma legal d'aturar-lo perquè la policia no pot fer res en base a una informació protegida.

Si canvies el seu grup sanguini a la base de dades de l'hospital rebrà la sang equivocada i evitaràs que contagïi a moltes persones. Això el matarà però salvaràs a molts.

Evites el contagi de SIDA canviant el grup sanguini del pacient per a que no infecti a altres persones?

**51) Personal – Others Beneficial – Avoidable (instrumental)**

Estàs negociant amb un home trastornat i armat en el terrat d'un edifici. A baix hi ha una multitud de gent en el festival d'art i música de la ciutat. Ell afirma que dispararà a la multitud i no pots convèncer-lo que no ho faci. La multitud desconeix l'amenaça i no tens forma d'avisar-los. Des d'aquesta posició, ell podria matar una dotzena de persones sense comptar les que es veurien afectades pel caos que se produciría alen intentar posar-se fora de perill.

Si corres i l'empenys, caurà del terrat i no podrà disparar a la multitud. Això el matarà però salvaràs moltes vides.

Evites el caos llençant a l'home del terrat per a que no dispari a la multitud?

**52) Impersonal – Others Beneficial – Avoidable (instrumental)**

Estàs negociant amb un home trastornat i armat en el terrat d'un edifici. A baix hi ha una multitud de gent en el festival d'art i música de la ciutat. Ell afirma que dispararà a la multitud i no pots convèncer-lo que no ho faci. La multitud desconeix l'amenaça i no tens forma d'avisar-los. Des d'aquesta posició, ell podria matar una dotzena de persones sense comptar les que es veurien afectades pel caos que es produïria en intentar posar-se fora de perill.

Si avises a la policia que la negociació ha fracassat li dispararan, evitant el tiroteig sobre la multitud. Això matarà a l'home trastornat, però es salvaran moltes vides.

Evites el caos fent que la policia dispari a l'home per impedir que ell crivilli a la multitud?
