# Supplementary material for: Moral judgment reloaded: a moral dilemma validation study
Source: Front Psychol. 2014 Jul 1;5:607. doi: 10.3389/fpsyg.2014.00607 (PMC4077230; doi:10.3389/fpsyg.2014.00607)
Supplement: Supplementary file 6 [file DataSheet6.DOC]

**Danish**

**DILEMMAER**

**1) Personal - Instrumental**

Du og fem andre personer er fanget i en brændende bygning. Der er kun én nødudgang i kan slippe ud igennem allesammen. Men den er blokeret af brændende ting. Du ser at en såret person forsøger at kravle gennem et lille hul nederst i nødudgangen. Du og de ​​andre fem personer bag dig har ikke tid til at gøre det samme.

Hvis i bruger den sårede person til at rage de brændene ting væk kan i slippe ud. Dette vil helt sikkert dræbe den sårede person, men hvis i ikke gør det, dør du og de ​​fem personer bag dig.

Bruger du den sårede person til at rage de brændene ting væk så du og de andre fem personer kan slippe ud?

**2) Impersonal- Accidental**

Du og fem andre personer er fanget i en brændende bygning. Der er kun én nødudgang, hvorigennem i alle kan slippe ud. Men den er blokeret af brændende ting. Du ser at en såret person forsøger at kravle gennem et lille hul nederst i ​​ nødudgangen. Du og de ​​andre fem personer bag dig har ikke tid til at gøre det samme.

Hvis du aktiverer nødsystemet vil det fjerne ilten fra gangen og dette vil slukke ilden. Den sårede person vil blive kvalt og dø, men hvis du ikke gør det, vil du og de ​​fem personer bag dig dø.

Aktiverer du nødsystemet, hvilket vil resultere i at den sårede person ikke får nok ilt, for at slukke ilden så du og de andre fem personer kan slippe ud?

**3) Personal – accidental**

Fjendtlige soldater har indtaget din landsby og har fået ordre om at dræbe alle civilister over to år. Du og 10 naboer har gemt jer i to rum i kælderen af et stort hus. I hører soldaternes stemmer ovenpå. De er kommet for at lede efter værdigenstande. Din baby begynder at græde højlydt.

Hvis du lægger din hånd over babiens mund undgår du at gråden tilkalder soldaternes opmærksomhed. De ville nok forskåne din babys liv, men dræbe dig og de ​​andre. Hvis du lader din hånd være på din babys mund vil den blive kvalt, men hvis Du ikke gør det, dør du og de andre 10.

Dæmper du gråden med din hånd, hvilket gør at din baby ikke får nok ilt, så soldaterne ikke finder dig og de 10 andre?

**4) Impersonal- accidental**

Fjendtlige soldater har indtaget din landsby og har fået ordre om at dræbe alle civilister over to år. Du og 10 naboer har gemt jer i to rum i kælderen af et stort hus. I hører soldaternes stemmer ovenpå. De er kommet for at lede efter værdigenstande. Din baby begynder at græde højlydt.

Hvis du aktiverer en larmende kedel vil denne dæmpe lyden. Dog ville kedlen blive meget varm. Denne varme er ubehagelig for voksne og børn, men dødbringende for babyer.
Dette vil sikkert dræbe din baby, men hvis Du ikke gør det, dør du og de andre 10.

Dæmper du gråden ved at tænde for kedlen, hvilket gør at det bliver for varmt for din baby, så soldaterne ikke finder dig og de 10 andre?

**5) Personal – accidental**

Du er en del af besætningen på en u-båd, der sejler under et stort isbjerg. En eksplosion har skadet skibet, såret adskillige af dine kollegaer og kollapseret den eneste adgang mellem den øvre og nedre del af skibet. Du og 10 overlevende er fanget i den øverste del, som ikke har nok ilt til jer alle til i når overfladen. Én kollega ligger bevidstløs i den nederste sektion, hvor der er ilt nok.

Hvis du åbner en nødluge mellem de to sektioner, vil luft strømme ind til jer. Dog vil lugen falde ned på kollegaen og dræbe ham, men dette vil rede dig og de andre 10 overlevende.

Åbner du lugen, som vil falde ned på kollegaen, så du og de ti andre kan få nok ilt?

**6) Impersonal- accidental**

Du er en del af besætningen på en u-båd, der sejler under et stort isbjerg. En eksplosion har skadet skibet, såret adskillige af dine kollegaer og kollapseret den eneste adgang mellem den øvre og nedre del af skibet. Du og 10 overlevende er fanget i den øverste del, som ikke har nok ilt til jer alle til i når overfladen. En kollega ligger bevidstløs i den nederste sektion, hvor der er ilt nok.

Hvis du trykker på nødkontakten vil en luge mellem de to sektioner blive åbnet, og ilt strømme ind til jer. Dog vil lugen falde ned på kollegaen og dræbe ham, men det vil rede dig og de andre 10 overlevende.

Trykker du på nødkontakten for at åbne lugen, der vil falde ned på kollegaen, så dig og de ti andre får nok ilt?

**7) Personal** – **instrumental**

Du og ti andre dyggere fra et team af De Forenede Nationer deaktiverer undersøiske miner fra Anden Verdenskrig. Et teammedlem er kommet til skade og blodet har tiltrukket hajer. Du har et undervandsgevær, men kun én harpun, og der er mange hajer. Den blødende dykker svømmer mod det sidste sikkerhedsbur og vil nå det før dig og de andre. Hajerne der har fulgt blodet er kommet for tæt på dig og de andre dykkere, for at i kan undslippe.

Hvis du skyder på den såredede dykker, vil dette dræbe ham og hajerne vil stoppe op og spise ham, men så kan du og de andre ti komme i sikkerhed.

Skyder du dykkeren så hajerne stopper op og spiser ham, så du og de ti andre kan undslippe?

**8) Impersonal-Instrumental**

Du og ti andre dyggere fra et team af De Forenede Nationer deaktiverer undersøiske miner fra Anden Verdenskrig. Et teammedlem er kommet til skade og blodet har tiltrukket hajer. Du har et undervandsgevær, men kun én harpun, og der er mange hajer. Den blødende dykker svømmer mod det sidste sikkerhedsbur og vil nå det før dig og de andre. Hajerne der har fulgt blodet er kommet for tæt på dig og de andre dykkere, for at i kan undslippe.

Hvis du detonerer en mine med en fjernbetjening vil eksplosionen dræbe den sårede dykker og hajerne vil stoppe op og spise ham, men så kan du og de andre ti komme i sikkerhed.

Udløser du bomben med fjernbetjeningen så hajerne stopper op og spiser den sårede dygger, så du og de ti andre kan undslippe?

**9) Personal – instrumental**

Du arbejder på et børnehjem i et land i krig. Rebeller har overtaget regeringen og dræber alle, der ikke støtter deres regime. Desuden gennemfører de en folkedrabs kampagne mod en bestemt etnisk gruppe. Du og elve forældreløse børn har ikke understøttet rebellernes opstand og du har et barn af denne etniske gruppe i dit børnehjem. Der er ingen chance for at du og de ​​elve børn kan undslippe og det er sikkert at soldaterne vil dræbe jer alle.

Hvis du kvæler dette ene barn med en pude mens det sover vil rebellerne tro at du støtter deres regime og dette vil redde dit og de andre ti børns liv.

Får du rebellerne til at tro at du støtter deres regime ved at kvæle dette barn, så rebellerne vil forskåne dit og de andre ti børns liv?

**10) Impersonal – Instrumental**

Du arbejder på et børnehjem i et land i krig. Rebeller har overtaget regeringen og dræber alle, der ikke støtter deres regime. Desuden gennemfører de en folkedrabs kampagne mod en bestemt etnisk gruppe. Du og elve forældreløse børn har ikke understøttet rebellernes opstand og du har et barn af denne etniske gruppe i dit børnehjem. Der er ingen chance for at du og de ​​elve børn kan undslippe og det er sikkert at soldaterne vil dræbe jer alle.

Hvis du beordrer en sygeplejerske til at give barnet en dødelig overdosis af sovepiller vil rebellerne tro at du støtter deres regime. Dette vil redde dit og de andre ti børns liv.

Får du rebellerne til at tro at du støtter deres regime ved at beordre at sygeplejersken giver barnet en overdosis så rebellerne vil forskåne dit og de andre ti børns liv?

**11) Personal – instrumental**

Du er en frivillig sygeplejer/ske i en afrikansk landsby. En mand fra nabolandsbyen er blevet inficeret med Ebola-virusen, som er meget smitsom, uhelbredelig og næsten altid dødelig. Imod prognosen har han overlevet en måned, hvilket betyder at han er immun overfor de dødelige virkninger af virusen. Men han antager fejlagtigt, at dit lægecenter kan helbrede ham fuldstændigt. Du ser at han nærmer sig, og du ved, at hvis han kommer ind i landsbyen vil han smitte dig og de øvrige 119 beboere, som, i modsætning til ham, vil dø.

Hvis du skyder på ham med lægecenterets pistol vil dette dræbe ham, men forhindre ham i at smitte dig og de øvrige 119 beboere af landsbyen med virusen.

Forhindrer du manden i at komme ind i landsbyen ved at bruge centerets pistol, saa han ikke kan smitte dig de øvrige 119 beboere af landsbyen?

**12) Impersonal-Instrumental**

Du er en frivillig sygeplejer/ske i en afrikansk landsby. En mand fra nabolandsbyen er blevet inficeret med Ebola-virusen, som er meget smitsom, uhelbredelig og næsten altid dødelig. Imod prognosen har han overlevet en måned, hvilket betyder at han er immun overfor de dødelige virkninger af virusen. Men han antager fejlagtigt, at dit lægecenter kan helbrede ham fuldstændigt. Du ser at han nærmer sig, og du ved, at hvis han kommer ind i landsbyen vil han smitte dig og de øvrige 119 beboere, som, i modsætning til ham, vil dø.

Hvis du informerer landsbyrådet vil de skyde på ham. Dette vil dræbe ham, men det er den eneste måde at forhindre at han smitter dig og resten af ​​landsbyen.

Forhindrer du manden i at komme ind i landsbyen ved at informere landsbyrådet, så han ikke kan smitte dig de øvrige 119 beboere af landsbyen?

**13) Personal – instrumental**

Du er en læge alene i en redningshelikopter under en frygtelig oversvømmelseskatastrofe. Du har reddet otte kritisk sårede og holder dem i live. Pludselig advarer piloten dig at der er en fejl i motoren og at helikopteren må frigøre vægt eller i vil styrte ned og alle vil dø. Du kan ikke selv forlade helikopteren fordi de sårede har brug for din hjælp og det tunge mediciniske udstyr er skruet fast.

Hvis du skubber en af de sårede ud, vil dette frigøre nok vægt. Dette vil sikkert dræbe denne person, men vil redde dig og de andre otte personer ombord.

Frigør du vægt ved at skubbe en af de sårede ud, så helicopteren kan fortsætte luften med dig og de otte sårede?

**14) Impersonal-Instrumental**

Du er en læge alene i en redningshelikopter under en frygtelig oversvømmelseskatastrofe. Du har reddet otte kritisk sårede og holder dem i live. Pludselig advarer piloten dig at der er en fejl i motoren og at helikopteren må frigøre vægt eller i vil styrte ned og alle vil dø. Du kan ikke selv forlade helikopteren fordi de sårede har brug for din hjælp og det tunge mediciniske udstyr er skruet fast.

Hvis du skærer ræbet igennem som den sidste sårede er ved at blive firet op med, vil dette frigøre nok vægt til at holde helikopteren i luften. Dette vil dræbe denne person, men redde dig og de andre otte.

Frigør du vægt ved at gennemskære denne persons ræb så helicopteren kan fortsætte i luften med dig og de otte sårede?

**15) Personal – accidental**

Du er en ingeniør på den internationale rumstation ISS. Pludselig går der ild i laderummet. Det automatiske sikkerhedssystem virker ved at åbne ydrelugen hvilket eliminerer ilten og sådan slukker ilden. Men det virker kun når den indre luge er lukket, og der er stadig en mekaniker bag lugen. Du ser, at han ikke har tid til at tage den tunge rumdragt af og at han vil komme til at side fast hvis han prøver at komme ind gennem lugen. Dette vil få ilden til at brede sig og dræbe jer alle.

Hvis du skubber mekanikeren væk fra lugen og forsegler den, vil sikkerhedsystemet gå i gang. Dette vil sende mekanikeren ud i rummet og dræbte ham, men det vil redde dig og de andre ti.

Slukker du ilden ved at lukke lugen hvilket resulterer i at mekanikeren bliver sendt ud i rummet, så ilden ikke når dig og de andre ti astronauter?

**16) Impersonal – accidental**

Du er en ingeniør på den internationale rumstation ISS. Pludselig går der ild i laderummet. Det automatiske sikkerhedssystem virker ved at åbne ydrelugen hvilket eliminerer ilten og sådan slukker ilden. Men det virker kun når den indre luge er lukket, og der er stadig en mekaniker bag lugen. Du ser, at han ikke har tid til at tage den tunge rumdragt af og at han vil komme til at side fast hvis han prøver at komme ind gennem lugen. Dette vil få ilden til at brede sig og dræbe jer alle.

Hvis du trykker på en nødkontakt der forsegler lugen inden mekanikeren når den, aktiveres sikkerhedsystemet og ilden slukkes. Dette vil sende mekanikeren ud i rummet og dræbte ham, men det vil redde dig og de andre ti.

Slukker du ilden ved at trykke på nødkontakten der forsegler lugen hvilket resulterer i at mekanikeren bliver sendt ud i rummet, så ilden ikke når dig og de andre ti astronauter?

**17) Personal – instrumental**

Du er inspektør på et atomkraftværk. Du og direktøren inspicerer netop værket da en af kølrørene overhedes. Nød-kølesystemet svigter og en kædereaktion er ved at gå i gang som vil resultere i kernefusion. Sker dette, vil den frigivne stråling være dødelig for dig og beboerne af en nærliggende by.

Hvis du skubber direktøren ned på kølrørene vil dette fjerne nok varme til at stoppe kædereaktionen. Dette vil dræbe direktøren, men det vil redde dig og beboerne af den nærliggende by.

Nedkøler du rørene ved at skubbe direktøren ned på dem, så du og beboerne af den nærliggende by undgår strålingen?

**18) Impersonal – accidental**

Du er inspektør på et atomkraftværk. Du og direktøren inspicerer netop værket da en af kølrørene overhedes. Nød-kølesystemet svigter og en kædereaktion er ved at gå i gang som vil resultere i kernefusion. Sker dette, vil den frigivne stråling være dødelig for dig og beboerne af en nærliggende by.

Hvis du manuelt frigør flydende kvælstof ind i systemet vil dette fjerne nok varme til at stoppe kædereaktionen. Men der er en medarbejder der vil blive lukket inde i systemets komandorum. Dette vil dræbe ham, men det vil redde dig og beboerne af den nærliggende by.

Nedkøler du rørene ved at manuelt frigøre flydende kvælstof ind i systemet, så du og beboerne af den nærliggende by undgår strålingen?

**19) Personal – instrumental**

Du er en sprængstoff ekspert i en virksomhed med den opgave at rive en skyskraber ned. Du er ved at undersøge den sidste sprængladning, da du får øje på en fyr der er ved at detonere en af sprængladningerne uden at være klar over det. Eksplosionen ville resultere i en ukontrolleret kollaps af bygningen over dig, drengen og mængden af tilskuere. Han står flere etager under dig, og kan ikke høre dig.

Hvis du lader en tung beton blok falde ned på hans hoved vil dette sikkert dræbe ham, men det vil forhindre eksplosionen og redde dig og tilskurermængden.

Stopper du drengen ved at lade beton blokken falde ned over hans hoved så bygningen ikke kollapser ukontrolleret af over dig og tilskuerene?

**20) Impersonal-Instrumental**

Du er en sprængstoff ekspert i en virksomhed med den opgave at rive en skyskraber ned. Du er ved at undersøge den sidste sprængladning, da du får øje på en fyr der er ved at detonere en af sprængladningerne uden at være klar over det. Eksplosionen ville resultere i en ukontrolleret kollaps af bygningen over dig, drengen og mængden af tilskuere. Han står flere etager under dig, og kan ikke høre dig.

Hvis du genopretter elektriciteten til bygningen vil drengen få et elektrisk stød fordi han netop rører ved nogle kabler. Dette vil dræbe ham, men vil forhindre eksplosionen og redde dig og tilskurermængden.

Stopper du drengen ved at genoprette elektriciteten så bygningen ikke kollapser ukontrolleret af over dig og tilskuerene?

**21) Personal – instrumental**

Du arbejder i byggebranchen. Du og dit team arbejder på stilladset af en skyskraber. Pludselig falder stilladset delvist sammen. Du og flere kammerater holder fast i en tværbjælke, men i vejer for meget og den er ved at briste. En kammerat ved siden af dig på tværbjælken rutscher ned og griber fat din arm. Stiladset er lige ved at vige.

Hvis du sparker din kammerant flere gange i maven og brystet vil han løsne sit greb og falde. Dette vil dræbe ham, men det vil også frigøre nok vægt til at resten af jer kan komme i sikkerhed før sammenbruddet.

Frigør du vægten ved at sparke din kamerat til han falder, så du og resten af holdet kan blive hængene?

**22) Impersonal-Instrumental**

Du arbejder i byggebranchen. Du og dit team arbejder på stilladset af en skyskraber. Pludselig falder stilladset delvist sammen. Du og flere kammerater holder fast i en tværbjælke, men i vejer for meget og den er ved at briste. En kammerat ved siden af dig på tværbjælken rutscher ned og griber fat i tværbjælken overfor. Stiladset er lige ved at vige.

Hvis du river stiften ud der holder din kammarats tværbjælke vil denne falde ned og frigøre nok vægt til at resten af jer kan komme i sikkerhed før sammenbruddet. Dette vil dræbe ham, men det vil redde dig og de andre.

Frigør du vægten ved at rive stiften ud af din kammerats tværbjælke så denne falder ned, så du og resten af holdet kan blive hængene?

**23) Personal – accidental**

Du er buschauffør og kører bussen på en børnehaveudflugt til zoologisk have. På vej derhen forårsager en hjulskade at bussen styrter fra en bro ned i en rivende flod. Du og tre børn befinder jer stadig i den synkende bus. Resten er allerede blevet revet med af strømningen. Du tager fat i de to børn tættest på og begynder at svømme mod udgangen. Det tredje barn klamrer sig til dit ben. Du mærker at du ikke er stærk nok til at svømme imod strømmen med alle tre børn.

Hvis du spraller dig fri af det tredje barns vægt og lader ham drukne kan du kæmpe mod strømmen. Dette vil dræbe barnet, men det vil redde dig og de andre to børn.

Frigør du dig af det tredje barn, hvilket vil resultere i at han drukner, så du og de andre to børn kan nå op til overfladen?

**24) Impersonal-accidental**

Du er buschauffør og kører bussen på en børnehaveudflugt til zoologisk have. På vej derhen forårsager en hjulskade at bussen styrter fra en bro ned i en rivende flod. Du og tre børn befinder jer stadig i den synkende bus. Resten er allerede blevet revet med af strømningen. Du tager fat i de to børn tættest på og begynder at svømme mod udgangen men den er blokeret af nogle bussæder.

Hvis du fjerner sæderne ved at skubbe dem ud på gangen frigør du udgangen men forspærrer gangen for det tredje barn hvilket vil resultere i at han drukner. Dette vil dræbe barnet, men det vil redde dig og de andre to børn.

Frigør du udgangen ved at flytte sæderne hvilket vil resultere i at et barn drukner, så du og de andre to børn kan nå op til overfladen?

**(25) Personal – instrumental**

Du læge på et hospital. Du har fem patienter der vil dø hvis de ikke får en organtransplantation med det samme. En ny patient bliver netop inleveret efter en alvorlig bilulykke. Hans tilstand er kritisk og han vil dø hvis han ikke bliver opereret øjeblikkeligt, men du kan nok redde ham med en lang og kompliceret operation.

Hvis du hemmeligt gennemskærer hans halspulsåre under operationen vil denne patient dø, men du vil få organerne til de andre fem patienter. Ulykkesofret vil dø, men det vil redde de øvrige fem patienter.

Opnår du organerne ved at gennemskære halspulsåren på ulykkesoffret så du kan give dem til de andre fem patienter?

**26) Impersonal-Instrumental**

Du læge på et hospital. Du har fem patienter der vil dø hvis de ikke får en organtransplantation med det samme. En ny patient bliver netop inleveret efter en alvorlig bilulykke. Hans tilstand er kritisk og han vil dø hvis han ikke bliver opereret øjeblikkeligt. Under den lange og komplicerede operation ser du hvordan en sygeplejerske er ved at sprøjte en forkert medicin, dødelig i dette sammenhæng.

Hvis du lader sygeplejersken sprøjte medicinen vil det dræbe denne patient, men du vil få organerne til de andre fem patienter. Ulykkesofret vil dø, men det vil redde de øvrige fem patienter.

Opnår du organerne ved at lade sygeplejersken sprøjte medicinen så du kan give dem til de andre fem patienter?

**27) Personal-27) Impersonal – accidental**

Du er en del af et team af havnearbejdere der fastgør kranernes kæder til kontainere på fragtskibe. Du og de ​​andre har netop fastgjort sådanne kæder på en kontainer og er i færd med at kravle op på den for at sikre en korrekt afladning. Pludselig ser du alarmen blinke. Den advarer om at en kæde er ved at briste. Du ser at det er pga to kollegaer der er oppe at slås og får kontaineren til at sveje farligt hen over fem andre kollegaer der står nede på dækket.

Hvis du skubber dem fra hinanden, undgår du at kontaineren styrter ned, men en af de to har ikke sin sikkerhedssele på og vil falde ned. Dette vil dræbe ham, men det vil redde de fem kollegaer på dækket.

Stopper du kontainerens svejen ved at skubbe de to kollegaer hvilket vil gøre at ham uden sikkerhedsele styrter, så den ikke falder ned på de fem kollegaer på dækket?

**28) Impersonal – accidental**

Du er en del af et team af havnearbejdere der fastgør kranernes kæder til kontainere på fragtskibe. Du og de ​​andre har netop fastgjort sådanne kæder på en kontainer og er i færd med at kravle op på den for at sikre en korrekt afladning. Pludselig ser du alarmen blinke. Den advarer om at en kæde er ved at briste. Hvis kæden brister over dækket vil kontaineren falde og begrave fem kollegaer under sig.

Du kan trykke på en sikkerhedknap som vil sende kontaineren tilbage til laderummet. Du og dine kollegaer vil blive hængene i jeres sikkerhedsseler, men der er stadig en kollega nede i laderummet. Han vil blive drabt af den nedstyrtende kontainer, men det vil redde de fem på dækket.

Sender du kontaineren tilbage til laderummet ved at trykke på knappen, hvilket vil gøre at den begraver kollegaen under sig, så den ikke falder ned på de fem kollegaer på dækket?

**29) Personal – accidental**

En epidemi har spræt sig over hele verden og dræbt tusinder. Du er forsker og har udviklet to substandser i laboratoriet. Ved en fejltagelse er begge blevet etiketeret som vaccine. En af dem er virkelig en vaccine der kunne redde tusinder af mennesker, den anden er dødbringende.

Hvis du sprøjter dine to assistenter med hver en substands vil du kunne identificere hvilken der er vaccinen. En af assistenterne vil dø, men du vil kunne redde tusinder af mennesker med vaccinen.

Identificerer du vaccinen ved at sprøjte dine assistenter, hvilket vil forgifte en af dem, så tusinder af mennesker kan blive vaccineret?

**30) Impersonal-accidental**

En epidemi har spræt sig over hele verden og dræbt tusinder. Du er forsker og har udviklet to substandser i laboratoriet. Ved en fejltagelse er begge blevet etiketeret som vaccine. En af dem er virkelig en vaccine der kunne redde tusinder af mennesker, den anden er dødbringende.

Hvis du lader en sygeplejerske sprøjte to af dine patienter med hver en substands vil du kunne identificere hvilken der er vaccinen. En af dem vil dø, men du vil kunne redde tusinder af mennesker med vaccinen.

Identificerer du vaccinen ved at lade sygeplejersken sprøjte dine to patienter, hvilket vil forgifte en af dem, så tusinder af mennesker kan blive vaccineret?

**31) Personal – instrumental**

En førerløs sporvogn i fuld fart er på vej hen mod fem skinnearbejdere, som vil blive kørt ned, hvis vognen fortsætter i den retning. Du står ved siden af ​​sporene, men er for langt væk for at advare dem. Ved siden af ​​dig står der en meget stor fremmed mand.

Hvis du skubber den store fremmede ned på sporene, vil vognen køre af sporene og vil ikke køre de fem arbejdere ned. Dette vil dræbe den fremmede, men du ville redde de fem arbejdere.

Skubber du den fremmede ned på skinnerne, så sporvognen ikke når de fem skinnearbejdere?

**32) Impersonal – accidental**

En førerløs sporvogn i fuld fart er på vej hen mod fem skinnearbejdere, som vil blive kørt ned, hvis vognen fortsætter i den retning. Du står ved siden af ​​sporene, men er for langt væk for at advare dem. Ved siden af ​​dig er der en skiftestang, som kan omdirigere vognen på et sidespor. På dette spor er der kun én arbejder.

Hvis du trækker i skiftestangen vil vognen skifte spor og ikke køre de fem arbejdere over. Dette vil dræbe arbejderen på sidesporet, men redde de fem andre.

Trækker du i skiftestangen så vognen skifter spor og rammer arbejderen på sidesporet, så sporvognen ikke når de andre fem skinnearbejdere?

**33) Personal – instrumental**

Du og en forsknings-kollega har opfundet en ny energikilde, der er billig, ren og sikker. Den er stærk nok til at sætte en stopper på miljøforurening og fattigdom. Men din kollega ønsker at sælge opfindelsen, og du ved, at han har planer om at kontakte potentielle købere senere i dag. Men kun du ved også med sikkerhed at disse vil misbruge opfindelsen som et forfærdeligt våben.

Hvis du forgifter din kollega med en gift i almindeligvis har på laboratoriet, undgår du at opfindelsen falder i de forkerte hænder. Giften vil dræbe ham, men du vil redde hundredevis af mennesker.

Undgår du at opfindelsen falder i de forkerte hænder ved at forgifte din kollega, så opfindelsen ikke kan blive brugt som et forfærdeligt våben mod tusinde mennesker?

**34) Impersonal-Instrumental**

Du og en forsknings-kollega har opfundet en ny energikilde, der er billig, ren og sikker. Den er stærk nok til at sætte en ende på miljøforurening og fattigdom. Men din kollega ønsker at sælge opfindelsen, og du ved, at han har planer om at kontakte potentielle købere senere i dag per email. Men kun du ved også med sikkerhed at disse vil misbruge opfindelsen som et forfærdeligt våben.

Hvis du frigør en brædbar gas i laboratoriet vil denne udløse en explosion når din kollega tænder for sin computer. Explosionen vil dræbe ham, men du undgår at opfindelsen falder i de forkerte hænder og du vil redde hundredevis af mennesker.

Undgår du at opfindelsen falder i de forkerte hænder ved at frigøre gasset, så opfindelsen ikke kan blive brugt som et forfærdeligt våben mod tusinde mennesker?

**35) Personal – instrumental**

Du er en erfaren motorcykelrytter og deltager i et motorcross festival. Du kører netop foran en stor gruppe af andre motorcykelryttere da du ser hvordan en anden rytter oppe foran mister kontrollen over sin maskine. Mens du giver gas for at komme ham til undsætning, indser du at han er ved at vælte hvert sekund. Dette vil medføre en kædereaktion og føre til en massekarambulage, der vil dræbe de ti motorcykelryttere bag dig.

Hvis du tvinger ham ud i rabatten med din motorcykel vil han køre ind i træerne. Ved jeres hastighed vil det dræbe ham, men det ville redde de ti motorcykelryttere bag dig.

Undgår du massekarambulagen ved at tvinge denne billist af vejen så de ti billister bag dig ikke kører ind i ham og falder?

**36) Impersonal-instrumental**

Du er en erfaren motorcykelrytter og deltager i en motorcross festival. Du kører netop foran en stor gruppe af andre motorcykelryttere da du ser hvordan en anden rytter oppe foran mister kontrollen over sin maskine og falder. Du selv kan let undvige ham, men bilisterne bag dig vil støde ind i hinanden i deres forsøg at undgå ham, hvilket vil medføre en dødlig massekarambulage.

Hvis du lader din motorcykel køre ind i den faldene billist og selv hoppe ud i rabatten vil dette alarmere de andre i tide og undgå massekarambulagen. Dette vil dræbe den faldne motorcykelrytter, men du vil redde de ti andre bag dig.

Advarer du de andre ved at lade din motorcykel køre ind i den faldene billist så de ti motorcykelryttere bag dig ikke også kører ind i ham og falder?

**37) Personal – instrumental**

Du er leder af en lille gruppe soldater. I vender netop tilbage fra en mission i fjendens territorium, da en af ​​dine mænd trædder i en fælde. Hans ben er alvorligt såret og sidder fast i fælden. Du kan ikke befri ham uden at dræbe ham, men hvis du efterlader ham vil fjendens tropper finde og torturere ham indtil han afslører gemmestedet af et allieret base camp. Så vil fjenden angribe dettte og dræbe de ti soldater der er stationeret der. Soldaten trygler dig om ikke at efterlade ham, men fjenden nærmer sig og det er farligt for dig og dine mænd at blive.

Hvis du stikker soldaten ned med en kniv undgår du at han bliver tortureret og afslører de oplysninger, som vi føre til at fjenden finder de allierede. Dette vil dræbe ham, men du vil redde de ti allierede soldater.

Undgår du at soldaten bliver totureret ved at stikke ham ned selv så han ikke afslører de 10 allierede soldaters lejr?

**38) Impersonal – Instrumental**

Du er leder af en lille gruppe soldater. I vender netop tilbage fra en mission i fjendens territorium, da en af ​​dine mænd trædder i en fælde. Hans ben er alvorligt såret og sidder fast i fælden. Du kan ikke befri ham uden at dræbe ham, men hvis du efterlader ham vil fjendens tropper finde og torturere ham indtil han afslører gemmestedet af et allieret base camp. Så vil fjenden angribe dettte og dræbe de ti soldater der er stationeret der. Soldaten trygler dig om ikke at efterlade ham, men fjenden nærmer sig og det er farligt for dig og dine mænd at blive.

Hvis du angiver den sårede soldats position til atelleri computeren, vil området blive bombarderet og soldaten vil dø uden at blive tortureret og uden at afsløre de oplysninger, som vil føre fjenden til det allierede basecamp. Dette vil dræbe ham, men du vil redde de ti allierede soldater.

Undgår du at soldaten bliver totureret ved at angive hans position til atellericomputeren så han ikke afslører de 10 alliierede soldaters lejr?

**39) Personal – instrumental**

Du er nattevagt på et hospital. En kemikalie er blevet spildt på et værelse med seks patienter. Kemikalien er meget giftig fordi den fordamper når den kommer i kontakt med ilt. Men ved fordampningen skabes der et giftigt gas der vil dræbe alle patienterne i rummet. Man kan ikke fjerne kemikalien med almindelige håndklæder eller klude. Men den menneskelige hud absorberer den hurtigt.

Hvis du lægger en af patienterne ned på gulvet på kemikalien, så undgår du at den fordamper. Denne patient vil dø, men du vil redde de andre fem.

Lægger du en patient på kemikalien for at forhindre at denne fordamper så de andre fem patienter ikke bliver forgiftet?

**40) Impersonal – accidental**

Du er nattevagt på et hospital. En kemikalie er blevet spildt på et værelse med seks patienter. Kemikalien er meget giftig fordi den fordamper når den kommer i kontakt med ilt. Ved fordampningen skabes der et giftigt gas der vil dræbe alle patienterne i rummet. Man kan ikke fjerne kemikalien med almindelige håndklæder eller klude.

Hvis du aktiverer ventilationssystemet vil gassen blive suget ud af rummet, men den vil ende på et værelse ovenpå, hvor der ligger en anden patient som du ikke vil kunne redde ud af værelset i tide. Denne patient vil dø, men du vil redde de andre fem.

For du gassen suget ud af værelset ved at aktivere ventilationssystemet, som vil omdirigere gassen til en anden patients værelse, så de fem patienter ikke bliver forgiftet?

**41) Personal – instrumental**

Du roer en robåd med en tourguide på en sø i Alaska. Tre børn er kænteret med deres båd, og er nu i fare for at fryse ihjel. Mens du begynder at ro mod dem indser du at jeres båd ikke vil kunne bære din, guidens og de tre børns vægt. I redder ​​børnene men en af dem er meget tung og hvis han bliver ombord vil båden kentre og alle tre børn vil dø. Du og guiden kan ikke forlade båden fordi du roer båden og han er ved at genoplive et af børnene.

Hvis du skubber det tunge barn over bord, vil i kunne nå bredden med de andre to børn. Dette vil dræbe dette ene barn, men du vil redde de andre to.

Letter du bådens vægt ved at skubbe det tunge barn over bord, så i kan nå bredden med de andre to?

**42) Impersonal- accidental**

Du roer en robåd med en tourguide på en sø i Alaska. Tre børn er kænteret med deres båd, og er nu i fare for at fryse ihjel. Mens du begynder at ro mod dem indser du at jeres båd ikke vil kunne bære din, guidens og de tre børns vægt. I redder ​​to af børnene men et barn er stadig i vandet. Hvis i også tager dette barn ombord vil båden kæntre og alle tre børn vil dø. Du og guiden kan ikke forlade båden fordi du roer båden og han er ved at genoplive et af børnene.

Hvis du roer hurtigt mod bredden med de andre to børn og lader det tredje blive i vandet undgår du at jeres båd kentrer. Dette vil dræbe dette ene barn, men du vil redde de andre to.

Undgår du at jeres båd kentrer ved at efterlade det tredje barn i vandet, hvilket vil resultere i at han fryser ihjæl, så i kan nå bredden med de andre to børn?

**43) Personal – instrumental**

Du er minearbejder dybt nede i jorden. For at komme ud skal man bestige en af kabinerne på en simpel kabelelevator. Der kan højst være ​​tre personer i en kabine. Arbejdsdagen er ovre og alle er på vej op i kabinerne. Mens du venter på din, ser du at det kabel der holder alle kabinerne er ved at briste. Der er netop 13 minearbejdere i kabinerne.

Hvis du vælter den sidste kabine i hvilken der kun står én minearbejder vil du frigøre nok vægt til at holde de andre kabiener i luften. Dette vil dræbe minearbejderen i den sidste kabine, men du vil redde de 12 andre fra deres sikre død.

Frigør du vægt ved at vælte den sidste minearbejders kabine så de 12 andre minearbejdere kan nå op til overfladen?

**44) Impersonal- instrumental**

Du er minearbejder dybt nede i jorden. For at komme ud skal man bestige en af kabinerne på en simpel kabelelevator. Der kan højst være ​​tre personer i en kabine. Arbejdsdagen er ovre og alle er på vej op i kabinerne. Mens du venter på din, ser du at det kabel der holder alle kabinerne er ved at briste. Der er netop 10 minearbejdere i kabinerne.

Hvis du trykker en nødalarm bliver den sidste kabine automatisk væltet hvilket frigører nok vægt til at holde de andre kabiner i luften. Der står én minearbejder i denne sidste kabine og nødalarmen vil dræbe ham, men du vil redde de 10 andre fra deres sikre død.

Frigør du vægt ved at trykke nødalarmen hvilket vil vælte den sidste minearbejders kabine, så de 10 andre minearbejdere kan nå op til overfladen?

**45) Personal – accidental**

Du er en elektrikker som er blevet hyret til at reparere et problem med en ledning hos en rig storindustriel, der er ved at dø af kræft. Det er for nylig blevet offenliggjort, at hans formue vil blive doneret til et lokalt børnehospital når han dør. Du ved, at der er 109 børn på hospitalet der kunne blive reddet med disse penge, men de kan ikke vente meget længere, fordi de er på randen af ​​døden.

Hvis du kvæler manden mens han sover ved at dække hans mund og næse med dine behandskede hænder, vil du påskønne donationen til børnene. Dette vil dræbe den storindustrielle, men redde de 109 børn.

Påskønner du donationen ved at kvæle denne mand, så de 109 syge børn får de penge, de har brug for?

**46) Impersonal-Instrumental**

Du er en elektrikker som er blevet hyret til at reparere et problem med en ledning hos en rig storindustriel, der er ved at dø af kræft. Det er for nylig blevet offenliggjort, at hans formue vil blive doneret til et lokalt børnehospital når han dør. Du ved, at der er 112 børn på hospitalet der kunne reddes med disse penge, men de kan ikke vente meget længere, fordi de er på randen af ​​døden.

Hvis du kortslutter ledningerne i stedet for at reparere dem, vil dette slukke mandens åndeapparat og du vil påskønne donationen til børnene. Dette vil dræbe den storindustrielle, men redde de 112 børn.

Påskønner du donationen ved at kortslutte ledningerne hvilket vil slukke mandens åndeapparat, så de 112 syge børn får de penge, de har brug for?

**47) Personal – instrumental**

Du officer i hæren i krig. Dine soldater har fundet en vigtig spion som sidder fast i en kollapseret bygning og desuden er alvorligt såret. Over radioen er du blevet informeret om at fjendens soldater planlægger et overraskelsesangreb på en nærliggende by, men du ved ikke om hvilken by det drejer sig. Angrebet vil dræbe tusinde civilister hvis du og dine soldater ikke når at stoppe det. Spionen ved, hvilken by det er. Du har forhørt ham men han vil ikke fortælle dig det. Han dør snart af sine kvæstelser.

Hvis du torturerer spionen vil han afsløre informationen om byen. Dette vil dræbe ham, men du vil redde tusinde civilister.

For du spionen til at prisgive informationen ved at torturere ham, så i kan stoppe angrebet på den nærliggende by?

**48) Impersonal-Instrumental**

Du officer i hæren i krig. Dine soldater har fundet en vigtig spion som sidder fast i en kollapseret bygning og desuden er alvorligt såret. Over radioen er du blevet informeret om at fjendens soldater planlægger et overraskelsesangreb på en nærliggende by, men du ved ikke om hvilken by det drejer sig. Angrebet vil dræbe tusinde civilister hvis du og dine soldater ikke når at stoppe det. Spionen ved, hvilken by det er. Du har forhørt ham men han vil ikke fortælle dig det. Han vil snart dø af sine kvæstelser. Rotter er begyndt at gnave i ham, og han lider meget.

Hvis du lader rotterne blive ved med denne tortur, vil spionen prisgive informationen om byen. Dette vil dræbe ham, men du vil redde tusinde civilister.

For du spionen til at prisgive informationen ved at lade rotterne gnave vidre i ham, så i kan stoppe angrebet på den nærliggende by?

**49) Personal – Others Beneficial – Inevitable (instrumental)**

Du er læge. En af dine patienter, som er HIV positiv, er ved at modtage en blodtransfusion før han skal udskrives af hospitalet, da han fortæller dig, at han agter at inficere så mange mennesker som muligt, efter at have forladt hospitalet. Du er bundet af din tavshedspligt og har ingen legale muligheder for at stoppe ham, fordi politiet ikke kan anholde ham baseret på beskyttede oplysninger.

Hvis du forurener hans medicin med en gift der ikke kan spores, vil du undgå at han kan inficere andre. Han vil dø af det, men du vil redde mange andre.

Forhindrer du at HIV bliver spredt ved at forurene din patients medicin for at forhindre at han smitter mange andre?

**50) Impersonal – Others Beneficial – Inevitable (instrumental)**

Du er læge. En af dine patienter, som er HIV positiv, er ved at modtage en blodtransfusion før han skal udskrives af hospitalet, da han fortæller dig, at han agter at inficere så mange mennesker som muligt, efter at have forladt hospitalet. Du er bundet af din tavshedspligt og har ingen legale muligheder for at stoppe ham, fordi politiet ikke kan anholde ham baseret på beskyttede oplysninger.

Hvis du ændrer hans blodgruppe i hospitalets database vil han få det forkerte blod og du vil undgå at han kan smitte andre. Han vil dø af det, men du vil redde mange andre.

Forhindrer du at HIV bliver spredt ved ændre din patients blodtype i databasen for at forhindre at han smitter mange andre?

**51) Personal – Others Beneficial – Avoidable (instrumental)**

Du er ved at forhandle med en sindsforvirret og bevæbnet mand på taget af en bygning. Nedenfor er der en menneskemængde på byens kunst og musikfestival. Han vil skyde ind i mængden, og selvom du har prøvet kan du ikke overbevise ham om ikke at gøre det. Mængden aner intet om truslen og du har ingen mulighed for at advare dem. Fra hans position kan han dræbe en halv snes mennesker, uden at tælle dem der ville blive såret eller dræbt under det opstående kaos.

Hvis du løber hurtigt hen til ham og skubber ham ned af taget, undgår du at han skydder ind i mængden. Dette til dræbe ham men du vil redde mange liv.

Undgår du kaoset ved at skubbe denne mand ned fra taget ned så han ikke skydder ind i mængden?

**52) Impersonal – Others Beneficial – Avoidable (instrumental)**

Du er ved at forhandle med en sindsforvirret og bevæbnet mand på taget af en bygning. Nedenfor er der en menneskemængde på byens kunst og musikfestival. Han vil skyde ind i mængden, og selvom du har prøvet kan du ikke overbevise ham om ikke at gøre det. Mængden aner intet om truslen og du har ingen mulighed for at advare dem. Fra hans position kan han dræbe en halv snes mennesker, uden at tælle dem der ville blive såret eller dræbt under det opstående kaos.

Hvis du advarer politiet om at forhandlingen er mislykket, vil de skyde manden, og du undgår at han skydder ind i mængden. Dette til dræbe ham, men du vil redde mange liv.

Undgår du kaoset ved at advare politet, der vil skydde ham, så han ikke skydder ind i mængden?
