# Supplementary material for: Moral judgment reloaded: a moral dilemma validation study
Source: Front Psychol. 2014 Jul 1;5:607. doi: 10.3389/fpsyg.2014.00607 (PMC4077230; doi:10.3389/fpsyg.2014.00607)
Supplement: Supplementary file 7 [file DataSheet7.DOC]

**English**

**DILEMMAS**

**1) Personal - Instrumental**

You and five other people are trapped in a burning building. There is only one emergency exit through which all of you could escape, but it is blocked by burning debris. Another injured person is about to crawl through a hole at the bottom of the exit door. You and the five people behind you do not have time to do the same.

If you use the injured person to break down the debris you will be able to escape. Doing this will certainly kill him, but it will save you and the five people behind you.

Do you break down the blockage by using the injured person so you and the five other people can escape?

**2) Impersonal- Accidental**

You and five other people are trapped in a burning building. There is only one emergency exit through which all of you could escape, but it is blocked by burning debris. Another injured person is about to crawl through a hole at the bottom of the exit door. You and the five people behind you do not have time to do the same.

If you activate the emergency system it will eliminate oxygen from the hallway which will put out the fire. However, it will leave the injured person without air. This will kill him, but you and the five people behind you will be saved.

Do you put out the fire by activating the emergency system, which will leave the injured without air, so you and the five other people can escape?

**3) Personal – accidental**

Enemy soldiers have taken over your village and will kill all civilians above the age of two. You and ten neighbors are hiding in two rooms of the cellar of a large house. You can hear the voices of soldiers who have come to search the house for valuables. Your baby begins to cry loudly. The crying will attract the attention of the soldiers, who will spare your baby’s life, but will kill you and the other refugees in both rooms.

If you put your hand over its mouth the crying will be absorbed, but your baby will not be able to breathe. This will kill it, but it will save you and the other ten neighbors.

Do you absorb the crying by putting your hand over your baby’s mouth, which will leave it without air, so the soldiers won’t find you and the ten neighbors?

**4) Impersonal- accidental**

Enemy soldiers have taken over your village and will kill all civilians above the age of two. You and ten neighbors are hiding in two rooms of the cellar of a large house. You can hear the voices of soldiers who have come to search the house for valuables. Your baby begins to cry loudly. The crying will attract the attention of the soldiers, who will spare your baby’s life, but will kill you and the other refugees in both rooms.

If you activate a noisy boiler it will cushion the crying, but it will become uncomfortably hot. The heat will be mortal for your baby, but it will save you and the ten neighbors.

Do you cushion the crying by activating the noisy boiler which will asphyxiate the baby, so they won’t find you and the ten neighbors?

**5) Personal – accidental**

You are a crewmember on a submarine traveling under a large iceberg. An explosion has damaged the ship, injured several crewmembers and collapsed the only access between the upper and lower decks of the ship. You and ten survivors are in the upper section, which does not have enough oxygen for all of you to survive until you reach the surface. One single crewmember is lying unconscious in the lower section, where there is enough oxygen.

If you push the emergency access hatch between the upper and lower sections of the ship it will allow oxygen to flow into the upper section. However, the hatch will fall down on the crewmember in the lower section and kill him, but it will save you and the ten survivors.

Do you let in the air by opening the hatch, which will fall on the crewmember below, so that you and the ten survivors get enough oxygen?

**6) Impersonal- accidental**

You are a crewmember on a submarine traveling under a large iceberg. An explosion has damaged the ship, injured several crewmembers and collapsed the only access between the upper and lower parts of the ship. You and ten survivors are in the upper section, which does not have enough oxygen for all of you to survive until you reach the surface. One single crewmember is lying unconscious in the lower section, where there is enough oxygen.

If you push an emergency button a hatch between the upper and lower sections of the ship will be released and will allow the oxygen to flow into the upper section. However, the hatch will fall on the crewmember in the lower section and kill him, but it will save you and the ten survivors.

Do you push the button, which will make the hatch fall down on the crewmember, so that you and the ten survivors get enough oxygen?

**7) Personal** – **instrumental**

You and ten divers are part of an U.N. team who is deactivating anti-ship mines from World War II. One team member has hurt himself and the blood in the water has attracted several sharks. You have an underwater rifle but only one harpoon and there are many sharks. The bleeding diver is swimming towards the last protective cage and will reach it before you and the others. The sharks, following the blood, are coming too close for you and the other divers to escape.

If you shoot at the injured diver this will kill him and the sharks will stop to eat him, but you and the ten divers will be saved.

Do you let the sharks eat the injured diver by shooting at him, so you and the other ten divers can reach the protective cage?

**8) Impersonal-Instrumental**

You and ten divers are part of an U.N. team who is deactivating anti-ship mines from World War II. One team member has hurt himself and the blood in the water has attracted several sharks. You have an underwater rifle, but only one harpoon and there are many sharks. The bleeding diver is swimming towards the last protective cage and will reach it before you and the others. The sharks, following the blood, are coming too close for you and the other divers to escape.

If you detonate one of the mines by mean of a remote control, it will kill the injured diver and the sharks will stop to eat him. Then, you and the ten divers will be saved.

Do you let the sharks eat the injured diver by detonating the mine next to him with a remote control, so you and the other ten divers can reach the protective cage?

**9) Personal – instrumental**

You work in an orphanage in a war swept country that hosts ten orphans. Rebel soldiers have taken over the government and are now killing everyone who does not support their regime. Moreover, they are conducting an ‘ethnic cleansing’ genocide campaign to kill everyone of a particular ethnic group. You and the eleven children of the orphanage did not support the rebellion and you also have one child of this ethnic group in your orphanage. There is no way for you and the other children to escape and the soldiers will certainly kill you all.

If you smother the child of the pursued ethnic group to death with a pillow while he is sleeping, the soldiers will believe that you support their regime. This will kill the child, but it will save you and the remaining ten children.

Do you make the soldiers believe you are supporting their regime by smothering that one child, so they will spare your and the other ten children’s life?

**10) Impersonal – Instrumental**

You work in an orphanage in a war swept country that hosts ten orphans. Rebel soldiers have taken over the government and are now killing everyone who does not support their regime. Moreover, they are conducting an ‘ethnic cleansing’ genocide campaign to kill everyone of a particular ethnic group. You and the eleven children of the orphanage did not support the rebellion and you also have one child of this ethnic group in your orphanage. There is no way for you and the other children to escape and the soldiers will certainly kill you all.

If you order a nurse to give the child of the pursued ethnic group a lethal overdose of sleeping pills, the soldiers will believe that you support their regime. This will kill the child, but it will save you and the remaining ten children.

Do you make the soldiers believe you are supporting their regime ordering the nurse to give that one child an overdose, so they will spare your and the other ten children’s life?

**11) Personal – instrumental**

You are a Peace Corps health-worker who is volunteering in a rural African village. A man from a nearby village has been infected with the Ebola virus that is extremely contagious, incurable, and almost always deadly. Unexpectedly, this man is still alive which means he must be immune to the virus’ deadly consequences. However, he wrongly believes that your health center can cure his disease completely. You see him approaching and you know that if he enters the village he will pass the virus to 119 innocent people who, unlike him, will die.

If you shoot him with the gun of the health center you will prevent him from spreading the virus to you and the rest of the village. This will kill him, but it will save you and the 119 inhabitants of the village.

Do you prevent the man from entering the village by shooting him, so he won’t spread the virus to you and the 119 people of the village?

**12) Impersonal-Instrumental**

You are a Peace Corps health-worker who is volunteering in a rural African village. A man from a nearby village has been infected with the Ebola virus that is extremely contagious, incurable, and almost always deadly. Unexpectedly, this man is still alive, which means he must be immune to the virus’ deadly consequences. However, he wrongly believes that your health center can cure his disease completely. You see him approaching and you know that if he enters the village he will pass the virus to 119 innocent people who, unlike him, will die.

If you warn the village council they will shoot him to prevent him from spreading the virus to you and the rest of the village. This will kill him, but it will save you and the 119 inhabitants of the village.

Do you prevent the man from entering into the village by warning the village council so that they shoot him, so he won’t spread the virus to you and the 119 people of the village?

**13) Personal – instrumental**

You are a doctor alone in a rescue helicopter during a devastating flood. You have rescued eight critically injured people and you are keeping them alive. Suddenly, the pilot notifies you of a problem in the engine and gives the order that the helicopter needs to release weight or it will crash and you will all die. You cannot abandon the helicopter because the wounded need your help and the heavy medical equipment is bolted down.

If you throw off one of the injured people, you will release enough weight to keep the helicopter in the air. This will kill him, but it will save you and the eight people on board.

Do you release weight by throwing one of the injured people off the helicopter, so you and the eight persons on board will be able to stay in the air?

**14) Impersonal-Instrumental**

You are a doctor alone in a rescue helicopter during a devastating flood. You have rescued eight critically injured people and you are keeping them alive. Suddenly, the pilot notifies you of a problem in the engine and gives the order that the helicopter needs to release weight or it will crash and you will all die. You cannot abandon the helicopter because the wounded need your help and the heavy medical equipment is bolted down.

If you cut the cable by which the last rescued person is ascending, you will release enough weight to keep the helicopter in the air. This will kill him, but it will save you and the eight people on board.

Do you release weight by cutting the cable by which the last person is ascending, in order to keep you and the eight people on board in the air?

**15) Personal – accidental**

You are an engineer on the international space station ISS. Suddenly, a fire breaks out in the cargo bay. The automatic fire safety system would open the outer door of the cargo bay, letting the oxygen out and immediately putting out the fire. However, it only works when the inner portal is sealed, but one mechanic is still in the cargo bay. You realize that he doesn’t have the time to take off the bulky space suit and that he’ll get stuck when trying to get through the inner portal, causing the fire to spread and to kill you all.

If you push the mechanic back into the cargo bay, the portal will close activating the emergency system which will put out the fire. This will suck the mechanic into space and kill him, but it will save you and the other ten astronauts.

Do you put out the fire by manually sealing the inner portal, which will suck the mechanic into space, so the fire won’t reach you and the ten astronauts?

**16) Impersonal – accidental**

You are an engineer on the international space station ISS. Suddenly, a fire breaks out in the cargo bay. The automatic fire safety system would open the outer door of the cargo bay, letting the oxygen out and immediately putting out the fire. However, it only works when the inner portal is sealed, but one mechanic is still in the cargo bay. You realize that he doesn’t have the time to take off the bulky space suit and that he’ll get stuck when trying to get through the inner portal, causing the fire to spread and to kill you all.

If you push a button before the mechanic reaches the inner portal, the portal will close activating the emergency system which will put out the fire. This will suck the mechanic into space and kill him, but it will save you and the remaining ten astronauts.

Do you put out the fire pushing the button which seals the inner portal, sucking the mechanic into space, so the fire won’t reach you and the ten astronauts?

**17) Personal – instrumental**

You are an inspector of a nuclear power plant. The plant foreman and you are visiting the facility when one of the supply conduits overheats. The emergency coolant system fails to activate, and a chain reaction which will result in a nuclear meltdown is about to start. This will release lethal radiation into the nearby town, killing thousands of people.

If you push the foreman onto the conduits it will release enough heat to prevent the nuclear chain reaction. This will kill him, but it will save you and the thousands of town inhabitants.

Do you cool down the conduits by pushing the foreman down on them, so the nuclear chain reaction will be stopped in time for you and the thousands of people?

**18) Impersonal – accidental**

You are an inspector of a nuclear power plant. The plant foreman and you are visiting the facility when one of the supply conduits overheats. The emergency coolant system fails to activate, and a chain reaction which will result in a nuclear meltdown is about to start. This will release lethal radiation into the nearby town, killing thousands of people.

If you insert liquid nitrogen manually into the supply chamber it will release enough heat to prevent the nuclear chain reaction. This will kill one employee, who is trapped in the supply chamber, but it will save you and the thousands of town inhabitants.

Do you release the heat inserting liquid nitrogen into the supply chamber where the employee is trapped, so the nuclear chain reaction will be stopped in time for you and the thousands of people?

**19) Personal – instrumental**

You are the explosives expert of a company that has been hired to demolish a skyscraper. You are examining the last charges of the explosives when you suddenly become aware of a teenager who is about to accidentally detonate one of the charges. The explosion will result in the building’s uncontrolled collapse over you, the teenager, and the crowd of spectators. The teenager stands several floors below you and cannot hear you.

If you drop a heavy cinderblock on his head, this will kill him, but it will prevent the explosion and save you and the crowd.

Do you stop the teenager by dropping the cinderblock on his head, so the building won’t collapse over you and the crowd of spectators?

**20) Impersonal-Instrumental**

You are the explosives expert of a company that has been hired to demolish a skyscraper. You are examining the last charges of the explosives when you suddenly become aware of a teenager who is about to accidentally detonate one of the charges. The explosion will result in the building’s uncontrolled collapse over you, the teenager, and the crowd of spectators. The teenager stands several floors below you and cannot hear you.

If you reactivate the building’s electricity, the boy will get an electric shock because he is touching an open circuit. This will kill him, but it will save you and the crowd by preventing the explosion.

Do you stop the teenager by reactivating the building’s electricity, so the building won’t collapse over you and the crowd of spectators?

21) **Personal – instrumental**

You are a construction worker. You and your crew are standing on some scaffolding, working on a skyscraper. Suddenly, the scaffolding collapses partially. You and several others are hanging on to a dangling crossbar, but it cannot hold the weight of all of you. A worker next to you slips off the crossbar and grabs your one free arm. However, you realize that the entire structure is about to give way.

If you repeatedly kick your coworker in the stomach and chest until he lets go of your hand and falls, you will remove just enough weight so the rest of you can make it to safety before the scaffolding collapses entirely. This will kill your colleague but it will safe you and the others.

Do you remove weight by hitting this colleague until he lets go of your arm and falls, so you and the others can continue to hold on to the structure?

**22) Impersonal-Instrumental**

You are a construction worker. You and your crew are standing on some scaffolding, working on a skyscraper. Suddenly, the scaffolding collapses partially. You and several others are hanging on to a dangling crossbar, but it cannot hold the weight of all of you. The worker next to you slips off the crossbar and catches himself on another portion of the scaffolding. The entire structure is about to give way.

If you pull out a latch that will detach the section of scaffolding with your co-worker on it, this will remove just enough weight that the rest of you can make it to safety before the scaffolding collapses entirely. This will kill him but save you and the other five coworkers.

Do you remove weight by pulling out the latch so your co-worker falls off the crossbar, so you and the others can continue to hold on to the structure?

**23) Personal – accidental**

You are the bus driver of a kindergarten trip to the zoo. On the way, faulty tires cause the bus to plunge off a bridge and into a roaring river. You and three of the children are still in the sinking bus. The rest has already been swept away by the river. You grab the two children next to you and begin to swim towards the exit. The third child latches onto your leg. You feel that you are not strong enough to fight against the current and swim with all three children.

If you shake off the third child you will be able to fight against the current, but the child dropped behind will drown. This will kill him, but it will save you and the other two children.

Do you fight against the current shaking off the child, which will make him to drown, so you and the other two children can reach the surface?

**24) Impersonal-accidental**

You are the bus driver of a kindergarten trip to the zoo. On the way, faulty tires cause the bus to plunge off a bridge and into a roaring river. You and three of the children are still in the sinking bus. The rest has already been swept away by the river. You grab the two children next to you and begin to swim towards the exit, but bulky seats are blocking it.

If you pull the seats away you will free the exit, but you will block the corridor and the third child will be trapped, causing him to drown. This will kill him, but it will save you and the other two children.

Do you free the exit putting the seats in the corridor, which will trap the third child, so you and the other two children can reach the surface?

**(25) Personal – instrumental**

You are a doctor. You have five patients who will all die if they don’t get an immediate organ transplant. Another patient is just being rushed to the hospital after a serious car accident. This person is critically injured, but you can probably save him by means of a long and complicated operation.

If you secretly cut his carotid artery during surgery it will cause his death and you will have the organs for the other five patients. This will kill him, but save the other five patients.

Do you obtain the organs cutting the carotid artery of the accident victim, so you can undertake the transplantations for the other five patients?

**26) Impersonal-Instrumental**

You are a doctor. You have five patients who will all die if they don’t get an immediate organ transplant. Another patient is just being rushed to the hospital after a serious car accident. This person is critically injured. During the long and complicated surgery you notice that a nurse is about to inject the wrong medication, which will be lethal in this case.

If you let the nurse inject the wrong medication the accident victim will die and you will have the organs for the other five patients. This will kill this patient, but will save the other five patients.

Do you obtain the organs letting the nurse inject the wrong medication, so you can undertake the transplantations for the other five patients?

**27) Personal-27) Impersonal – accidental**

You are part of a shipyard dock team that attaches crane cables to containers to unload the cargo ships. You and the others have just attached cables to a container and are now climbing on top of it to make sure it is unloaded properly. Suddenly the red warning light flashes indicating that the crane cable is about to fail. You see that the reason for this is a fight between two crewmembers. The container is swinging dangerously over five other crewmembers on the deck.

If you push the two fighting crewmembers to separate them, you will avoid having the container fall down. However, one of the crewmembers is not wearing his safety harness and will fall off the container. This will kill him, but it will save the five crewmembers on the deck.

Do you stop the swinging of the container by pushing your colleagues, which will cause the one without safety harness to fall down, so the container won’t collapse onto the five crewmembers on the deck?

**28) Impersonal – accidental**

You are part of a shipyard dock team that attaches crane cables to containers to unload the cargo ships. You and the others have just attached cables to a container and are now climbing on top of it to make sure it is unloaded properly. Suddenly the red warning light flashes indicating that a cable is about to fail. If it fails over the deck the container will collapse onto five crewmembers.

If you push the emergency release button the container will be dropped back into the cargo bay. You and the others will be held suspended in mid air by your safety harnesses, but one crewmember is still working in the cargo bay. Dropping the container back there will kill him, but it will save the five crewmembers on the deck.

Do you drop the container pushing the emergency button, which will cause the container to fall back into the cargo bay on the crewmember, so the container won’t collapse onto the five crewmembers on the deck?

**29) Personal – accidental**

A viral epidemic has spread across the globe killing thousands of people. You are a medical researcher and have developed two substances in your laboratory. Due to a mistake both are labeled as vaccine. One of them is indeed the vaccine, which could save thousands of lives. The other one is lethal.

If you inject the substances to your two lab assistants without knowing which substance is which, you will be able to identify the vaccine. One of the assistants will die, but you will be able to save thousands of lives with your vaccine.

Do you identify the vaccine injecting these two substances into your two lab assistants which will poison one of them, so thousands of people can be vaccinated?

**30) Impersonal-accidental**

A viral epidemic has spread across the globe killing thousands of people. You are a medical researcher and have developed two substances in your laboratory. Due to a mistake both are labeled as vaccine. One of them is indeed the vaccine, which could save thousands of lives. The other one is lethal.

If you allow a nurse to inject the two substances to two patients, you will be able to identify the vaccine. One of the patients will die, but you will be able to save thousands of lives with the vaccine.

Do you identify the vaccine allowing the nurse to inject the two substances to two patients which will poison one of them, so thousands of people can be vaccinated?

**31) Personal – instrumental**

A runaway trolley is speeding down the tracks towards five workmen who will be killed if the trolley continues on its present course. You are standing next to the tracks, but you are too far away to warn them. Next to you there is a very large stranger.

If you push the large stranger onto the tracks, the trolley will slide off the tracks and won’t continue its course towards the workmen. This will kill the stranger, but you will save the five workmen.

Do you cause the trolley to derail pushing the stranger onto the tracks, so the trolley does not reach the five workmen?

**32) Impersonal – accidental**

A runaway trolley is speeding down the tracks towards five workmen who will be killed if the trolley continues on its present course. You are standing next to the tracks, but you are too far away to warn them. Next to you there is a control switch that can redirect the trolley onto a different track, where only one workman is working.

If you flip the control switch it will divert the trolley onto the track with one workman and will not continue its course towards the five workmen. This will kill this workman, but it will save the other five.

Do you divert the trolley by flipping the control switch, which will cause the trolley to run over one workman, so the trolley does not reach the five workmen?

**33) Personal – instrumental**

You and a fellow researcher have discovered a powerful new energy source that is cheap, safe, and clean. It has the potential to put an end to pollution and poverty in the world However, your colleague wants to sell the discovery and you know that he is planning to contact the potential buyers today. You also know for sure that the latter will use the invention as a deadly weapon, but your colleague doesn’t believe you.

If you poison your colleague with a common poison available in your lab, you will avoid having the discovery fall into the wrong hands. The poison will kill your colleague, but you will save thousands of people.

Do you prevent the sale of the discovery by poisoning your colleague, so the source of energy can’t be used as a weapon against thousands of people?

**34) Impersonal-Instrumental**

You and a fellow researcher have discovered a powerful new energy source that is cheap, safe, and clean. It has the potential to put an end to pollution and poverty in the world However, your colleague wants to sell the discovery and you know that he is planning to contact the potential buyers by email today. You also know for sure that the latter will use the invention as a deadly weapon, but your colleague doesn’t believe you.

If you release a flammable gas in the lab, it will cause an explosion when your colleague turns on his computer. This will kill him, but you will save thousands of people.

Do you cause the computer of your colleague to explode releasing the flammable gas, in order to prevent the discovery being used as a weapon against thousands of people?

**35) Personal – instrumental**

You are an expert motorcyclist participating in a Bike Week. As you are driving down the road in front of a large group of ten bikers, you suddenly notice that a biker up front is losing control over his machine. As you speed up to pull alongside him, you realize that he is going to crash any moment. This would result in a large pile-up and the group of ten bikers behind you will die.

If you force this biker off the road he will crash into the trees, but you will prevent the pile-up. At your current speed this will kill him, but you will save the group of ten riders.

Do you prevent the large pile-up by forcing this biker off the road, in order to impede that the group of ten bikers crash into him?

**36) Impersonal-instrumental**

You are an expert motorcyclist participating in a Bike Week. As you are driving down the road in front of a large group of ten bikers, you suddenly notice that a biker up front is losing control over his machine and is falling. You can easily evade him yourself but the bikers behind you will crash into each other and die in the resulting pile-up.

If you let your bike run into the falling biker while jumping into the roadside ditch yourself, you will warn the others in time. This will kill this biker, but you will save the other ten.

Do you alert the others about the imminent accident by running your bike into the falling bikers’ machine, so the other ten riders will have enough time to drive around him?

**37) Personal – instrumental**

You are the leader of a small group of soldiers. You are on your way back from a mission in enemy territory when one of your men steps in a trap that catches his leg, injuring him badly. You cannot free him without killing him, but if you leave him behind, enemy troops will torture him until he reveals the position of an important allied base camp. After that the soldiers will attack this camp and kill the 10 soldiers of the platoon there. The soldier begs you not to leave him behind, but the enemy troops are approaching and it is dangerous for you and your men to stay because you don’t have any more ammunition.

If you stab the soldier yourself you will impede that he’ll be tortured and reveal the information which leads to the death of the allied platoon. This will kill him, but you will save the ten soldiers of the platoon.

Do you impede that the trapped soldier will be tortured by stabbing him to death, so he can’t reveal the location of the ten allied troop members?

**38) Impersonal – Instrumental**

You are the leader of a small group of soldiers. You are on your way back from a mission in enemy territory when one of your men steps in a trap that catches his leg, injuring him badly. You cannot free him without killing him, but if you leave him behind, enemy troops will torture him until he reveals the position of an important allied base camp. After that the soldiers will attack this camp and kill the 10 soldiers of the platoon there. The soldier begs you not to leave him behind, but the enemy troops are approaching and it is dangerous for you and your men to stay because you don’t have more ammunition.

If you signalize the position of the trapped soldier with an illumination rocket, the region will be bombed. Thus, you will avoid that the soldier is tortured and reveals the information which will lead to the death of the allied troop. This will kill him, but you will save the ten soldiers of the allied platoon.

Do you impede that the trapped soldier is tortured by ordering the bombing of that region, so he can’t reveal the location of the ten allied troop members?

**39) Personal – instrumental**

You are on the night watch in a hospital. A chemical product has been spilled in a room with six patients. This chemical is highly toxic because once exposed to oxygen it evaporates and transforms into a poisonous gas which will kill all the patients in the room. You cannot wipe it away with conventional towels or wiping clothes. However, human skin absorbs it swiftly.

If you place one of the patients on the chemical you will prevent it from evaporating. This will kill this patient but you will save the other five patients in the room.

Do you prevent the toxic evaporation placing one of the patients on the chemical, so the other five patients won’t be poisoned?

**40) Impersonal – accidental**

You are on the night watch in a hospital. A chemical product has been spilled in a room with five patients. This chemical is highly toxic because once exposed to oxygen it evaporates and transforms into a poisonous gas which will kill all the patients in the room. You cannot wipe it away with conventional towels or wiping clothes.

If you activate the ventilation system the poisonous gas will be taken out of the room. However, it will be transferred into a room upstairs with one patient, whom you won’t be able to evacuate in time. This will kill this patient, but you will save the other five.

Do you divert the gas out of the room by activating the ventilation system, causing it to be transferred to the room with one patient, so the other five patients won’t be poisoned?

**41) Personal – instrumental**

You are on a rowboat with a tour guide on a lake in Alaska. Nearby, three children have fallen off their boat and are in danger of freezing to death. You rescue the children, but one of them is very heavy and you realize that if you keep him on board your boat will sink and all will die. Neither you nor the guide can leave the boat because you are rowing and the guide is giving CPR to one of the children.

If you throw the heavy child off the boat you will be able to reach the shore. This will kill this child, but you will save the other two.

Do you get rid of load by throwing that one child overboard, so you can reach the shore with the other two children?

**42) Impersonal- accidental**

You are on a rowboat with a tour guide on a lake in Alaska. Nearby, three children have fallen off their boat and are in danger of freezing to death. After you rescue two of the children, you realize that the weight of the third child would cause your boat to sink and all would die. Neither you nor the guide can leave the boat because you are rowing and the guide is giving CPR to one of the children.

If you row to the shore leaving the third child behind in the water, you will prevent your boat from sinking. This will kill the child left behind, but you will save the other two.

Do you prevent your boot from sinking by leaving the third child behind, which will cause him to die, so that you can reach the shore with the other two children?

**43) Personal – instrumental**

You are a miner. The only way out of the mine is to ride up with a simple cable elevator with small cubicles. It is the end of your shift and everybody is riding up to the surface in the cubicles. While you are in the queue, you see that the cable supporting all the cubicles is about to snap. 10 miners are riding in the cubicles at this time.

If you knock over the last cubicle, the miner inside will fall down. In this way, you will reduce the load enough for the cable to resist breaking, and bring the other 10 miners to the exit. This will kill the miner in the cubicle you knock over, but you will save the other 10.

Do you reduce weight by knocking over the cubicle with only one miner, so that the other ten miners will reach the exit?

**44) Impersonal- instrumental**

You are a miner. The only way out of the mine is to ride up with a simple cable elevator with small cubicles. It is the end of your turn and everybody is riding up to the surface in the cubicles. While you are in the queue, you see that the cable supporting all the cubicles is about to snap. 10 miners are riding the cubicles at this time.

If you push an emergency switch the last cubicle will be detached from the cable. This will reduce enough weight to maintain the cubicles of the other 10 miners. This will kill the miner in the detached cubicle, but it will save the other ten.

Do you reduce weight by pushing the emergency switch which detaches the last cubicle, so that the other ten miners can reach the exit?

**45) Personal – accidental**

You are an electrician hired to fix an electrical problem in the home of a very wealthy tycoon who is terminally ill of cancer. Recently, it has been announced that upon his death, his enormous fortune will be donated to the local children’s hospital. You know that there are 109 children at this hospital who could be saved with this money, but who cannot wait much longer before they die.

If you suffocate the tycoon while he is sleeping by covering his nose and mouth with your gloved hands, this essential donation for the children will arrive in time. This will kill the tycoon, but you will save the lives of 109 children.

Do you speed the donation by suffocating this man, so that the donation will get to the 109 ill children in time?

**46) Impersonal-Instrumental**

You are an electrician hired to fix an electrical problem in the home of a very wealthy tycoon who is terminally ill of cancer. Recently, it has been announced that upon his death, his enormous fortune will be donated to the local children’s hospital. You know that there are 112 children at this hospital who could be saved with this money, but who cannot wait much longer before they die.

If you short-circuit the cables instead of fixing the problem, the tycoon’s life support equipment will fail and this essential donation for the children will arrive in time. This will kill the tycoon, but you will save the lives of 112 children.

Do you anticipate the donation by causing the tycoon’s life support equipment to fail, so that the donation gets to the 112 ill children in time?

47) **Personal – instrumental**

You are a military officer during a war. Your soldiers have found an important enemy spy trapped and injured in a collapsed building. Over the radio you have been informed that enemy soldiers are planning a surprise attack on a nearby town, but you don’t know which. They will kill thousands of civilians if you and your soldiers do not stop them. The spy knows which town will be attacked. You have questioned him but he doesn’t want to tell you. He won’t survive much longer due to his injuries.

If you torture the spy, he will reveal the information about the town. The torture will kill him, but you will save thousands of civilians.

Do you make the spy reveal the information by torturing him, so you can prevent the attack on thousands of civilians?

**48) Impersonal-Instrumental**

You are a military officer during a war. Your soldiers have found an important enemy spy trapped and injured in a collapsed building. Over the radio you have been informed that enemy soldiers are planning a surprise attack on a nearby town, but you don’t know which. They will kill thousands of civilians if you and your soldiers do not stop them. The spy knows which town will be attacked. You have questioned him but he doesn’t want to tell you. He won’t survive much longer due to his injuries. Rats have begun to chew his legs and he is suffering a lot.

If you let the rats continue with this torture the spy will reveal the information about the town. The torture will kill him, but you will save thousands of civilians.

Do you make the spy reveal the information by letting the rats continue to torture him, so that you can prevent the attack on the thousands of civilians?

**49) Personal – Others Beneficial – Inevitable (instrumental)**

You are a doctor. One of your patients, who is HIV positive, is about to receive a blood transfusion before being released from the hospital. He has told you, in the confidence of your doctor-patient relationship, that he intends to infect as many people as possible starting that evening, after leaving the hospital. There is no legal way to stop him, because the police cannot arrest him based on protected information.

If you contaminate his medication by putting an untraceable poison in it, you will prevent him from infecting others. This will kill him, but you will save many.

Do you prevent the transmission of AIDS by poisoning the patient, so he won’t be able to infect other people?

**50) Impersonal – Others Beneficial – Inevitable (instrumental)**

You are a doctor. One of your patients, who is HIV positive, is about to receive a blood transfusion before being released from the hospital. He has told you, in the confidence of your doctor-patient relationship, that he intends to infect as many people as possible starting that evening, after leaving the hospital. There is no legal way to stop him, because the police cannot arrest him based on protected information.

If you change his blood-type in the hospital’s database he will receive the wrong blood and you will prevent him from infecting many people. This will kill him, but you will save many.

Do you prevent the transmission of AIDS by changing the blood-type of the patient, so he won’t be able to infect other people?

**51) Personal – Others Beneficial – Avoidable (instrumental)**

You are negotiating with a psychologically ill and armed man on the roof of a building. Down below there is a crowd of people at the city’s art and music festival. He wants to shoot people in the crowd and you have tried but cannot talk him out of it. The crowd does not realize the threat and you have no way to warn them. From where he is standing, he could easily kill a dozen people, not counting those who would be injured or killed by the resulting chaos.

If you run over and shove him off the roof of the building he won’t be able to shoot at people in the crowd. This will kill him, but you will save the lives of many.

Do you prevent the chaos by shoving the man off the roof, so he won’t shoot at people in the crowd?

**52) Impersonal – Others Beneficial – Avoidable (instrumental)**

You are negotiating with a psychologically ill and armed and man on the roof of a building. Down below there is a crowd of people at the city’s art and music festival. He wants to shoot people in the crowd and you have tried but cannot talk him out of it. The crowd does not realize the threat and you have no way to warn them. From where he is standing, he could easily kill a dozen of people, not counting those who would be injured or killed by the resulting chaos.

If you inform the police that the negotiation has failed, they will shoot him and prevent the shooting at the crowd. This will kill him, but you will save the lives of many.

Do you prevent the chaos by letting the police shoot him, so he won’t shoot at people in the crowd?
