# Supplementary material for: Moral judgment reloaded: a moral dilemma validation study
Source: Front Psychol. 2014 Jul 1;5:607. doi: 10.3389/fpsyg.2014.00607 (PMC4077230; doi:10.3389/fpsyg.2014.00607)
Supplement: Supplementary file 8 [file DataSheet8.DOC]

**French**

**DILEMMAS**

**1) Personal - Instrumental**

Vous et cinq autres personnes êtes piégés dans un immeuble en feu. Il n'y a qu'une seule sortie d'urgence par laquelle vous pouvez tous vous échapper, mais elle est bloquée par des débris ardents. Une personne blessée est en train de ramper par un trou au bas de la porte. Vous et les cinq autres personnes derrière vous n'avez pas le temps de faire la même chose.

Si vous utilisez la personne blessée pour écarter les débris ardents, vous pouvez vous échapper. Elle va mourir, mais cela va vous sauver, vous et les cinq personnes derrière vous.

Utilisez-vous l'homme blessé pour écarter les débris ardents afin que vous et les cinq autres personnes puissiez vous échapper?

2) Impersonal- Accidental

Vous et cinq autres personnes êtes piégés dans un immeuble en feu. Il n'y a qu'une seule sortie d'urgence par laquelle vous pouvez tous vous échapper, mais elle est bloquée par des débris ardents. Une personne blessée est en train de ramper par un trou au bas de la porte. Vous et les cinq autres personnes derrière vous n'avez pas le temps de faire la même chose.

Si vous activez le système d'urgence cela va éliminer l'oxygène du couloir et éteindre le feu. La personne blessée va mourir asphyxiée, mais si vous ne le faites pas, vous et les cinq personnes derrière vous allez mourir.

Activez-vous le système d'urgence pour éteindre le feu, ce qui va asphyxier la personne blessée, afin que vous et les cinq autres personnes puissiez vous échapper?

3) Personal – accidental

Des soldats ennemis ont pris votre village et ont reçu l’ordre de tuer tous les civils âgés de plus de deux ans. Vous et quelques voisins vous êtes refugiés dans deux pièces au sous-sol d'une grande maison. Vous entendez la voix des soldats qui sont venus pour chercher des objets de valeur. Votre bébé commence à pleurer très fort.

Si vous couvrez sa bouche avec votre main pour éviter que ses pleurs n’attirent l'attention des soldats, qui vont épargner la vie de votre bébé mais vont vous tuer vous et les autres voisins, cela va étouffer votre bébé. Si vous ne le faites pas, vous et les 10 autres allez mourir.

Couvrez-vous la bouche de votre bébé avec la main, ce qui va l’étouffer, afin que les soldats ne vous trouvent pas, vous et les 10 autres voisins?

4) Impersonal- accidental

Des soldats ennemis ont pris votre village et ont reçu l’ordre de tuer tous les civils âgés de plus de deux ans. Vous et quelques voisins vous êtes refugiés dans deux pièces au sous-sol d'une grande maison. Vous écoutez la voix des soldats qui sont venus pour chercher des objets de valeur. Votre bébé commence à pleurer très fort.

Si vous activez une chaudière bruyante cela amortira le bruit. Cependant, elle va causer une chaleur inconfortable pour les adultes et les enfants, mais mortel pour votre bébé. Mais si vous ne le faites pas, vous et les 10 autres mourrez.

Activez-vous la chaudière pour amortir le son, ce qui va étouffer votre bébé, afin que les soldats ne vous trouvent pas, vous et les 10 autres voisins?

5) Personal – accidental

Vous faites partie de l'équipe d'un sous-marin qui navigue sous un gros iceberg. Une explosion a causé une avarie au bateau, blessant plusieurs de vos collègues et le seul accès entre les parties supérieure et inférieure du sous-marin a fondu. Vous et 10 autres survivants êtes dans la section supérieure, qui n'a pas assez d'oxygène pour que tous puissent parvenir à la surface. Un collègue est inconscient dans la partie inférieure, où il y a suffisamment d'oxygène.

Si vous poussez la porte de secours entre les deux sections, l’air se diffusera. Cependant, la porte va tomber sur le collègue et cela va le tuer, mais cela vous sauvera, vous et les 10 autres survivants.

Poussez-vous la trappe, ce qui va écraser le collègue, afin que vous et les 10 autres survivants ayez suffisamment d’oxygène?

6) Impersonal- accidental

Vous faites partie de l'équipe d'un sous-marin qui navigue sous un gros iceberg. Une explosion a causé une avarie au bateau, blessant plusieurs de vos collègues et le seul accès entre les parties supérieure et inférieure du sous-marin a fondu. Vous et 10 autres survivants êtes dans la section supérieure, qui n'a pas assez d'oxygène pour que tous puissent parvenir à la surface. Un collègue est inconscient dans la partie inférieure, où il y a suffisamment d'oxygène.

Si vous appuyez sur un interrupteur d'urgence, une porte de secours sera ouverte entre les deux sections et l’air se diffusera. Cependant, la porte va tomber sur le collègue et cela va le tuer, mais cela va vous sauver, vous et les 10 autres survivants.

Appuyez-vous sur l’interrupteur d'urgence, ce qui fera tomber la trappe sur le collègue, afin que vous et les 10 autres survivants ayez suffisamment d’oxygène?

**7) Personal** – **instrumental**

Vous et 10 plongeurs faites partie d'une équipe des Nations Unies qui désactive des mines sous-marines de la Seconde Guerre mondiale. Un membre de l'équipe a été blessé et son sang a attiré plusieurs requins. Vous n’avez qu’un harpon comme arme sous-marine, mais il y a trop de requins. Le plongeur saigne, il est en train de nager vers la cage protectrice et il va l’atteindre avant vous et les autres. Les requins en suivant le sang s’approchent trop près pour que vous et les 10 autres puissiez vous échapper.

Si vous abattez le plongeur qui saigne, cela va le tuer et les requins s’arrêteront pour le manger, mais vous et les 10 plongeurs vous pourrez vous échapper.

Harponnez-vous le plongeur blessé pour que les requins s’arrêtent pour le manger, afin que vous et les 10 autres puissiez vous échapper?

**8) Impersonal-Instrumental**

Vous et 10 plongeurs faites partie d'une équipe des Nations Unies qui désactive des mines sous-marines de la Seconde Guerre mondiale. Un membre de l'équipe a été blessé et son sang a attiré plusieurs requins. Vous n’avez qu’un harpon comme arme sous-marine, mais il y a trop de requins. Le plongeur saigne, il est en train de nager vers la cage protectrice et il va l’atteindre avant vous et les autres. Les requins en suivant le sang s’approchent trop près pour que vous et les autres 10 puissiez vous échapper ?

Si vous faites exploser une mine avec la commande à distance cela va tuer le blessé, mais vous et les 10 plongeurs pourrez vous échapper.

Faites-vous exploser la mine avec la commande à distance, pour que les requins s’arrêtent pour manger le plongeur afin que vous et les 10 autres puissiez vous échapper?

**9) Personal – instrumental**

Vous travaillez dans un orphelinat dans un pays en guerre. Des soldats rebelles ont pris le gouvernement et sont en train de tuer tous ceux qui n’appuient pas leur régime. En plus, ils font une campagne génocide contre un groupe ethnique en particulier. Vous et les onze orphelins n’avez pas appuyé la rébellion et vous avez un enfant de ce groupe ethnique dans votre orphelinat. Il n’y a aucune chance que vous puissiez vous échapper et il est sûr que les soldats vont tous vous tuer.

Si vous étouffez cet enfant avec un oreiller quand il dort, il va mourir, mais les soldats vont croire que vous appuyez leur régime et épargneront votre vie et celle des 10 autres enfants.

Faites-vous croire aux rebelles que vous appuyez leur régime en étouffant cet enfant afin que les soldats vous épargnent, vous et les autres enfants?

**10) Impersonal – Instrumental**

Vous travaillez dans un orphelinat dans un pays en guerre. Des soldats rebelles ont pris le gouvernement et sont en train de tuer tous ceux qui n’appuient pas leur régime. En plus, ils font une campagne génocide contre un groupe ethnique en particulier. Vous et les onze orphelins n’avez pas appuyé la rébellion et vous avez un enfant de ce groupe ethnique dans votre orphelinat. Il n’y a aucune chance que vous puissiez vous échapper et il est sûr que les soldats vont tous vous tuer.

Si vous ordonnez à une infirmière de donner une surdose mortelle de somnifères à l’enfant de ce groupe ethnique, les soldats vont croire que vous appuyez leur régime et épargneront votre vie et celle des 10 autres enfants.

Faites-vous croire aux rebelles que vous appuyez leur régime en ordonnant à l’infirmière de donner une surdose à cet enfant afin que les soldats épargnent votre vie et celle des autres enfants?

**11) Personal – instrumental**

Vous êtes collaborateur bénévole dans un village rural d'Afrique. Un homme d'un village voisin a été infecté par le virus de l’Ébola, qui est extrêmement contagieux, incurable et presque toujours mortel. Miraculeusement, il a survécu un mois, ce que veut dire qu’il est immunisé contre les effets mortels du virus. Toutefois, il croit à tort que votre centre de santé peut le guérir complètement. Vous le voyez s'approcher et vous savez que s’il entre dans le village il va contaminer 119 autres personnes innocentes qui, contrairement à lui, vont mourir.

Si vous l’abattez avec le pistolet du centre, vous allez le tuer, mais ainsi vous empêchez qu’il ne vous contamine, vous et le reste du village.

Empêchez-vous l’homme d’entrer dans le village en l’abattant afin qu’il ne vous contamine pas, vous et les 119 personnes du village?

**12) Impersonal-Instrumental**

Vous êtes collaborateur bénévole dans un village rural d'Afrique. Un homme d'un village voisin a été infecté par le virus de l’Ébola, qui est extrêmement contagieux, incurable et presque toujours mortel. Miraculeusement, il a survécu un mois, ce que veux dire qu’il est immunisé contre les effets mortels du virus. Toutefois, il croit à tort que votre centre de santé peut le guérir complètement. Vous le voyez s'approcher et vous savez que s’il entre dans le village il va contaminer 119 autres personnes innocentes qui, contrairement à lui, vont mourir.

Si vous avertissez le conseil municipal, ils vont l’abattre. Cela va le tuer, mais c’est la seule manière d’empêcher qu’il diffuse le virus à vous et aux gens du village.

Empêchez-vous l’homme d’entrer dans le village en avertissant le conseil municipal afin d’éviter qu’il ne vous contamine, vous et les 119 personnes du village?

**13) Personal – instrumental**

Vous êtes la/e seul/e médecin dans un hélicoptère de secours lors d’une inondation dévastatrice. Vous avez sauvé huit personnes gravement blessées et vous les maintenez en vie. Tout d’un coup, le pilote vous avertit d’une panne de moteur et ordonne de lâcher du lest sinon l’hélicoptère va s’écraser et vous allez tous mourir. Quant à vous, vous ne pouvez pas abandonner l’hélicoptère parce que les blessés ont besoin de vos soins et l’équipement médical lourd est fixé au sol.

Si vous jetez un des blessés dans le vide, cela va permettre de maintenir l'hélicoptère en l'air. Cela va tuer le blessé, mais vous allez être sauvés vous et les huit autres blessés.

Allégez-vous le poids en jetant un des blessés dans le vide afin de maintenir l'hélicoptère en l'air avec vous et les huit autres blessés?

**14) Impersonal-Instrumental**

Vous êtes la/e seul/e médecin dans un hélicoptère de secours lors d’une inondation dévastatrice. Vous avez sauvé huit personnes gravement blessées et vous les maintenez en vie. Tout d’un coup, le pilote vous avertit d’une panne de moteur et ordonne de lâcher du lest ou l’hélicoptère va s’écraser et vous allez tous mourir. Quant à vous, vous ne pouvez pas abandonner l’hélicoptère parce que les blessés ont besoin de vos soins et l’équipement médical lourd est fixé au sol.

Si vous coupez la corde par laquelle monte la dernière personne sauvée, vous allez libérer suffisamment de poids pour garder l'hélicoptère en l'air. Cela va tuer cette personne, mais vous et les huit autres personnes allez être sauvés.

Allégez-vous le poids en coupant la corde afin que vous et les autres huit personnes à bord puissiez rester en l’air?

15) **Personal – accidental**

Vous êtes un/e ingénieur/e de la Station Spatiale Internationale ISS. Tout d’un coup, la soute prend feu. On peut ouvrir la porte extérieure avec un système de sécurité automatique pour libérer de l'oxygène et ainsi éteindre le feu. Cependant, le système ne marche qu'avec la porte intérieure fermée, et il y a encore un mécanicien dans la soute. Vous voyez qu’il n'a pas le temps de se défaire de son scaphandre lourd et qu’il va être coincé s’il essaye de traverser la trappe, provoquant la propagation de l’incendie et votre mort à tous.

Si vous poussez le mécanicien et le renvoyez à la soute, la porte va se fermer. Cela va envoyer le mécanicien dans l’espace, le tuant, mais vous et les dix autres allez être à l’abri.

Eteindrez-vous l’incendie en fermant la porte manuelle, ce qui va envoyer le mécanicien dans l’espace, afin que le feu ne vous atteigne pas, vous et les dix autres astronautes ?

16) **Impersonal – accidental**

Vous êtes un/e ingénieur/e de la Station Spatiale Internationale ISS. Tout d’un coup, la soute prend feu. On peut ouvrir la porte extérieure avec un système de sécurité automatique pour libérer de l'oxygène et ainsi éteindre le feu. Cependant, le système ne marche qu'avec la porte intérieure fermée, et il y a encore un mécanicien dans la soute. Vous voyez qu’il n'a pas le temps de se défaire de son scaphandre lourd et qu’il va être coincé s’il essaye de traverser la trappe, provoquant la propagation de l’incendie et votre mort à tous.

Si vous activez un interrupteur qui ferme la porte intérieure avant que le mécanicien ne l’atteigne, le système de secours sera activé et le feu éteint. Cela va envoyer le mécanicien dans l’espace, le tuant, mais vous et les dix autres allez être sauvés.

Éteindrez-vous le feu en activant l’interrupteur qui va envoyer le mécanicien dans l’espace afin que le feu ne vous atteigne pas, vous et les dix autres astronautes?

**17) Personal – instrumental**

Vous êtes inspecteur/-trice d'une centrale nucléaire. Vous et le directeur êtes en train de la visiter lorsqu’un tuyau d'alimentation surchauffe. Le système de refroidissement d'urgence échoue et une réaction en chaîne aboutissant à une fusion nucléaire est imminente. Le rayonnement libéré sera mortel pour vous et une ville voisine, tuant des milliers de personnes.

Si vous poussez le directeur pour qu’il tombe sur les tuyaux d'alimentation, cela va éliminer assez de chaleur pour arrêter la réaction en chaîne. Il va mourir, mais vous et les gens de la ville voisine serez sauvés.

Refroidissez-vous les tuyaux en poussant le directeur afin que vous et les milliers de personnes de la ville voisine ne soient pas atteints par des radiations nucléaires?

**18) Impersonal – accidental**

Vous êtes inspecteur/trice d'une centrale nucléaire. Vous et le directeur êtes en train de la visiter lorsqu’un tuyau d'alimentation surchauffe. Le système de refroidissement d'urgence échoue et une réaction en chaîne aboutissant à une fusion nucléaire est imminente. Le rayonnement libéré sera mortel pour vous et une ville voisine, tuant des milliers de personnes.

Si vous insérez manuellement de l’azote liquide dans la chambre d'alimentation, cela va éliminer assez de chaleur pour arrêter la réaction en chaîne. Mais un employé est enfermé à l’intérieur. Cela va le tuer, mais vous et les gens de la ville voisine serez sauvés.

Refroidissez-vous les tuyaux en insérant manuellement de l’azote liquide dans la chambre

d’alimentation où se trouve enfermé l’employé afin que vous et les milliers de personnes de la ville voisine ne soient pas atteints par des radiations nucléaires.

**19) Personal – instrumental**

Vous êtes l’expert en explosifs d’une entreprise chargée de démolir un gratte-ciel. Vous êtes en train de vérifier le dernier chargement quand vous voyez un gars qui est sur ​​le point de faire exploser les charges accidentellement. L'explosion entraînerait l'effondrement incontrôlé de la construction sur vous, le garçon et la foule de spectateurs. Le garçon est plusieurs étages plus bas que vous et ne vous entend pas.

Si vous lâchez un lourd bloc de béton sur la tête du gars il va mourir, mais cela va éviter l'explosion et l'effondrement incontrôlé de la construction sur vous, le garçon et la foule de spectateurs.

Stoppez-vous l’action du gars en laissant tomber le bloc sur sa tête pour éviter l’effondrement incontrôlé de la construction sur vous et la foule de spectateurs?

**20) Impersonal-Instrumental**

Vous êtes l’expert en explosifs d’une entreprise chargée de démolir un gratte-ciel. Vous êtes en train de vérifier le dernier chargement quand vous voyez un gars qui est sur ​​le point de faire exploser les charges accidentellement. L'explosion entraînerait l'effondrement incontrôlé de la construction sur vous, le garçon et la foule de spectateurs. Le garçon est plusieurs étages plus bas que vous et ne vous entend pas.

Si vous rétablissez l'électricité dans le bâtiment il va recevoir une décharge électrique car il touche un circuit ouvert, et il va mourir. Cependant cela va éviter l'explosion et l'effondrement incontrôlé de la construction sur vous, le garçon et la foule de spectateurs.

Stoppez-vous l’action du gars en rétablissant l'électricité dans le bâtiment pour éviter l’effondrement incontrôlé de la construction sur vous et la foule de spectateurs?

21) **Personal – instrumental**

Vous travaillez dans la construction. Vous et votre équipe travaillez sur l’échafaudage d’un gratte-ciel. Tout à coup l'échafaudage s’effondre partiellement. Vous et plusieurs collègues pendez à une barre transversale, mais vous êtes trop lourds pour la structure. Un collègue à côté de vous glisse et s’accroche à votre bras. La structure est sur ​​le point de céder.

Si vous frappez votre collègue à plusieurs reprises dans l'estomac et la poitrine il va vous lâcher et tomber. Cela va le tuer mais vous allez alléger suffisamment la structure pour que vous et le reste de l’équipe puissiez accéder à un lieu sûr avant l'effondrement.

Allégez-vous la structure en frappant votre collègue qui va tomber afin que vous et le reste de l'équipe puissiez rester sur la structure?

**22) Impersonal-Instrumental**

Vous travaillez dans la construction. Vous et votre équipe travaillez sur l’échafaudage d’un gratte-ciel. Tout d’un coup l'échafaudage s’effondre partiellement. Vous et plusieurs collègues pendez à une barre transversale, mais vous êtes trop lourds pour la structure. Un collègue à côté de vous glisse et s’accroche à une autre partie de la barre. La structure est sur ​​le point de céder.

Si vous enlevez la goupille pour libérer la partie où s’accroche votre collègue, il va tomber. Cela va le tuer mais vous allez alléger suffisamment la structure afin que vous et le reste de l’équipe puissiez accéder à un lieu sûr avant l'effondrement.

Allégez-vous la structure en enlevant la goupille qui libère la barre de votre collègue qui va tomber, afin que vous et le reste de l'équipe puissiez rester sur la structure?

**23) Personal – accidental**

Vous êtes conducteur/-trice d’autocar et vous conduisez un bus en excursion au zoo d’un jardin d’enfants. Sur le chemin, un défaut dans les roues fait tomber le bus d’un pont dans une rivière sauvage. Vous et trois enfants êtes encore dans le véhicule, les autres ont déjà été emportés par la rivière. Vous attrapez les deux enfants les plus proches et commencez à nager vers la sortie. Le troisième enfant s'accroche à votre jambe. Vous sentez que vous n'êtes pas assez fort/e pour nager contre le courant avec les trois enfants.

Si vous vous débarrassez du troisième enfant et le laissez se noyer vous allez pouvoir lutter contre le courant. Cela va le tuer, mais vous et les deux autres enfants allez être sauvés.

Vous débarrassez-vous du troisième enfant en le laissant se noyer afin que vous et les deux autres enfants puissiez atteindre la surface?

**24) Impersonal-accidental**

Vous êtes conducteur d’autocar et vous conduisez un bus en excursion au zoo d’un jardin d’enfants. Sur le chemin, un défaut dans les roues fait tomber le bus d’un pont dans une rivière sauvage. Vous et trois enfants êtes encore dans le véhicule, les autres ont déjà été emportés par la rivière. Vous attrapez les deux enfants les plus proches et commencez à nager vers la sortie mais elle est bloquée par des sièges cassés.

Si vous libérez la sortie en poussant les sièges dans le couloir, cela va bloquer le troisième enfant et il va se noyer. Cela va le tuer, mais vous et les deux autres enfants allez être sauvés.

Libérez-vous la sortie en bloquant le couloir, laissant le troisième enfant se noyer afin que vous et les deux autres enfants puissiez atteindre la surface?

**(25) Personal – instrumental**

Vous êtes médecin dans un hôpital. Vous avez cinq patients qui vont mourir s’ils ne reçoivent pas une transplantation d’organe tout de suite. Une autre personne vient juste d’être hospitalisée après un grave accident de voiture. Son état est critique et elle va mourir sans intervention chirurgicale immédiate. Vous pouvez probablement la sauver grâce à une opération longue et compliquée.

Si vous lui coupez l’artère carotide discrètement pendant l’opération, il va mourir, mais vous aurez ses organes. Cela va tuer la victime de l’accident, mais vous pourrez sauver les cinq autres patients.

Obtenez-vous ses organes en coupant l'artère carotide de la victime de l'accident afin de pouvoir les donner aux cinq autres patients?

**26) Impersonal-Instrumental**

Vous êtes médecin dans un hôpital. Vous avez cinq patients qui vont mourir s’ils ne reçoivent pas une transplantation d’organe tout de suite. Une autre personne vient juste d’être hospitalisée après un grave accident de voiture. Son état est critique. Pendant l'opération longue et compliquée, vous voyez qu’une infirmière est sur le point d'injecter un médicament inapproprié, létal dans ce cas.

Si vous laissez l’infirmière injecter le médicament, la victime de l'accident va mourir, mais vous aurez ses organes. Cela va le tuer, mais vous allez sauver les cinq autres patients.

Obtenez-vous ses organes en laissant l’infirmière injecter le médicament afin que vous puissiez donner les organes aux autres cinq patients?

27) Personal-27) Impersonal – accidental

Vous faites partie d’une équipe de travailleurs du port qui attache les câbles des grues aux conteneurs des bateaux de transport pour les décharger. Vous et vos collègues venez d’attacher les câbles à un conteneur et vous êtes en train de monter dessus pour vous assurer que le déchargement se déroule correctement. Tout d’un coup, la lumière rouge d’alarme s’allume indiquant qu’il y a un câble qui va lâcher. Vous voyez que c'est à cause de deux de vos collègues qui sont en train de se battre. Le conteneur bascule dangereusement au-dessus de cinq autres collègues en bas sur le pont.

Si vous poussez les deux combattants pour les séparer, vous évitez que le conteneur ne tombe. Cependant, l’un des deux ne porte pas son harnais de sécurité et va tomber. Cela va le tuer, mais les cinq collègues sur le pont seront sauvés.

Arrêtez-vous le basculement du conteneur en poussant les deux collègues ce qui fera tomber celui qui n’a pas de harnais, afin que le conteneur n’écrase pas les cinq collègues d’en bas?

**28) Impersonal – accidental**

Vous faites partie d’une équipe de travailleurs du port qui attache les câbles des grues aux conteneurs des bateaux de transport pour les décharger. Vous et vos collègues venez d’attacher les câbles à un conteneur et vous êtes en train de monter dessus pour vous assurer que le déchargement se déroule correctement. Tout à coup, la lumière rouge d’alarme s’allume indiquant qu’il y a un câble qui va lâcher. Si le câble se rompt au-dessus du pont, le conteneur va tomber sur cinq collègues.

Vous pouvez appuyer sur le bouton d’urgence ce qui renverrait le conteneur vers la cale. Vous et vos collègues seriez suspendus par vos harnais de sécurité, mais il y a encore un collègue dans la cale. La chute du conteneur va le tuer, mais les cinq collègues sur le pont seront sauvés.

Appuyez-vous sur le bouton d’urgence pour renvoyer le conteneur vers la cale, ce qui le fera tomber sur le collègue en bas dans la cale et non pas sur les cinq collègues du pont?

**29) Personal – accidental**

Une épidémie s’est déclenchée dans le monde, ce qui a déjà tué des milliers de personnes. Vous êtes chercheur et vous avez développé deux substances dans le laboratoire. Les deux sont étiquetées à tort comme vaccins. Une d'elles est certainement le vaccin qui pourrait sauver des milliers de personnes, l'autre est une substance mortelle.

Si vous injectez les substances à vos deux assistants de laboratoire, une à chacun, vous allez pouvoir identifier le vaccin. L’un des deux va mourir mais grâce au vaccin vous pourrez commencer à sauver des milliers de vies.

Identifiez-vous le vaccin en injectant les substances à vos deux assistants, l’un des deux sera empoisonné mais des milliers de personnes pourront être vaccinées?

**30) Impersonal-accidental**

Une épidémie s’est déclenchée dans le monde, ce qui a déjà tué des milliers de personnes. Vous êtes chercheur et vous avez développé deux substances dans le laboratoire. Les deux sont étiquetées à tort comme vaccins. L’une d'elles est certainement le vaccin qui pourrait sauver des milliers de personnes, l'autre est une substance mortelle.

Si vous permettez qu’une infirmière injecte les substances à deux patients, une à chacun, vous allez pouvoir identifier le vaccin. L’un des deux va mourir, mais grâce au vaccin vous pourrez commencer à sauver des milliers de vies.

Identifiez-vous le vaccin en ordonnant que l’infirmière injecte les substances à deux de vos patients, l’un d’eux sera empoisonné, mais des milliers de personnes pourront être vaccinées?

**31) Personal – instrumental**

Un tramway hors de contrôle se dirige à toute vitesse vers cinq travailleurs qui vont mourir si le tram continue son chemin. Vous êtes à côté des voies, mais trop loin pour les avertir. Auprès de vous il y a un inconnu très grand.

Si vous poussez l'inconnu sur les voies, faisant dérailler le tramway, vous allez éviter la mort certaine des cinq travailleurs. Cela va tuer l'inconnu, mais vous allez sauver les cinq travailleurs.

Poussez-vous l'inconnu sur les voies afin que le tram n'atteigne pas les cinq travailleurs?

**32) Impersonal – accidental**

Un tramway hors de contrôle se dirige à toute vitesse vers cinq travailleurs qui vont mourir si le tram continue son chemin. Vous êtes à côté des voies, mais trop loin pour les avertir. Auprès de vous il y a un levier avec lequel l’on peut rediriger le tram sur ​​une autre voie où ne travaille qu’un travailleur.

Si vous appuyez sur le levier le tram sera redirigé vers la voie de ce travailleur, évitant la mort certaine des cinq travailleurs. Cela va tuer ce travailleur, mais vous allez sauver les cinq autres.

Appuyez-vous sur le levier pour changer le tram de voie, en écrasant le travailleur isolé, afin que le tram n'atteigne pas les autres cinq travailleurs?

**33) Personal – instrumental**

Vous et un collège de la recherche avez découvert une nouvelle source d'énergie qui est à bas prix, propre et sûre. L’invention pourrait en terminer avec la pollution et la pauvreté dans le monde. Cependant, votre collègue veut vendre la découverte, et vous savez qu’il projette de contacter des acheteurs potentiels au plus tard aujourd’hui. Mais vous savez de façon sûre que ceux-ci vont utiliser l’invention comme une arme terrible.

Si vous empoisonnez votre collègue avec un poison qui se trouve dans votre laboratoire, vous allez empêcher que l’invention ne tombe dans de mauvaises mains. Le poison va le tuer, mais vous allez éviter que la découverte soit utilisée comme une arme contre des milliers de personnes.

Empêchez-vous que l’invention ne tombe dans des mauvaises mains en empoisonnant à votre collègue afin que l’invention ne soit pas utilisée comme une arme terrible contre des milliers de gens?

**34) Impersonal-Instrumental**

Vous et un collège de la recherche avez découvert une nouvelle source d'énergie qui est à bas prix, propre et sûre. L’invention pourrait en terminer avec la pollution et la pauvreté dans le monde. Cependant, votre collègue veut vendre la découverte, et vous savez qu’il projette de contacter des acheteurs potentiels au plus tard aujourd’hui par e-mail. Mais vous savez de façon sûre que ceux-ci vont utiliser l’invention comme une arme terrible.

Si vous libérez un gaz inflammable dans le laboratoire cela va causer une explosion lorsque votre collègue connectera son ordinateur. L’explosion va le tuer, mais vous allez éviter que la découverte soit utilisée comme une arme contre des milliers de personnes.

Empêchez-vous que l’invention ne tombe dans des mauvaises mains en libérant le gaz dans le laboratoire pour faire exploser l’ordinateur de votre collègue afin que l’invention ne soit pas utilisée comme une arme terrible contre des milliers de gens?

**35) Personal – instrumental**

Vous êtes un motocycliste et vous participez à un rassemblement de motards. Vous conduisez devant un groupe important d’autres motards quand tout-à-coup vous voyez qu’un motard d’en face est en train de perdre le contrôle de sa moto. Vous accélérez pour l’aider, mais vous vous rendez compte qu'il pourrait tomber à tout moment, provoquant un accident en chaîne qui va tuer un groupe de dix motards derrière vous.

Si vous le forcez en dehors de la route, il va s'écraser contre les arbres. A cette vitesse, ce motard va mourir, mais vous allez éviter l’accident en chaîne.

Évitez-vous l’accident en chaîne en forçant ce motard en dehors de la route pour que le groupe des dix motards derrière vous n’entre pas en collision avec lui?

**36) Impersonal-instrumental**

Vous êtes un motocycliste et vous participez à un rassemblement de motards. Vous conduisez devant un groupe important d’autres motards quand tout-à-coup, vous voyez qu’un motard en face est en train de perdre le contrôle de sa moto et tombe sur la route. Vous pouvez facilement l'esquiver, mais les motards derrière vous vont chuter en essayant de l'éviter aussi, provoquant un accident en chaîne mortel.

Si vous lancez votre moto contre la moto de ce motard, tout en sautant dans le fossé, vous allez avertir les autres à temps. Cela va tuer le motard tombé, mais vous allez sauver les dix autres motards.

Avertissez-vous les autres de l’accident imminent en entrant en collision avec le motard accidenté pour que les dix motards derrière vous évitent l’accident?

**37) Personal – instrumental**

Vous êtes le/la leader d'un petit groupe de soldats. Vous revenez d'une mission en territoire ennemi quand un de vos hommes tombe dans un piège, qui le retient par la jambe, blessée gravement. Vous ne pouvez pas le libérer sans le tuer, mais si vous le laissez là, les troupes ennemies vont le torturer jusqu’à ce qu’il révèle le site d'un camp de base allié important. En conséquence, les ennemis pourront attaquer le camp et tuer les 10 soldats de la troupe alliée. Le soldat vous supplie de ne pas l’abandonner, mais les troupes ennemies s’approchent et il est dangereux de rester là pour vous et vos hommes.

Si vous poignardez le soldat vous-même, vous évitez qu’il soit torturé et qu’il ne révèle le site de la troupe alliée. Cela va le tuer, mais vous allez sauver les 10 soldats de la troupe alliée.

Évitez-vous que le soldat soit torturé en le poignardant vous-même pour qu’il ne puisse pas révéler la localisation des 10 soldats de la troupe alliée?

**38) Impersonal – Instrumental**

Vous êtes le/la leader d'un petit groupe de soldats. Vous retournez d'une mission en territoire ennemi quand un de vos hommes tombe dans un piège, qui retient sa jambe, la blessant gravement. Vous ne pouvez pas le libérer sans le tuer, mais si vous le laissez là, les troupes ennemies vont le torturer jusqu’à ce qu’il révèle le site d'un camp de base allié important. En conséquence les ennemis pourront attaquer le camp et tuer les 10 soldats de la troupe alliée. Le soldat vous supplie de ne pas l’abandonner, mais les troupes ennemies s’approchent et il est dangereux de rester là pour vous et vos hommes.

Si vous indiquez à l’ordinateur cible la position du blessé, le secteur sera bombardé et il va mourir sans être torturé et sans révéler le site de la troupe alliée. Cela va le tuer, mais vous allez sauver les 10 soldats de la troupe alliée.

Évitez-vous que le soldat soit torturé en programmant l’ordinateur cible pour qu’il ne puisse pas révéler la localisation des 10 soldats de la troupe alliée?

**39) Personal – instrumental**

Vous êtes gardien de nuit dans un hôpital. Un produit chimique a été renversé dans une chambre avec six patients. Le produit est très toxique car quand il est exposé à l’oxygène il s'évapore y se transforme en un gaz toxique. Ce gaz va tuer tous les patients dans la salle. Il est impossible de le nettoyer avec des serviettes ou des linges conventionnels. Toutefois, la peau humaine l’absorbe rapidement.

Si vous descendez un des patients de son lit et le placez sur le produit vous allez éviter que celui-là s’évapore. Le patient va mourir, mais vous allez sauver les cinq autres.

Placez-vous un patient sur ​​le produit afin que celui-ci ne s’évapore pas et que les cinq autres patients ne soient pas intoxiqués?

**40) Impersonal – accidental**

Vous êtes gardien de nuit dans un hôpital. Un produit chimique a été renversé dans une chambre avec six patients. Le produit est très toxique car quand il est exposé à l’oxygène il s'évapore y se transforme en un gaz toxique. Ce gaz va tuer tous les patients dans la salle. Il est impossible de le nettoyer avec des serviettes ou des linges conventionnels.

Si vous activez le système de ventilation le gaz sera extrait de la chambre, mais il sera redirigé vers une chambre sur l’autre étage où se trouve un patient que vous n’allez pas pouvoir sortir à temps. Ce patient va mourir, mais vous allez sauver les cinq autres.

Extrayez-vous le gaz de la salle en activant le système de ventilation, redirigeant ainsi le gaz vers la chambre de l’autre patient, et évitant que les six patients ne soient intoxiqués?

**41) Personal – instrumental**

Vous êtes dans un bateau avec un guide sur un lac en Alaska. Le bateau de trois enfants s’est renversé et ils sont en danger de mourir de froid. Vous commencez à ramer vers eux mais vous réalisez que le bateau ne va pas résister à votre poids, au poids du guide et à celui des trois enfants. Vous sauvez les enfants mais l’un d’eux pèse très lourd et s’il reste à bord votre bateau va chavirer et les trois enfants vont mourir. Vous ne pouvez pas quitter le bateau parce que vous ramez et le guide fait la RCR à un des enfants.

Si vous jetez le plus lourd des enfants à l’eau vous allez pouvoir atteindre la côte. Cela va tuer cet enfant mais vous allez sauver les deux autres.

Allégez-vous le bateau en jetant l’un des enfants à l’eau afin de pouvoir atteindre la côte avez les deux autres ?

**42) Impersonal- accidental**

Vous êtes dans un bateau avec un guide sur un lac en Alaska. Le bateau de trois enfants s’est renversé et ils sont en danger de mourir de froid. Vous commencez à ramer vers eux mais vous réalisez que le bateau ne va pas résister à votre poids, au poids du guide et à celui des trois enfants. Vous sauvez deux des enfants mais l’un d’eux est toujours dans l’eau. Si vous le prenez à bord, votre bateau va chavirer et les trois enfants vont mourir. Vous ne pouvez pas quitter le bateau parce que vous ramez et le guide fait la RCR à un des enfants.

Si vous ramez rapidement vers la côte, laissant le troisième enfant dans l’eau, vous allez éviter que votre bateau ne se renverse. Cela va tuer l’enfant dans l’eau, mais vous allez sauver les deux autres.

Évitez-vous le chavirage de votre bateau en laissant dans l’eau le troisième enfant qui mourra de froid, afin que vous puissiez atteindre la côte avec les deux autres enfants?

**43) Personal – instrumental**

Vous travaillez dans une mine. Pour sortir il faut monter dans les cabines d’un simple ascenseur à cordes. Chaque cabine résiste au poids de trois personnes. Vous avez terminé la journée et tout le monde remonte à la surface avec les cabines. En attendant la vôtre, tout d’un coup, vous voyez que le câble qui supporte toutes les cabines est sur ​​le point de se rompre. Il y a à ce moment 13 mineurs dans les cabines.

Si vous renversez la dernière cabine dans laquelle n’est monté qu’un seul mineur, vous allez alléger suffisamment le poids pour que le câble ne se rompe pas et éviter ainsi que les 12 autres mineurs ne fassent une chute mortelle. Le mineur seul sera tué, mais vous allez sauver les 13 autres.

Allégez-vous le poids nécessaire en renversant la cabine du dernier mineur, afin que les cabines des autres 13 mineurs ne chutent pas?

**44) Impersonal- instrumental**

Vous travaillez dans une mine. Pour sortir il faut monter dans les cabines d’un simple ascenseur à cordes. Chaque cabine résiste au poids de trois personnes. Vous avez terminé la journée et tout le monde remonte à la surface avec les cabines. En attendant la vôtre, tout d’un coup vous voyez que le câble qui supporte toutes les cabines est sur ​​le point de se rompre. Il y a à ce moment, 10 mineurs dans les cabines.

Si vous tirez le frein de secours, la dernière cabine dans laquelle n’est monté qu’un seul mineur sera automatiquement renversée et ainsi vous allez alléger suffisamment le poids pour que le câble ne se rompe pas et éviter ainsi que les 10 autres mineurs ne fassent une chute mortelle. Le mineur seul sera tué, mais vous allez sauver les 10 autres.

Allégez-vous le poids nécessaire en tirant le frein de secours qui fera que la cabine du dernier mineur sera renversée, afin que les cabines des 10 autres ne chutent pas?

**45) Personal – accidental**

Vous êtes un électricien qui a été engagé pour réparer un problème électrique dans la maison d'un riche magnat de l'acier qui est en phase terminale d'un cancer. Récemment il a été annoncé qu’à sa mort, sa fortune sera donnée à un hôpital pour enfants de la région. Vous savez qu'il y a 109 enfants qui pourraient être sauvés avec cet argent, mais qui ne peuvent attendre beaucoup plus parce qu'ils sont sur ​​le point de mourir.

Vous pouvez étouffer le magnat quand il dort en lui couvrant la bouche et le nez avec vos mains gantées. Cela va le tuer, mais vous allez sauver la vie des 109 enfants.

Accélérez-vous la donation en étouffant cet homme pour que les 109 enfants malades reçoivent la donation à temps?

**46) Impersonal-Instrumental**

Vous êtes un électricien qui a été engagé pour réparer un problème électrique dans la maison d'un riche magnat de l'acier qui est en phase terminale d'un cancer. Récemment il a été annoncé qu’à sa mort, sa fortune sera donnée à un hôpital pour enfants de la région. Vous savez qu'il y a 112 enfants qui pourraient être sauvés avec cet argent, mais qui ne peuvent attendre beaucoup plus parce qu'ils sont sur ​​le point de mourir.

Vous pouvez provoquer un court-circuit au lieu de réparer le problème ce qui fera que le respirateur du magnat tombera en panne. Cela va le tuer, mais vous allez sauver la vie des 112 enfants.

Accélérez-vous la donation en provoquant un court-circuit, afin que le respirateur du magnat tombe en panne et que les 112 enfants malades reçoivent la donation à temps?

47) **Personal – instrumental**

Vous êtes un officier de l'armée pendant une guerre. Vos soldats ont découvert un espion important, capturé et blessé dans un immeuble effondré. Par la radio, vous avez été informé que les soldats ennemis ont l’intention de faire une attaque surprise sur une ville voisine, mais vous ne savez pas laquelle. Ils vont tuer des milliers de civils si vous et vos soldats ne les arrêtent pas. L'espion sait de quelle ville il s’agit, mais vous l’avez interrogé et il ne vous le dit pas. Il mourra bientôt à cause de ses blessures.

Si vous le torturez il va vous révéler de quelle ville il s’agit. Cela va le tuer, mais vous allez sauver des milliers de civils.

Torturez-vous l’espion pour qu’il vous révèle l’information, afin que vous puissiez prévenir l’attaque sur la ville voisine?

**48) Impersonal-Instrumental**

Vous êtes un officier de l'armée pendant une guerre. Vos soldats ont trouvé un espion important, capturé et blessé dans un immeuble effondré. Par la radio vous avez été informé que les soldats ennemis ont l’intention de faire une attaque surprise sur une ville voisine, mais vous ne savez pas laquelle. Ils vont tuer des milliers de civils si vous et vos soldats ne les arrêtent pas. L'espion sait de quelle ville il s’agit, mais vous l’avez interrogé et il ne vous le dit pas. Il mourra bientôt à cause de ses blessures. Des rats ont commencé à le ronger et il souffre beaucoup.

Si vous laissez les rats poursuivre leur torture, l’espion va vous révéler de quelle ville il s’agit. Cela va le tuer, mais vous allez sauver des milliers de civils.

Laissez-vous les rats torturer l’espion pour qu’il vous révèle l’information et que vous puissiez prévenir l’attaque sur la ville voisine?

**49) Personal – Others Beneficial – Inevitable (instrumental)**

Vous êtes médecin. Un de vos patients qui a le SIDA est sur ​​le point de recevoir une transfusion avant sa sortie de l'hôpital. Il vous a confié sous la relation confidentielle médecin-patient, qu’en sortant il envisage de contaminer autant de personnes que possible, en commençant cet après-midi-là. Vous n'avez aucun moyen juridique pour l'arrêter parce que la police ne peut pas agir sur la base d’informations protégées.

Si vous contaminez ses médicaments avec un poison non-détectable vous allez éviter qu’il ne contamine d’autres personnes. Cela va le tuer, mais vous allez sauver beaucoup d’autres personnes.
Contaminez-vous les médicaments de votre patient pour éviter la propagation du SIDA à de nombreuses autres personnes ?

**50) Impersonal – Others Beneficial – Inevitable (instrumental)**

Vous êtes médecin. Un de vos patients qui a le SIDA est sur ​​le point de recevoir une transfusion avant sa sortie de l'hôpital. Il vous a confié sous la relation confidentielle médecin-patient, qu’en sortant il envisage de contaminer autant de personnes que possible, en commençant cet après-midi là. Vous n'avez aucun moyen juridique pour l'arrêter parce que la police ne peut pas agir sur la base d’informations protégées.

Si vous changez son groupe sanguin dans la base de l'hôpital il va recevoir une transfusion mortelle et vous allez éviter qu’il ne contamine d’autres personnes. Cela va le tuer, mais vous allez sauver beaucoup d’autres personnes.

Changez-vous le groupe sanguin de votre patient dans l’ordinateur afin d’éviter la propagation du SIDA à de nombreuses autres personnes?

**51) Personal – Others Beneficial – Avoidable (instrumental)**

Vous êtes en train de négocier avec un homme perturbé et armé sur le toit d'un bâtiment. En bas il y a une foule de gens car c’est le Festival des arts et de la musique de la ville. Il veut tirer à vue sur la foule et vous n’avez pas pu le convaincre de ne pas le faire. La foule ignore la menace et vous n’avez aucun moyen de les avertir. De sa position, il pourrait tuer une douzaine de personnes, sans compter ceux qui seront blessés ou tués pendant le chaos qui s’ensuivrait.

Si vous courez rapidement vers lui et le poussez, il va tomber du toit et ne pourra plus tirer sur la foule. Cela va le tuer, mais vous allez sauver de nombreuses vies.

Prévenez-vous la fusillade et le chaos en poussant cet homme du toit afin qu’il ne tire pas à vue sur la foule?

**52) Impersonal – Others Beneficial – Avoidable (instrumental)**

Vous êtes en train de négocier avec un homme perturbé et armé sur le toit d'un bâtiment. En bas il y a une foule de gens car c’est le Festival des arts et de la musique de la ville. Il veut tirer à vue sur la foule et vous n’avez pas pu le convaincre de ne pas le faire. La foule ignore la menace et vous n’avez aucun moyen de les avertir. De sa position, il pourrait tuer une douzaine de personnes, sans compter ceux qui seront blessés ou tués pendant le chaos qui s’ensuivrait.

Si vous avertissez la police que la négociation n’a pas échoué, ils vont l’abattre et il ne pourra plus tirer sur la foule. Cela va le tuer, mais vous allez sauver de nombreuses vies.

Evitez-vous la fusillade et le chaos en avertissant la police afin que l’homme ne puisse pas tirer sur la foule?
