# Supplementary material for: Moral judgment reloaded: a moral dilemma validation study
Source: Front Psychol. 2014 Jul 1;5:607. doi: 10.3389/fpsyg.2014.00607 (PMC4077230; doi:10.3389/fpsyg.2014.00607)
Supplement: Supplementary file 9 [file DataSheet9.DOC]

**German**

**DILEMMATA**

**1) Personal - Instrumental**

Du und fünf weitere Personen seid in einem brennenden Gebäude gefangen. Es gibt nur einen Notausgang, durch den ihr alle entkommen könntet. Er ist aber durch brennende Trümmer blockiert. Eine weitere verletzte Person versucht durch ein Loch im unteren Teil des Ausgangs zu kriechen. Du und die fünf Personen hinter dir habt nicht die Zeit das Gleiche zu tun.

Wenn du den verletzten Mann benutzt um die Schuttblockade zu durchbrechen, werdet ihr entkommen können. Dies wird ihn sicher töten, aber du und die fünf Leute hinter dir werdet euch retten können.

Durchbrichst du den Schutt mit der verletzten Person damit du und die anderen fünf Personen entkommen könnt?

**2) Impersonal- Accidental**

Du und fünf weitere Personen seid in einem brennenden Gebäude gefangen. Es gibt nur einen Notausgang, durch den ihr alle entkommen könntet, er ist aber durch brennende Trümmer blockiert. Eine weitere verletzte Person versucht durch ein Loch im unteren Teil des Ausgangs zu kriechen. Du und die fünf Personen hinter dir habt nicht die Zeit das Gleiche zu tun.

Wenn du das Notfallsystem aktivierst wird der Sauerstoff aus dem Flur entfernt und das Feuer wird gelöscht, aber der Verwundete wird keine Luft mehr bekommen. Das wird ihn sicher töten, aber du und die fünf Personen hinter dir könnt euch retten.

Löschst du das Feuer indem du das Notfallsystem aktivierst, wodurch der Verwundete keine Luft mehr bekommen wird, damit du und die anderen fünf Personen entkommen könnt?

**3) Personal – accidental**

Feindliche Soldaten haben dein Dorf belagert und werden alle Zivilisten über zwei Jahre töten. Du und zehn Nachbarn versteckt euch in zwei Räumen im Keller eines großen Hauses. Ihr hört die Stimmen der Soldaten, die ins Haus gekommen sind um nach Wertgegenständen zu suchen. Dein Säugling beginnt laut zu schreien. Das Schreien wird die Aufmerksamkeit der Soldaten auf euch ziehen, welche zwar nicht dein Baby, schon aber dich und die anderen Flüchtlinge in beiden Räumen töten werden.

Wenn du dem Baby den Mund zuhältst wird das Schreien gedämpft, aber es wird so keine Luft mehr bekommen. Das wird das Baby töten, aber du und die anderen zehn Nachbarn könnt euch retten.

Dämpfst du das Schreien, indem du deinem Baby den Mund zuhältst, woduch es keine Luft mehr bekommen wird, damit die Soldaten dich und die zehn Nachbarn nicht finden?

**4) Impersonal- accidental**

Feindliche Soldaten haben dein Dorf belagert und werden alle Zivilisten über zwei Jahre töten. Du und zehn Nachbarn versteckt euch in zwei Räumen im Keller eines großen Hauses. Ihr hört die Stimmen der Soldaten, die in das Haus gekommen sind um nach Wertgegenständen zu suchen. Dein Säugling beginnt laut zu schreien. Das Schreien wird die Aufmerksamkeit der Soldaten auf euch ziehen, welche zwar nicht dein Baby, schon aber dich und die anderen Flüchtlinge in beiden Räumen töten werden.

Wenn du einen lauten Heizungskessel aktivierst wird das Schreien gedämpft. Allerdings erzeugt er eine für Kinder und Erwachsene unangenehme, aber für Babys tödliche Hitze. Das wird dein Baby töten, aber dich und die zehn Nachbarn retten.

Dämpfst du das Schreien, indem du den Heizungskessel aktivierst, wodurch das Baby ersticken wird, damit man dich und die zehn Nachbarn nicht findet?

**5) Personal – accidental**

Du gehörst zur Besatzung eines U-Bootes, das sich unter einem großen Eisberg befindet. Eine Explosion hat das Boot beschädigt. Mehrere Kollegen sind verletzt und der einzige Zugang zwischen dem oberen und unteren Teil des Bootes ist verschüttet. Du und zehn weitere Überlebende befindet euch im oberen Teil. Hier gibt es aber nicht genug Sauerstoff für euch alle, bis ihr die Wasseroberfläche erreicht. Ein einzelner Kollege liegt bewusstlos im unteren Teil. Hier gibt es noch genügend Sauerstoff.

Wenn du die Notfallklappe zwischen den beiden Teilen aufschiebst, wird die Luft durchgelassen. Allerdings wird die Klappe auf den Kollegen im unteren Teil fallen und ihn töten, aber dies wird dich und die zehn Überlebenden retten.

Lässt du die Luft durch, indem du die Notfallklappe öffnest, die auf den Kollegen fallen wird, damit ihr, du und die zehn Überlebenden, genug Sauerstoff bekommt?

**6) Impersonal- accidental**

Du gehörst zur Besatzung eines U-Bootes, das sich unter einem großen Eisberg befindet. Eine Explosion hat das Boot beschädigt. Mehrere Kollegen sind verletzt und der einzige Zugang zwischen dem oberen und unteren Teil des Bootes ist verschüttet. Du und zehn weitere Überlebende befindet euch im oberen Teil. Hier gibt es aber nicht genug Sauerstoff für euch alle, bis ihr die Wasseroberfläche erreicht. Ein einzelner Kollege liegt bewusstlos im unteren Teil. Hier gibt es noch genügend Sauerstoff.

Wenn du einen Notfallschalter betätigst, wird eine Notfallklappe zwischen den beiden Teilen geöffnet und die Luft durchgelassen. Allerdings wird die Klappe auf den Kollegen fallen und ihn töten, aber es wird dich und die zehn Überlebenden retten.

Betätigst du den Notfallschalter, welcher die Klappe auf den Kollegen fallen lassen wird, damit ihr, du und die zehn Überlebenden, genug Sauerstoff bekommt?

**7) Personal** – **instrumental**

Du und zehn Taucher seid Teil eines Teams der Vereinten Nationen was Seeminen aus dem Zweiten Weltkrieg unschädlich macht. Ein Teammitglied wurde verletzt und das Blut hat mehrere Haie angelockt. Du hast ein Unterwassergewehr, aber nur eine Harpune und es sind viele Haie. Der blutende Taucher schwimmt auf den letzten Schutzkäfig zu und wird diesen vor dir und den anderen erreichen. Die Haie folgen der Blutspur und sind zu nah, als dass du und die anderen Taucher entkommen könntet.

Wenn du auf den Taucher schießt wird es ihn töten und die Haie werden innehalten um ihn zu fressen, aber du und die zehn Taucher könnt euch retten.

Lässt du die Haie den verletzten Taucher fressen, indem du auf ihn schießt, damit du und die anderen zehn Taucher den Schutzkäfig erreichen könnt?

**8) Impersonal-Instrumental**

Du und zehn Taucher seid Teil eines Teams der Vereinten Nationen was Seeminen des Zweiten Weltkriegs unschädlich macht. Ein Teammitglied wurde verletzt und das Blut hat mehrere Haie angelockt. Du hast ein Unterwassergewehr, aber nur eine Harpune und es sind viele Haie. Der blutende Taucher schwimmt auf den letzten Schutzkäfig zu und wird diesen vor dir und den anderen erreichen. Die Haie folgen der Blutspur und sind zu nah, als dass du und die anderen Taucher entkommen könntet.

Wenn du per Fernbedienung eine der Minen detonieren lässt, wird es ihn töten und die Haie werden innehalten um ihn zu fressen, aber du und die zehn Taucher werdet euch retten können.

Lässt du die Haie den verletzten Taucher fressen, indem du die Mine per Fernbedienung detonierst, damit du und die anderen zehn Taucher den Schutzkäfig erreichen könnt?

**9) Personal – instrumental**

Du arbeitest in einem Waisenhaus in einem kriegsgebeutelten Land. Die Rebellen haben die Regierung übernommen und töten alle, die das Regime nicht unterstützen. Außerdem führen sie einen Völkermörd gegen eine bestimmte ethnische Gruppe durch. Du und die elf Waisen des Waisenhauses habt die Rebellion nicht unterstützt und darüber hinaus hast du ein Kind dieser ethnischen Gruppe in deinem Heim. Es gibt keine Möglichkeit, dass du und die Waisenkinder entkommen könnt und mit Sicherheit werden die Soldaten euch alle töten.

Wenn du das Kind der verfolgten Volksgruppe mit einem Kissen erstickst, während es schläft, werden die Soldaten glauben, dass du ihr Regime unterstützt. Das wird das Kind töten, aber es wird dich und die restlichen zehn Kinder retten.

Lässt du die Soldaten in dem Glauben, dass du ihr Regime unterstützt, indem du das genannte Kind erstickst, damit sie Dich und anderen zehn Kindern verschonen?

**10) Impersonal – Instrumental**

Du arbeitest in einem Waisenhaus in einem vom Krieg gebeutelten Land. Die Rebellen haben die Regierung übernommen und töten alle, die das Regime nicht unterstützen. Außerdem führen sie einen Völkermörd gegen eine bestimmte ethnische Gruppe durch. Du und die elf Waisen des Waisenhauses habt die Rebellion nicht unterstützt und darüber hinaus hast du ein Kind dieser ethnischen Gruppe in deinem Heim. Es gibt keine Möglichkeit, dass du und die Waisenkinder entkommen könnt und mit Sicherheit werden die Soldaten euch alle töten.

Wenn du einer Krankenschwester befiehlst dem Kind der verfolgten Volksgruppe eine tödliche Überdosis von Schlaftabletten zu geben, werden die Soldaten glauben, dass du ihr Regime unterstützt. Das wird das Kind töten, aber es wird dich und die restlichen zehn Kinder retten.

Lässt du die Soldaten in dem Glauben, dass du ihr Regime unterstützt, indem du der Krankenschwester befiehlst dem genannten Kind eine Überdosis zu geben, damit sie Dich und anderen zehn Kindern verschonen?

**11) Personal – instrumental**

Du bist ein/e ehrenamtliche/r Mitarbeiter/in eines Gesundheitszentrums in einem afrikanischen Dorf. Ein Mann aus dem Nachbardorf hat sich mit dem Ebola-Virus infiziert. Die Krankheit ist extrem ansteckend, unheilbar und fast immer tödlich. Unerwarteter Weise hat er sie schon einen Monat überlebt. D.h., dass er wahrscheinlich immun gegen die tödlichen Auswirkungen des Virus ist. Dennoch glaubt er fälschlicherweise, dass dein Gesundheitszentrum ihn vollständig heilen könnte. Du siehst, wie er sich nähert und weißt, dass wenn er das Dorf betritt, er 119 Unschuldige anstecken wird , die im Gegensatz zu ihm sterben werden.

Wenn du ihn mit der Pistole des Zentrums erschießt, wirst du verhindern, dass er dich und den Rest des Dorfes mit dem Virus ansteckt. Das wird ihn töten, aber es wird dich und die 119 Bewohner des Dorfes retten.

Verhinderst du das der Mann das Dorf betritt, indem du ihn erschießt, damit er dich und die 119 Personen des Dorfes nicht ansteckt?

**12) Impersonal-Instrumental**

Du bist ein/e ehrenamtliche/r Mitarbeiter/in eines Gesundheitszentrums in einem afrikanischen Dorf. Ein Mann aus dem Nachbardorf hat sich mit dem Ebola-Virus infiziert. Die Krankheit ist extrem ansteckend, unheilbar und fast immer tödlich. Unerwarteter Weise hat er sie schon einen Monat überlebt, d.h. dass er wahrscheinlich immun gegen die tödlichen Auswirkungen des Virus ist. Dennoch glaubt er fälschlicherweise, dass dein Gesundheitszentrum ihn vollständig heilen könnte. Du siehst wie er sich nähert und weisst, dass wenn er das Dorf betritt, wird er 119 Unschuldige anstecken, die, im Gegensatz zu ihm, sterben werden.

Wenn du den Gemeinderat informierst, wird dieser seine Erschießung veranlassen, um zu verhindern, dass er dich und den Rest des Dorfes mit dem Virus ansteckt. Das wird ihn töten, aber es wird dich und die 119 Bewohner des Dorfes retten.

Verhinderst du, dass der Mann das Dorf betritt indem du den Gemeinderat informierst, damit seine Erschießung veranlasst wird, so dass er dich und die 119 Personen nicht anstecken kann?

**13) Personal – instrumental**

Du bist ein/e Arzt/Ärztin allein in einem Rettungshubschrauber während einer verheerenden Flutkatastrophe. Du hast acht Schwerverletzte gerettet und erhältst sie am Leben. Plötzlich meldet der Pilot einen Motorschaden und befielt, dass der Hubschrauber von Ballast befreit werden muss, da er sonst abstürzen wird und ihr alle sterben werdet. Du kannst den Hubschrauber nicht verlassen, denn die Verwundeten brauchen deine Hilfe und die schweren medizinischen Gerätschaften sind festgeschraubt.

Wenn du einen Verwundeten herausschubst, befreist du den Hubschrauber von ausreichend Ballast um ihn in der Luft zu halten. Dies wird den Verwundeten töten, aber es wird dich und die acht Personen an Bord retten.

Befreist du den Hubschrauber von Ballast, indem du einen der Verwundeten herausschubst, um den Hubschrauber mit Dir und den acht Personen in der Luft zu halten?

**14) Impersonal-Instrumental**

Du bist ein/e Arzt/Ärztin allein in einem Rettungshubschrauber während einer verheerenden Flutkatastrophe. Du hast acht Schwerverletzte gerettet und erhältst sie am Leben. Plötzlich meldet der Pilot einen Motorschaden und befielt, dass der Hubschrauber von Ballast befreit werden muss, da er sonst abstürzen wird und ihr alle sterben werdet. Du kannst den Hubschrauber nicht verlassen, denn die Verwundeten brauchen deine Hilfe und die schweren medizinischen Gerätschaften sind festgeschraubt.

Wenn du das Tau durchschneidest, mit dem die letzte gerettete Person hochgezogen wird, reduzierst du genügend Gewicht, um den Hubschrauber in der Luft zu halten. Dies wird den Verletzten töten, aber es wird dich und die acht Personen an Bord retten.

Reduzierst du das Gewicht, indem du das Tau durchschneidest an dem die letzte Person hochgezogen wird, um den Hubschrauber mit Dir und den acht Personen in der Luft zu halten

**15) Personal – accidental**

Du bist ein/e Ingenieur/in in der Internationalen Raumstation ISS. Plötzlich fängt der Laderaum Feuer. Das automatische Sicherheitssystem kann den Sauerstoff ablassen und so das Feuer löschen. Allerdings wird dafür die Außentür geöffnet und das funktioniert nur wenn die Innenluke geschlossen ist. Hinter der Luke befindet sich aber noch ein Mechaniker. Du siehst, dass ihm nicht die Zeit bleibt, um die schwere Ausrüstung auszuziehen und dass er steckenbleiben wird, wenn er versucht durch die Innenluke zu kommen. Das wird dazu führen, dass sich das Feuer ausbreitet und euch alle tötet.

Wenn du den Mechaniker zurück in den Laderaum schubst, wird sich die Innenluke schließen lassen, das Notfallsystem aktiviert und das Feuer gelöscht. Dies wird den Mechaniker in den Weltraum schleudern und ihn töten, aber es wird dich und die verbleibenden zehn Astronauten retten.

Löschst du das Feuer indem du die Innenluke von Hand schließt, was den Mechaniker in den Weltraum schleudert, damit das Feuer dich und die zehn Astronauten nicht erreicht?

**16) Impersonal – accidental**

Du bist ein/e Ingenieur/in in der Internationalen Raumstation ISS. Plötzlich fängt der Laderaum Feuer. Das automatische Sicherheitssystem kann den Sauerstoff ablassen und so das Feuer löschen. Allerdings wird dafür die Außentür geöffnet und das funktioniert nur wenn die Innenluke geschlossen ist. Hinter der Luke befindet sich aber noch ein Mechaniker. Du siehst, dass ihm nicht die Zeit bleibt, um die schwere Ausrüstung auszuziehen und dass er steckenbleiben wird, wenn er versucht durch die Innenluke zu kommen. Das wird dazu führen, dass sich das Feuer ausbreitet und euch alle tötet.

Wenn du einen Schalter betätigst, bevor der Mechaniker die Innenluke erreicht, wird sich die Innenluke schließen, was das Notfallsystem aktiviert und das Feuer löscht. Dies wird den Mechaniker in den Weltraum schleudern und ihn töten, aber es wird dich und die verbleibenden zehn Astronauten retten.

Löschst du das Feuer, indem du den Schalter betätigst, der die Innenluke schließt, was den Mechaniker in den Weltraum schleudern wird, damit das Feuer dich und die zehn Astronauten nicht erreicht?

**17) Personal – instrumental**

Du bist ein/e Inspektor/in in einem Atomkraftwerk. Der Direktor und du besichtigt es gerade, als eine der Zuleitungen sich überhitzt. Das Not-Kühlsystem versagt und eine Kettenreaktion steht bevor, die zu einer Kernschmelze führen wird. Wenn dies passiert, wird die freigesetzte Strahlung tödlich für dich und die Bewohner der nahe gelegenen Stadt sein. Tausende Menschen werden sterben.

Wenn du den Direktor von der Brücke schubst und er auf die Zuleitungen fällt, wird genügend Hitze freigesetzt, um die Kettenreaktion aufzuhalten. Dies wird ihn töten, aber es wird dich und die tausenden Einwohner der Stadt retten.

Kühlst du die Leitungen, indem du den Direktor von der Brücke auf die Zuleitungen schubst, damit die für dich und tausende Menschen so gefährliche Kernschmelze verhindert wird?

**18) Impersonal – accidental**

Du bist ein/e Inspektor/in in einem Atomkraftwerk. Der Direktor und du besichtigt es gerade, als eine der Zuleitungen sich überhitzt. Das Not-Kühlsystem versagt und eine Kettenreaktion steht bevor, die zu einer Kernschmelze führen wird. Wenn dies passiert, wird die freigesetzte Strahlung tödlich für dich und die Bewohner der nahe gelegenen Stadt sein. Tausende Menschen werden sterben.

Wenn du flüssigen Stickstoff manuell in die Zuleitungskammer einführst, wird genügend Hitze freigesetzt um die Kettenreaktion aufzuhalten. Dies wird allerdings einen Mitarbeiter töten, der in der Zuleitungskammer festsitzt, aber es wird dich und die tausenden Einwohner der Stadt retten.

Setzt du die Hitze frei, indem du flüssigen Stickstoff in die Zuleitungskammer einführst, in der sich ein Mitarbeiter befindet, damit die für dich und tausende Menschen so gefährliche Kernschmelze verhindert wird?

**19) Personal – instrumental**

Du bist ein/e Sprengstoff-Experte/in eines Unternehmens, das den Auftrag hat ein Hochhaus abzureissen. Du prüfst gerade die letzte Sprengladung, als du einen Jungen siehst, der im Begriff ist versehentlich eine der Sprengladungen zu detonieren. Die Explosion würde zu einem unkontrollierten Einsturz des Gebäudes führen und dich, den Jungen und eine Menge Zuschauer unter sich begraben. Der Junge befindet sich mehrere Stockwerke unter dir und kann dich nicht hören.

Wenn du einen schweren Betonblock auf seinen Kopf fallen lässt, wird es ihn töten, aber es wird dich und die Menschenmenge, retten indem es die Explosion verhindert.

Hälst du den Jungen auf, indem du den Block auf seinen Kopf fallen lässt, damit das Gebäude nicht über dir und der Zuschauermenge einstürzt?

**20) Impersonal-Instrumental**

Du bist ein/e Sprengstoff-Experte/in eines Unternehmens, das den Auftrag hat ein Hochhaus abzureissen. Du prüfst gerade die letzte Sprengladung, als du einen Jungen siehst, der im Begriff ist versehentlich eine der Sprengladungen zu detonieren. Die Explosion würde zu einem unkontrollierten Einsturz des Gebäudes führen und dich, den Jungen und eine Menge Zuschauer unter sich begraben. Der Junge befindet sich mehrere Stockwerke unter dir und kann dich nicht hören.

Wenn du den Strom des Gebäudes wieder anstellst wird der Junge an dem Stromschlag sterben, da er gerade einen offenen Stromkreis berührt. Dies wird ihn töten, aber es wird dich und die Menschenmenge retten, indem es die Explosion verhindert.

Hälst du den Jungen auf, indem du den Strom des Gebäudes wieder anstellst, damit das Gebäude nicht über dir und der Zuschauermenge einstürzt?

21) **Personal – instrumental**

Du bist ein Bauarbeiter. Du und dein Team arbeitet auf einem Gerüst an einem Hochhaus. Plötzlich stürzt das Gerüst teilweise ein. Du und fünf Kollegen hängt an einer Querstange, aber die Stange hält euer Gewicht nicht länger aus. Ein Kollege rutscht von der Stange und klammert sich an deinem Arm fest. Das Gerüst ist im Begriff einzustürzen.

Wenn du den Kollegen wiederholt schlägst, damit er dich loslässt und herunterfällt, reduzierst du ausreichend Gewicht, damit das Gerüst nicht einstürzt. Der Sturz wird den Kollegen töten, aber es wird dich und die anderen fünf Kollegen retten.

Reduzierst du Gewicht, indem du diesen Kollegen schlägst bis er loslässt, damit das Gerüst nicht über dir und den anderen fünf Kollegen einstürzt?

**22) Impersonal-Instrumental**

Du bist ein Bauarbeiter. Du und dein Team arbeitet auf einem Gerüst an einem Hochhaus. Plötzlich stürzt das Gerüst teilweise ein. Du und fünf Kollegen hängt an einer Querstange, aber die Stange hält euer Gewicht nicht länger aus. Ein Kollege rutscht von der Stange und klammert sich an deinem Arm fest. Das Gerüst ist im Begriff einzustürzen.

Wenn du den Stift entfernst, an dem der Teil der Stange befestigt ist, an dem sich der Kollege festhält, wird dieser herunterfallen. Dadurch wird euer Gewicht ausreichend reduziert. Der Sturz wird den Kollegen töten, aber es wird dich und die anderen fünf Kollegen retten.

Reduzierst du das nötige Gewicht, indem du den Stift entfernst, damit du und die anderen fünf Kollegen weiter an der Stange hängen könnt?

**23) Personal – accidental**

Du bist Busfahrer/in und fährst den Bus auf einem Kindergartenausflug auf dem Weg in den Zoo. Auf dem Weg stürzt ihr wegen eines Reifenversagens von einer Brücke in einen wilden Fluss. Du und drei Kinder befindet euch noch im sinkenden Fahrzeug. Die anderen sind schon vom Fluss mitgerissen worden. Du nimmst die beiden Kinder an deiner Seite und schwimmst mit ihnen zum Ausgang. Das dritte Kind klammert sich an deinem Bein fest. Du merkst, dass du nicht stark genug bist, um mit allen drei Kindern gegen die Strömung anzuschwimmen.

Wenn du das Kind abschüttelst, das sich an deinem Bein festklammert, wirst du gegen die Strömung ankämpfen können, aber das Kind wird ertrinken. Das wird es töten, aber es wird dich und die anderen beiden Kinder retten.

Kämpfst du gegen die Strömung an, indem du das Kind abschüttelst, was dazu führen wird dass es ertrinkt, damit du und die anderen beiden Kinder die Oberfläche erreichen könnt?

**24) Impersonal-accidental**

Du bist Busfahrer/in und fährst den Bus auf einem Kindergartenausflug auf dem Weg in den Zoo. Auf dem Weg stürzt ihr wegen eines Reifenversasgen von einer Brücke in einen wilden Fluss. Du und drei Kinder befindet euch noch im sinkenden Fahrzeug. Die anderen sind schon vom Fluss mitgerissen worden. Du nimmst die beiden Kinder an deiner Seite und schwimmst mit ihnen zum Ausgang, doch dieser ist durch einige zerstörte Sitze blockiert.

Wenn du die Sitze entfernst, wirst du den Ausgang freimachen, aber du wirst den Gang blockieren und das dritte Kind wird dort eingesperrt ertrinken. Das wird es töten, aber es wird dich und die anderen beiden Kinder retten.

Machst du den Ausgang frei, indem du die Sitze in den Gang schiebst, was dazu führt, dass das dritte Kind eingesperrt wird, damit du und die anderen beiden Kinder die Oberfläche erreichen könnt?

**(25) Personal – instrumental**

Du bist Arzt/Ärztin in einem Krankenhaus. Du hast fünf Patienten die sterben werden, wenn sie nicht sofort eine Organspende bekommen. Ein weiterer Patient wird gerade nach einem schweren Autounfall eingeliefert. Sein Zustand ist kritisch, aber wahrscheinlich kannst du ihn durch eine lange und komplizierte Operation retten.

Wenn du während der Operation des Unfallopfers unauffällig seine Halsschlagader durchschneidest, verursachst du seinen Tod und wirst so die Organe für die anderen fünf Patienten zur Verfügung haben. Dies wird das Unfallopfer töten, aber es wird die anderen fünf Patienten retten.

Beschafftst du dir die Organe, indem du die Halsschlagader des Unfallopfers durchtrennst, damit du sie bei den anderen fünf Patienten verwenden kannst?

**26) Impersonal-Instrumental**

Du bist Arzt/Ärztin in einem Krankenhaus. Du hast fünf Patienten die sterben werden wenn sie nicht sofort eine Organtransspende bekommen. Ein weiterer Patient wird gerade nach einem schweren Autounfall eingeliefert. Während der langen und komplizierten Operation siehst du wie eine Krankenschwester im Begriff ist eine falsche Injektion zu verabreichen, die in diesem Fall tödlich wäre.

Wenn du die Krankenschwester die falsche Injektion verabreichen lässt wird das Unfallopfer sterben und du wirst die Organe für die anderen fünf Patiente zur Verfügung haben. Dies wird das Unfallopfer töten, aber es wird die anderen fünf Patienten retten.

Beschafftst du dir die Organe, indem du die Krankenschwester dem Unfallopfer die falsche Injektion verabreichen lässt, damit du sie bei den anderen fünf Patienten verwenden kannst?

**27) Personal – accidental**

Du gehörst zu einem Hafenarbeiterteam, das Kranketten an Containern auf Frachtschiffen festmacht, um diese zu entladen. Du und die anderen befestigt gerade Ketten an einem Container und steigt auf diesen herauf, um sicherzustellen, dass er ordnungsgemäß entladen wird. Plötzlich geht das rote Licht an, welches anzeigt, dass die Ketten versagen werden. Du siehst, dass der Grund dafür zwei streitende Kollegen sind. Der Container schwingt gefährlich über den Köpfen von fünf Kollegen, die unten auf dem Deck stehen, hin und her.

Wenn du die zwei Streitenden schubst und sie trennst, wirst du verhindern, dass der Container herunterfällt. Allerdings hat einer der zwei seinen Sicherheitsgurt nicht angelegt und wird herunterfallen. Das wird ihn töten, aber es wird die fünf Kollegen, die auf dem Deck stehen, retten.

Stoppst du das Schwingen des Containers, indem du deine Kollegen schubst, was dazu führen wird, dass der ohne Sicherheitsgurt herunterfallen wird, damit der Container nicht auf die fünf Kollegen, die auf dem Deck stehen stürzt?

**28) Impersonal – accidental**

Du gehörst zu einem Hafenarbeiterteam, das Kranketten an Containern auf Frachtschiffen festmacht, um diese zu entladen. Du und die anderen befestigt gerade Ketten an einem Container und steigt auf diesen herauf, um sicherzustellen, dass er ordnungsgemäß entladen wird. Plötzlich geht das rote Licht an, welches anzeigt, dass die Ketten versagen werden. Wenn sie über dem Deck reißen, wird der Container auf fünf Kollegen stürzen, die dort arbeiten.

Wenn du einen Notfallschalter betätigst, wird der Container zurück in den Laderaum fallen gelassen. Ihr werdet alle von euren Sicherheitsgurten aufgefangen werden, aber ein Kollege befindet sich noch im Laderaum. Der herabstürzende Container wird ihn töten, aber es wird die fünf Kollegen an Deck retten.

Lässt du den Container zurück in den Laderaum fallen, indem du den Notfallschalter betätigst, was dazu führt, dass er auf den Kollegen stürzt, damit er nicht auf die fünf Kollegen an Deck stürzt?

**29) Personal – accidental**

Eine Epidemie hat sich auf dem ganzen Planeten ausgebreitet und schon tausende Menschen getötet. Du bist Forscher/in und hast zwei Substanzen im Labor entwickelt. Durch einen Fehler sind beide als Impfstoff etikettiert. Eine davon ist tatsächlich der Impfstoff der tausende Leben retten könnte, die andere ist eine tödliche Substanz.

Wenn du deinen zwei Laborassistenten je eine dieser beiden Substanzen injizierst, wirst du den Impfstoff identifizieren können. Einer der beiden Assistenten wird sterben, aber du wirst mit dem Impfstoff tausende Leben retten können.

Identifizierst du den Impfstoff, indem du die Substanzen deinen Assistenten injizierst, was dazu führen wird, dass einer der beiden vergiftet wird, damit tausende Menschen geimpft werden können?

**30) Impersonal-accidental**

Eine Epidemie hat sich auf dem ganzen Planeten ausgebreitet und schon tausende Menschen getötet. Du bist Forscher/in und hast zwei Substanzen im Labor entwickelt. Durch einen Fehler sind beide als Impfstoff etikettiert. Eine davon ist tatsächlich der Impfstoff der tausende Leben retten könnte, die andere ist eine tödliche Substanz.

Wenn du einem Krankenpfleger jeweils eine dieser beiden Substanzen zwei Patienten injizieren lässt, wirst du den Impfstoff identifizieren können. Einer der beiden Patienten wird sterben, aber du wirst tausende Leben mit dem Impfstoff retten.

Identifizierst du den Impfstoff indem du dem Krankenpfleger die Substanzen zwei Patienten injizieren lässt, was dazu führen wird, dass einer der beiden vergiftet wird, damit tausende Menschen geimpft werden können?

**31) Personal – instrumental**

Eine Straßenbahn außer Kontrolle steuert mit hoher Geschwindigkeit auf fünf Arbeiter zu. Diese werden sterben, wenn die Bahn nicht aufgehalten wird. Du stehst an den Gleisen, bist aber zu weit weg, um sie zu warnen. Neben dir steht ein unbekannter, sehr fülliger Mann.

Wenn du den Unbekannten auf die Gleise stößt, wird sein Körper die Straßenbahn zum entgleisen bringen und die fünf Arbeiter werden nicht überfahren. Dies wird den Unbekannten töten, aber du wirst die fünf Arbeiter retten.

Lässt du die Straßenbahn entgleisen, indem du den Unbekannten auf die Gleise stößt, damit die Straßenbahn die fünf Arbeiter nicht überfährt?

**32) Impersonal – accidental**

Eine Straßenbahn außer Kontrolle steuert mit hoher Geschwindigkeit auf fünf Arbeiter zu. Diese werden sterben, wenn die Bahn nicht aufgehalten wird. Du stehst an den Gleisen, bist aber zu weit weg, um sie zu warnen Neben dir befindet sich ein Kontrollhebel der die Strassenbahn auf ein anderes Gleis umleiten kann. Hier befindet sich nur ein Arbeiter.

Wenn du den Stellhebel betätigst, wird die Straßenbahn auf das Gleis auf dem ein Arbeiter steht, umgeleitet und wird die anderen fünf Arbeiter nicht überfahren. Das wird diesen einen Arbeiter töten, aber es wird die anderen Fünf retten.

Lenkst du die Straßenbahn um, indem du den Stellhebel betätigst, was dazu führen wird dass ein Arbeiter überfahren wird, damit die Straßenbahn die fünf anderen Arbeiter nicht überfährt?

**33) Personal – instrumental**

Du und ein Forschungskollege habt eine neue Energiequelle entdeckt, die billig, sauber und sicher ist. Sie könnte der Umweltverschmutzung und der Armut in der Welt ein Ende setzen. Allerdings will dein Kollege die Entdeckung verkaufen und du weißt, dass er plant heute mit potenziellen Käufern Kontakt aufzunehmen. Du weißt ebenfalls mit Sicherheit, dass diese die Energie als schreckliche Waffe einsetzen wollen, aber dein Kollege glaubt dir nicht.

Wenn du deinen Kollegen mit einem gänigen Gift aus eurem Labor vergiftest, wirst du verhindern, dass die Entdeckung in falsche Hände gerät. Das Gift wird deinen Kollegen töten, aber du wirst Tausende Menschen retten.

Verhinderst du den Verkauf der Entdeckung, indem du deinen Kollegen vergiftest, damit die Energiequelle nicht als Waffe gegen tausende Menschen eingesetzt werden kann?

**34) Impersonal-Instrumental**

Du und ein Forschungskollege habt eine neue Energiequelle entdeckt, die billig, sauber und sicher ist. Sie könnte der Umweltverschmutzung und der Armut in der Welt ein Ende setzen. Allerdings will dein Kollege die Entdeckung verkaufen und du weißt, dass er plant heute mit potenziellen Käufern Kontakt aufzunehmen. Du weißt ebenfalls mit Sicherheit, dass diese die Energie als schreckliche Waffe einsetzen wollen, aber dein Kollege glaubt dir nicht.

Wenn du ein entflammbares Gas im Labor freisetzt, wird es eine Explosion geben, in dem Moment, wenn dein Kollege den Computer anmacht. Dies wird ihn töten, aber du wirst tausende Menschen retten.

Lässt du den Computer deines Kollegen explodieren, indem du das entflammbare Gas freisetzt, um zu verhindern, dass die Entdeckung als Waffe gegen tausende Menschen eingesetzt wird?

**35) Personal – instrumental**

Du bist ein/e erfahrene/r Motorradfahrer/in der/die an einem Motorradevent teilnimmt. Du fährst vor einer großen Gruppe anderer Motorradfahrer. Plötzlich siehst du, wie ein Fahrer weiter vorne die Kontrolle über sein Motorrad verliert. Während du beschleunigst, um ihm zur Hilfe zu eilen, merkst du, dass er jeden Moment stürzen wird. Dies wird eine Massenkarambolage auslösen und eine Gruppe von zehn Fahrern hinter dir töten.

Wenn du den stürzenden Motorradfahrer von der Straße abdrängst wird er gegen die Bäume fahren, aber du wirst die Massenkarambolage verhindern. Bei eurer Geschwindigkeit wird ihn das töten, aber du wirst die Gruppe der zehn Fahrer hinter Dir retten.

Verhinderst du die Massenkarambolage, indem du den Motorradfahrer von der Straße abkommen lässt, um zu vermeiden, dass die anderen zehn Fahrer stürzen?

**36) Impersonal-instrumental**

Du bist ein/e erfahrene/r Motorradfahrer/in der/die an einem Motorradevent teilnimmt. Du fährst vor einer großen Gruppe anderer Motorradfahrer. Plötzlich siehst du, wie ein Fahrer weiter vorne die Kontrolle über sein Motorrad verliert und stürzt. Du selbst kannst ihn leicht umfahren, aber zehn Motorradfahrer hinter dir werden im Versuch ihm auszuweichen aufeinander auffahren, was in einer tödlichen Massenkarambolage enden wird.

Wenn du dein Motorrad gegen das des stürzenden Motorradfahrers fahren lässt, während du geschickt in den Straßengraben springst, wirst dies die anderen rechtzeitig alarmieren. Dies wird den stürzenden Motorradfahrer töten, aber du wirst die anderen zehn Motorradfahrer retten.

Machst du die anderen auf die Unfallgefahr aufmerksam, indem du dein Motorrad gegen das des stürzenden Motorradfahrers fahren lässt, damit die zehn Motorradfahrer hinter dir genug Zeit haben ihm auszuweichen?

**37) Personal – instrumental**

Du bist der/die Anführer/in einer kleinen Gruppe von Soldaten. Ihr seid auf dem Rückweg von einer Mission in feindlichem Gebiet, als einer deiner Männer in eine Falle tritt, die sein Bein erfasst und es schwer verletzt. Du kannst ihn nicht befreien, ohne ihn zu töten, aber wenn du ihn zurücklässt werden die feindlichen Truppen ihn finden und foltern, bis er den Standort eines wichtigen Basecamps eurer Alliierten verrät. Danach werden eure Feinde dieses Lager angreifen und die 10 Soldaten der sich dort aufhaltenden Truppe töten. Der Soldat fleht dich an, ihn nicht zurückzulassen, aber die feindlichen Truppen nähern sich und es ist gefährlich für dich und deine Männer länger zu bleiben, da ihr keine Munition mehr habt.

Wenn du den Soldaten in der Falle erstichst, verhinderst du, dass er gefoltert wird und die Information preisgibt, die zum Tod der Soldaten der alliierten Truppe führen wird. Dies wird ihn töten, aber du wirst zehn alliierete Truppenmitglieder retten.

Verhinderst du, dass der Soldat in der Falle gefoltert wird, indem du ihn selbst erstichst, damit er nicht den Aufenthaltsort der zehn alliierten Truppenmitglieder preisgeben kann?

**38) Impersonal – Instrumental**

Du bist der/die Anführer/in einer kleinen Gruppe von Soldaten. Ihr seid auf dem Rückweg von einer Mission in feindlichem Gebiet, als einer deiner Männer in eine Falle tritt, die sein Bein erfasst und es schwer verletzt. Du kannst ihn nicht befreien, ohne ihn zu töten, aber wenn du ihn zurücklässt werden die feindlichen Truppen ihn finden und foltern, bis er den Standort eines wichtigen Basecamps eurer Alliierten verrät. Danach werden eure Feinde dieses Lager angreifen und die 10 Soldaten der sich dort aufhaltenden Truppe töten. Der Soldat fleht dich an, ihn nicht zurückzulassen, aber die feindlichen Truppen nähern sich und es ist gefährlich für dich und deine Männer länger zu bleiben, da ihr keine Munition mehr habt.

Wenn du die Position des Soldaten in der Falle mit einer Leuchtrakete signalisierst, wird die Gegend bombardiert. So verhinderst du, dass er gefoltert wird und die Information preisgeben kann, die zum Tod der alliierten Truppe führt. Dies wird ihn töten, aber du wirst die zehn Truppenmitglieder retten.

Verhinderst du, dass der Soldat in der Falle gefoltert wird, indem du die Gegend bombardieren lässt, damit er nicht den Aufenthaltsort der zehn alliierten Truppenmitglieder preisgeben kann?

**39) Personal – instrumental**

Du bist der/die Nachtwächter/in in einem Krankenhaus. Ein chemisches Produkt ist in einem Zimmer mit sechs Patienten verschüttet worden. Es ist hochgiftig, denn es verdampft nach kurzer Zeit unter Sauerstoffeinfluss und ein giftiges Gas entsteht was alle Patienten in dem Zimmer töten wird. Man kann es nicht mit herkömlichen Handtüchern oder Lappen wegwischen. Allerdings absorbiert die menschliche Haut es sehr schnell.

Wenn du einen der Patienten auf das Produkt legst, verhinderst du, dass das giftige Gas entsteht. Dieser Patient wird sterben, aber du wirst die anderen fünf Patienten in dem Zimmer retten.

Verhinderst du die Entstehung des giftigen Gases, indem du einen Patienten auf das Produkt legst, damit die anderen fünf Patienten nicht vergiftet werden?

**40) Impersonal – accidental**

Du bist der/die Nachtwächter/in in einem Krankenhaus. Ein chemisches Produkt ist in einem Zimmer mit sechs Patienten verschüttet worden. Es ist hochgiftig, denn es verdampft nach kurzer Zeit unter Sauerstoffeinfluss und ein giftiges Gas entsteht was alle Patienten in dem Zimmer töten wird. Man kann es nicht mit herkömlichen Handtüchern oder Lappen wegwischen.

Wenn du das Ventilationssystem aktivierst, wird das Gas aus dem Zimmer herausgesaugt. Allerdings wird es in einem Raum im oberen Stockwerk gepumpt, wo sich ein anderer Patient befindet, den du nicht schnell genug verlegen kannst. Das wird diesen Patient töten, aber du wirst die anderen fünf retten.

Lässt du das Gas aus dem Zimmer absaugen, indem du das Ventilationssystem aktivierst, was dazu führt, dass es in das Zimmer des einzelnen Patient gepumpt wird, damit die anderen fünf Patienten nicht vergiftet werden?

**41) Personal – instrumental**

Du befindest dich mit einem Reiseleiter in einem von zwei Ruderbooten auf einem See in Alaska. Im anderen Boot sind drei Kinder, aber dieses ist gerade gekentert und die Drei laufen nun Gefahr zu erfrieren. Du ruderst in ihre Richtung aber merkst, dass dein Boot nicht das Gewicht von dir, dem Reiseleiter und das der drei Kinder wird tragen können. Ihr rettet die Kinder, aber eines wiegt sehr viel und ihr werdet untergehen. Weder du noch der Reiseleiter könnt das Boot verlassen, denn du ruderst und der Reiseleiter reanimiert gerade eins der Kinder.

Wenn du das schwerere Kind über Bord wirfst, werdet ihr nicht kentern und zwei der Kinder werden gerettet sein können. Das über Bord geworfene Kind wird sterben, aber du wirst die anderen beiden retten.

Machst du das Boot leichter indem du das schwerere Kind über Bord wirfst, damit ihr mit den anderen beiden Kindern das Ufer erreichen könnt?

**42) Impersonal- accidental**

Du befindest dich mit einem Reiseleiter in einem von zwei Ruderbooten auf einem See in Alaska. Im anderen Boot sind drei Kinder, aber dieses ist gerade gekentert und die Drei laufen nun Gefahr zu erfrieren. Du ruderst in ihre Richtung aber merkst, dass dein Boot nicht das Gewicht von dir, dem Reiseleiter und das der drei Kinder wird tragen können. Ihr rettet die Kinder, aber eines wiegt sehr viel und ihr werdet untergehen. Weder du noch der Reiseleiter könnt das Boot verlassen, denn du ruderst und der Reiseleiter reanimiert gerade eins der Kinder.

Wenn du zum Ufer ruderst und das dritte Kind im Wasser zurücklässt verhinderst du, dass ihr untergeht. Dies wird das zurückgelassene Kind töten, aber du wirst die anderen beiden retten.

Verhinderst du, dass ihr untergeht, indem du das dritte Kind zurücklässt, was dazu führen wird dass es erfriert, damit ihr mit den anderen beiden Kindern das Ufer erreichen könnt?

**43) Personal – instrumental**

Du bist Minenarbeiter im Untertagebau. Nach oben gelangt man nur über einen einfachen Seilaufzug, an dem mehrere Kabinen hängen. Jede Kabine hält das Gewicht von drei Personen. Ihr habt den Arbeitstag beendet und fahrt in den Kabinen nach oben. Während du wartest siehst du plötzlich, dass eins der Kabel an dem alle Kabinen festgemacht sind, gleich reißen wird. 13 Minenarbeiter befinden sich gerade in den verschiedenen Kabinen.

Wenn du die letzte Kabine umstürzt, in der nur ein einziger Minenarbeiter fährt, reduzierst du ausreichend Gewicht damit das Kabel mit den Kabinen der zwölf anderen Minenarbeitern nicht reißt. Der Minenarbeiter in der umgestürzten Kabine stirbt, aber du wirst die anderen zwölf retten.

Reduzierst du das Gewicht, indem du die Kabine des einzelnen Minenarbeiters umstürzt, damit die Kabinen der anderen zwölf Bergarbeiter nicht abstürzten?

**44) Impersonal- instrumental**

Du bist Minenarbeiter im Untertagebau. Nach oben gelangt man nur über einen einfachen Seilaufzug, an dem mehrere Kabinen hängen. Jede Kabine hält das Gewicht von drei Personen. Ihr habt den Arbeitstag beendet und fahrt in den Kabinen nach oben. Während du wartest siehst du plötzlich, dass eins der Kabel an dem alle Kabinen festgemacht sind, gleich reißen wird. 10 Minenarbeiter befinden sich gerade in den verschiedenen Kabinen.

Wenn du den Notfallknof betätigst stürzt die letzte Kabine vom Seilaufzug. In dieser Kabine befindet sich nur ein Minenarbeiter und du würdest so das Gewicht ausreichend reduzieren, um die Kabinen der anderen neun Minenarbeiter anzuhalten. Der eine Minenarbeiter wird bei dem Sturz getötet, aber du wirst die anderen neun retten.

Reduzierst du das Gewicht, indem du den Knopf betätigst, der die Kabine mit dem einen Minenarbeiter umstürzt, damit die Kabinen der anderen neun Bergarbeiter anzuhalten?

**45) Personal – accidental**

Du bist ein/e Elektriker/in, der/die im Haus eines reichen Großindustriellen eine Stromleitung reparieren soll. Der Mann ist unheilbar an Krebs erkrankt und es wurde vor kurzem bekannt, dass nach seinem Tod sein ganzes Vermögen an ein lokales Kinderkrankenhaus gespendet wird. In diesem Krankenhaus befinden sich 109 Kinder, die mit diesem Geld gerettet werden könnten. Sie können aber nicht länger warten, da sie kurz vor dem Tode sind.

Wenn du den Großindustriellen erstickst, indem du, während er schläft, seinen Mund und seine Nase mit deinen behandschuhten Händen zuhälst, wirst du die lebensnotwendige Spende beschleunigen. Dies wird den Großindustriellen töten, aber die Leben der 109 Kinder retten.

Beschleunigst du die Spende, indem du diesen Mann erstickst, damit die Spende rechtzeitig bei den 109 kranken Kindern ankommt?

**46) Impersonal-Instrumental**

Du bist ein/e Elektriker/in, der/die im Haus eines reichen Großindustriellen eine Stromleitung reparieren soll. Der Mann ist unheilbar an Krebs erkrankt und es wurde vor kurzem bekannt, dass nach seinem Tod sein ganzes Vermögen an ein lokales Kinderkrankenhaus gespendet wird. In diesem Krankenhaus befinden sich 112 Kinder, die mit diesem Geld gerettet werden könnten. Sie können aber nicht länger warten, da sie kurz vor dem Tode sind.

Wenn du die Stromkreise kurzschließt, anstatt das Problem zu beheben, wird das Beatmungsgerät des Mannes ausfallen und du wirst die Spende für die Kinder beschleunigen. Dies wird den Großindustriellen töten, aber du wirst die Leben der 112 Kinder retten.

Beschleunigst du die Spende, indem du einen Kurzschluss verursachst, der das Beatmungsgerät des Großindustriellen ausfallen lässt, damit die Spende rechtzeitig bei den 112 kranken Kindern ankommt?

47) **Personal – instrumental**

Du bist ein Militäroffizier im Krieg. Deine Soldaten haben einen wichtigen Spion aufgefunden, der verletzt und festgeklemmt in einem eingestürzten Gebäude liegt. Per Funk bist Du informiert worden, dass feindliche Soldaten einen Überraschungsangriff auf eine nahe gelegene Stadt planen. Du weißt aber nicht auf welche. Tausende Zivilisten werden getötet, wenn du und deine Soldaten sie nicht aufhalten. Der Spion weiß um welche Stadt es sich handelt. Du hast ihn verhört, aber er verrät es dir nicht. Er wird wegen seiner Verletzungen nicht mehr lange leben.

Wenn du den Spion folterst, wird er preisgeben welche Stadt angegriffen werden soll. Die Folter wird ihn töten, aber du wirst tausende Zivilisten retten.

Sorgst du dafür, dass der Spion die Information preisgibt, indem du ihn folterst, damit ihr den Angriff auf tausende Zivilisten aufhalten könnt?

**48) Impersonal-Instrumental**

Du bist ein Militäroffizier im Krieg. Deine Soldaten haben einen wichtigen Spion aufgefunden, der verletzt und festgeklemmt in einem eingestürzten Gebäude liegt. Per Funk bist Du informiert worden, dass feindliche Soldaten einen Überraschungsangriff auf eine nahe gelegene Stadt planen. Du weißt aber nicht auf welche. Tausende Zivilisten werden getötet, wenn du und deine Soldaten sie nicht aufhalten. Der Spion weiß um welche Stadt es sich handelt. Du hast ihn verhört, aber er verrät es dir nicht. Er wird wegen seiner Verletzungen nicht mehr lange leben. Ratten nagen an ihm und er leidet sehr.

Wenn du die Ratten mit dieser Folter weitermachen lässt, wird der Spion preisgeben, welche Stadt angegriffen werden soll. Die Folter wird ihn töten, aber du wirst tausende Zivilisten retten.

Sorgst du dafür, dass der Spion die Information preisgibt, indem du die Ratten weiter an ihm nagen lässt, damit ihr den Angriff auf tausende Zivilisten aufhalten könnt?

**49) Personal – Others Beneficial – Inevitable (instrumental)**

Du bist Arzt/Ärztin. Einer deiner Patienten, der mit AIDS infiziert ist, bekommt gerade eine Bluttransfusion, bevor er aus dem Krankenhaus entlassen wird. Er gesteht dir, dass er beabsichtigt, so viele Menschen wie möglich zu infizieren und dass er an diesem Nachmittag, nach dem Verlassen des Krankenhauses, damit beginnen wird. Du bist durch die ärztliche Schweigepflicht gebunden und hast keine rechtliche Grundlage, um ihn aufzuhalten, denn die Polizei kann ihn nicht auf der Grundlage von vertraulichen Informationen verhaften.

Wenn du seine Medikamente mit einem nicht nachweisbaren Gift verunreinigst, wirst du verhindern, dass er andere ansteckt. Dies wird ihn töten, aber du wirst viele retten.

Verhinderst du, dass viele Menschen durch deinen Patienten mit AIDS infiziert werden, indem du seine Medizin verunreinigst, damit er nicht andere Personen anstecken kann?

**50) Impersonal – Others Beneficial – Inevitable (instrumental)**

Du bist Arzt/Ärztin. Einer deiner Patienten, der mit AIDS infiziert ist, bekommt gerade eine Bluttransfusion, bevor er aus dem Krankenhaus entlassen wird. Er gesteht dir, dass er beabsichtigt, so viele Menschen wie möglich zu infizieren und dass er an diesem Nachmittag, nach dem Verlassen des Krankenhauses, damit beginnen wird. Du bist durch die ärztliche Schweigepflicht gebunden und hast keine rechtliche Grundlage, um ihn aufzuhalten, denn die Polizei kann ihn nicht auf der Grundlage von vertraulichen Informationen verhaften

Wenn du seine Blutgruppe in der Datenbank des Krankenhauses änderst, wird er vom falschen Blut vergiftet und du wirst verhindern, dass er viele Menschen anstecken kann. Dies wird ihn töten, aber du wirst viele retten.

Verhinderst du, dass viele Menschen durch deinen Patienten mit AIDS infiziert werden, indem du die Blutgruppe des Patienten änderst, damit er nicht andere Personen anstecken kann?

**51) Personal – Others Beneficial – Avoidable (instrumental)**

Du verhandelst mit einem psychisch kranken, bewaffneten Mann auf dem Dach eines Gebäudes. Unten befindet sich eine Menschenmenge auf dem Kunst-und Musikfestival der Stadt. Er sagt, dass er auf die Menschenmenge schießen will und du schaffst es nicht ihn davon abzubringen. Die Menge weiß nichts von der Bedrohung und du hast keine Möglichkeit die Menschen zu warnen. Von seiner Position aus könnte er ein dutzend Menschen töten, ohne diejenigen mitzuzählen, die durch das entstehende Chaos verletzt oder getötet werden würden.

Wenn du losrennst und ihn schubst, wird er vom Dach stürzen und wird nicht auf die Menschenmenge schießen können. Dies wird ihn töten, aber du wirst viele Leben retten.

Verhinderst du die Schießerei und das darauffolgende Chaos, indem du den Mann vom Dach herunterschubst, damit er nicht auf die Menschenmenge schießt?

**52) Impersonal – Others Beneficial – Avoidable (instrumental)**

Du verhandelst mit einem psychisch kranken, bewaffneten Mann auf dem Dach eines Gebäudes. Unten befindet sich eine Menschenmenge auf dem Kunst-und Musikfestival der Stadt. Er sagt, dass er auf die Menschenmenge schießen will und du schaffst es nicht ihn davon abzubringen. Die Menge weiß nichts von der Bedrohung und du hast keine Möglichkeit die Menschen zu warnen. Von seiner Position aus könnte er ein dutzend Menschen töten, ohne diejenigen mitzuzählen, die durch das entstehende Chaos verletzt oder getötet werden würden.

Wenn du die Polizei Bescheid gibst, dass die Verhandlung gescheitert ist, werden sie auf ihn schießen und so wird die Schießerei auf die Menschenmenge verhindert. Dies wird ihn töten, aber du wirst viele Leben retten.

Verhinderst du das Chaos, indem du die Polizei auf den Mann schießen lässt, damit er nicht in die Menschenmenge schießt?
