# Supplementary material for: Moral judgment reloaded: a moral dilemma validation study
Source: Front Psychol. 2014 Jul 1;5:607. doi: 10.3389/fpsyg.2014.00607 (PMC4077230; doi:10.3389/fpsyg.2014.00607)
Supplement: Supplementary file 10 [file DataSheet10.DOC]

**Spanish**

**DILEMAS**

**1) Personal - Instrumental**

Tú y cinco personas más estáis atrapadas en un edificio en llamas. Hay una única salida de emergencia a través de la cuál todos podríais escapar, pero está bloqueada por escombros ardientes. Otra persona herida intenta arrastrarse por un hueco que queda en la base de dicha salida. Tú y las otras cinco personas que tienes detrás no tenéis tiempo de hacer lo mismo.

Si usas la persona herida para desbloquear el paso a través de los escombros podréis escapar. Esto seguro que la matará, pero os salvaréis tú y las cinco personas que tienes detrás.

¿Desbloqueas el paso usando a la persona herida, para que podáis escapar tú y las otras cinco personas?

**2) Impersonal- Accidental**

Tú y cinco personas más estáis atrapadas en un edificio en llamas. Hay una única salida de emergencia a través de la cuál todos podríais escapar, pero está bloqueada por escombros ardientes. Otra persona herida intenta arrastrarse por un hueco que queda en la base de dicha salida. Tú y las otras cinco personas que tienes detrás no tenéis tiempo de hacer lo mismo.

Si activas el sistema de emergencia se eliminará el oxígeno del pasillo, apagando el fuego, pero dejando al herido sin aire. Esto seguro que lo matará, pero os salvaréis tú y las cinco personas que tienes detrás.

¿Apagas el fuego activando el sistema de emergencia, cosa que dejará al herido sin aire, para que tú y las otras cinco personas podáis escapar?

**3) Personal – accidental**

Soldados enemigos han ocupado tu pueblo y matarán a todos los civiles mayores de dos años. Tú y diez vecinos os refugiáis en dos habitaciones del sótano de una gran casa. Oís voces de soldados que han entrado a buscar cosas de valor. Tu bebé empieza a llorar fuertemente. Su llanto llamará la atención de los soldados, que le perdonarán la vida, pero os matarán a ti y a los otros diez refugiados.

Si le tapas la boca con la mano, amortiguarás el llanto, pero el bebé se quedará sin aire. Esto lo matará, pero os salvará a ti y a los diez vecinos.

¿Amortiguas el llanto manteniendo la mano en la boca de tu bebé, para que los soldados no os encuentren a ti y a los diez vecinos?

**4) Impersonal- accidental**

Soldados enemigos han ocupado tu pueblo y matarán a todos los civiles mayores de dos años. Tú y diez vecinos os refugiáis en dos habitaciones del sótano de una gran casa. Oís voces de soldados que han entrado a buscar cosas de valor. Tu bebé empieza a llorar fuertemente. Su llanto llamará la atención de los soldados, que perdonarán la vida de tu bebé, pero os matarán a ti y a los otros diez refugiados.

Si activas una estruendosa caldera que hay se producirá un incómodo calor para los adultos, que será mortal para tu bebé, pero amortiguará su llanto, cosa que os salvará a ti y a los diez vecinos.

¿Amortiguas el llanto de tu bebé, activando la caldera que le asfixiará, para que no os encuentren a ti y a los diez vecinos?

**5) Personal – accidental**

Eres parte de la tripulación de un submarino que navega debajo de un gran iceberg. Una explosión ha dañado la nave, hiriendo a varios compañeros y colapsando el único acceso entre las secciones superior e inferior. Tú y otros diez supervivientes estáis en la sección superior, que no tiene suficiente oxígeno para manteneros con vida hasta salir a la superficie. En la sección inferior, donde hay suficiente oxígeno, yace inconsciente un único compañero.

Si empujas la compuerta de emergencia entre ambas secciones abrirás el paso del aire. Sin embargo, la compuerta caerá sobre el compañero de abajo, matándolo, pero esto os salvará a ti y a los otros diez supervivientes.

¿Abres el paso del aire empujando la compuerta que caerá sobre el compañero, para que tú y los otros diez supervivientes tengáis suficiente oxígeno?

**6) Impersonal- accidental**

Eres parte de la tripulación de un submarino que navega debajo de un gran iceberg. Una explosión ha dañado la nave, hiriendo a varios compañeros y colapsando el único acceso entre las partes superior e inferior. Tú y otros diez supervivientes estáis en la sección superior, que no tiene suficiente oxígeno para manteneros con vida hasta salir a la superficie. En la sección inferior, donde hay suficiente oxígeno, yace inconsciente un único compañero.

Si pulsas un interruptor de emergencia se abrirá una compuerta entre ambas secciones, dejando pasar el aire. Sin embargo, la puerta caerá sobre el compañero, matándolo, pero esto os salvará a ti y a los otros diez supervivientes

¿Abres el paso del aire pulsando el interruptor, cosa que hará caer la compuerta sobre el compañero, para que tú y los otros diez supervivientes tengáis suficiente oxígeno?

**7) Personal** – **instrumental**

Tú y diez submarinistas formáis parte de un equipo de las Naciones Unidas que desactiva minas submarinas de la IIª Guerra Mundial. Un miembro de tu equipo se ha herido y la sangre ha atraído a varios tiburones. Tienes un fusil submarino, pero sólo un arpón, y hay muchos tiburones. El submarinista que sangra está nadando hacia la última jaula protectora y la alcanzará antes que tú y los demás. Los tiburones, siguiendo la sangre, se acercan demasiado como para que tú y los otros submarinistas podáis escapar.

Si disparas al submarinista herido, esto lo matará y los tiburones se detendrán a comérselo, pero tú y los otros submarinistas podréis salvaros.

¿Haces que los tiburones se coman al submarinista herido disparándole, para que tú y los otros nueve submarinistas podáis alcanzar la jaula protectora?

**8) Impersonal-Instrumental**

Tú y diez submarinistas formáis parte de un equipo de de las Naciones Unidas que desactiva minas submarinas de la IIª Guerra Mundial. Un miembro de tu equipo se ha herido y la sangre ha atraído a varios tiburones. Tienes un fusil submarino, pero sólo un arpón, y hay muchos tiburones. El submarinista que sangra está nadando hacia la última jaula protectora y la alcanzará antes que tú y los demás. Los tiburones, siguiendo la sangre, se acercan demasiado como para que tú y los otros submarinistas podáis escapar.

Si detonas por control remoto una de las minas, esto lo matará y los tiburones se pararán a comérselo, pero podréis salvaros tú y los otros submarinistas.

¿Haces que los tiburones se coman al submarinista herido detonando por control remoto la mina próxima a ese submarinista, para que tú y los otros nueve submarinistas podáis alcanzar la jaula protectora?

**9) Personal – instrumental**

Trabajas en un orfanato de un país en guerra que acoge diez niños. Los rebeldes han tomado el gobierno y están matando a todos los que no apoyan a su régimen y además llevan a cabo una campaña genocida contra un determinado grupo étnico. Tú y los diez huérfanos no apoyáis la rebelión y además uno de ellos pertenece al grupo étnico perseguido. No hay forma de que tú y los huérfanos podáis escapar y seguro que los soldados os matarán a todos.

Si asfixias con una almohada al niño del grupo étnico perseguido mientras duerme, los soldados creerán que apoyas su régimen. Esto lo matará, pero os salvará a ti a y a los otros nueve niños.

¿Haces que los soldados crean que apoyas su régimen asfixiando a dicho niño, para salvar la vida de los otros nueve niños y la tuya?

**10) Impersonal – Instrumental**

Trabajas en un orfanato de un país en guerra que acoge diez huérfanos. Los rebeldes han tomado el gobierno y están matando a todos los que no apoyan su régimen y además llevan a cabo una campaña genocida contra un determinado grupo étnico. Tú y los diez huérfanos del orfanato no los apoyáis y además uno de los niños pertenece al grupo étnico perseguido. No hay forma de que tú y los huérfanos podáis escapar y seguro que los soldados os matarán a todos.

Si ordenas a la enfermera que le dé al niño del grupo étnico perseguido una sobredosis letal de píldoras para dormir, los soldados creerán que apoyas su régimen. Esto lo matará, pero os salvará a ti a y a los nueve niños restantes.

¿Haces que los soldados crean que apoyas su régimen ordenando a la enfermera que dé una sobredosis a dicho niño, para que os perdonen la vida a ti y a los otros nueve niños?

**11) Personal – instrumental**

Eres trabajador/a de la salud voluntario/a en un pueblo rural africano. Un hombre de un pueblo vecino ha contraído el virus del Ébola, que es extremadamente contagioso, incurable y casi siempre mortal. Milagrosamente, ha sobrevivido un mes, por lo que debe ser inmune a los efectos mortales del virus. Sin embargo, él cree erróneamente que tu centro de salud puede curarlo por completo. Lo ves aproximándose y sabes que si entra en el pueblo contagiará a los 119 habitantes inocentes que, a diferencia de él, morirán.

Si le disparas con la pistola del centro prevendrás que os contagie el virus a ti y al resto del pueblo. Esto lo matará, pero te salvará a ti y a los 119 habitantes del pueblo.

¿Impides que el hombre entre en el pueblo, disparándole para que no os contagie a ti y a los 119 habitantes del pueblo?

**12) Impersonal-Instrumental**

Eres trabajador/a de la salud voluntario/a en un pueblo rural africano. Un hombre de un pueblo vecino ha contraído el virus del Ébola, que es extremadamente contagioso, incurable y casi siempre mortal. Milagrosamente, ha sobrevivido un mes, por lo que debe ser inmune a los efectos mortales del virus. Sin embargo, él cree erróneamente que tu centro de salud puede curarlo por completo. Lo ves aproximándose y sabes que si entra en el pueblo contagiará a los 119 habitantes inocentes que, a diferencia de él, morirán.

Si avisas al consejo del pueblo le dispararán, para prevenir que os contagie el virus a ti y al resto del pueblo. Esto lo matará, pero te salvará a ti y a los 119 habitantes del pueblo.

¿Impides que el hombre entre en el pueblo avisando al consejo de que le dispare, para que no os contagie a ti y a los 119 habitantes?

**13) Personal – instrumental**

Eres el/la único/a médico/a de un helicóptero de rescate en una inundación devastadora. Has rescatado a nueve personas heridas en estado crítico y las mantienes vivas. De pronto, el piloto te notifica que hay un fallo en el motor y que hay que reducir carga o el helicóptero se estrellará y todos moriréis. Tú no puedes abandonar el helicóptero porque los heridos necesitan tus cuidados y no puedes lanzar el pesado equipo médico porque está atornillado.

Si lanzas a uno de los heridos, liberarás carga suficiente para mantener el helicóptero en el aire. Esto lo matará, pero os salvará a ti, al piloto, y a los otros ocho heridos.

¿Reduces carga lanzando a uno de los heridos, para mantener el helicóptero en el aire, y salvar las otras diez personas a bordo?

**14) Impersonal-Instrumental**

Eres el/la único/a médico/a de un helicóptero de rescate en una inundación devastadora. Has rescatado nueve personas heridas estado crítico y las mantienes vivas. De pronto, el piloto te notifica que hay un fallo en el motor y que hay que reducir carga o el helicóptero se estrellará y todos moriréis. Tú no puedes abandonar el helicóptero porque los heridos necesitan tus cuidados, ni lanzar el pesado equipo médico porque está atornillado.

Si cortas el cable por el que asciende la última persona rescatada, reducirás peso suficiente para mantener el helicóptero en el aire. Esto lo matará, pero os salvará a ti, al piloto y a los otros ocho heridos a bordo.

¿Liberas peso cortando el cable por el que asciende el último de los heridos, para mantener el helicóptero en el aire y salvar las otras diez personas a bordo?

**15) Personal – accidental**

Eres ingeniero/a en la Estación Espacial Internacional que orbita la Tierra. De pronto se enciende la bodega de carga. El sistema anti-incendios automático funciona abriendo la compuerta exterior de la bodega para que el oxígeno salga y así apagar el fuego, pero sólo funciona con la escotilla interior sellada. El problema es que hay un mecánico en la bodega. Tú ves que no tiene tiempo de quitarse el pesado traje y quedará atrapado si intenta entrar por la escotilla interior, causando que el fuego se extienda y os mate a todos.

Si empujas al mecánico de vuelta a la bodega podrás sellar la escotilla interior, y así se activará el sistema anti-incendios. Esto enviará el mecánico al espacio, matándolo, pero os salvará a ti y a los nueve astronautas restantes.

¿Apagas el fuego sellando la escotilla a mano, cosa que enviará al mecánico al espacio, para que el fuego no os alcance a ti y a los restantes nueve astronautas?

**16) Impersonal – accidental**

Eres ingeniero/a en la Estación Espacial Internacional que orbita la Tierra. De pronto se enciende la bodega de carga. El sistema anti-incendios automático funciona abriendo la compuerta exterior de la bodega para que el oxígeno salga y así apagar el fuego, pero sólo funciona con la escotilla interior sellada. El problema es que hay un mecánico en la bodega. Tú ves que no tiene tiempo de quitarse el pesado traje y quedará atrapado si intenta entrar por la escotilla interior, causando que el fuego se extienda y os mate a todos.

Si pulsas el interruptor que sella la escotilla interior antes de que el mecánico la alcance, se activará el sistema anti-incendios. Esto enviará al mecánico al espacio, matándolo, pero os salvará a ti y a los nueve astronautas restantes.

¿Apagas el fuego pulsando el interruptor que sella la escotilla, cosa que enviará el mecánico al espacio, para que el fuego no os alcance a ti y a los otros nueve astronautas?

**17) Personal – instrumental**

Eres inspector/a en una planta de energía nuclear. El director y tú estáis visitándola cuando uno de los conductos de abastecimiento se sobrecalienta. El sistema refrigerador de emergencia falla y está a punto de empezar una reacción en cadena que resultará en fusión nuclear. La radiación liberada será letal para una ciudad cercana, matando a miles de personas.

Si empujas al director al lugar donde confluyen los conductos de abastecimiento, su cuerpo absorberá suficiente calor para detener la reacción en cadena. Esto lo matará, pero os salvará a ti y a los miles de habitantes de la ciudad.

¿Enfrías los conductos de abastecimiento empujando al director al lugar donde confluyen, para evitar un accidente nuclear que te mataría a ti y a miles de personas?

**18) Impersonal – accidental**

Eres inspector/a en una planta de energía nuclear. El director y tú estáis visitándola cuando uno de los conductos de abastecimiento se sobrecalienta. El sistema refrigerador de emergencia falla y está a punto de empezar una reacción en cadena que resultará en fusión nuclear. La radiación liberada será letal para una ciudad cercana, matando a miles de personas.

Si insertas manualmente nitrógeno líquido en la cámara de abastecimiento se eliminará suficiente calor para detener la reacción en cadena. Esto matará a un empleado atrapado en la cámara, pero os salvará a ti y a los miles de habitantes de la ciudad.

¿Eliminas calor insertando nitrógeno líquido en la cámara de abastecimiento donde se encuentra un empleado, para evitar un accidente nuclear que te mataría a ti y a miles de personas?

**19) Personal – instrumental**

Eres el experto/a en explosivos de una compañía encargada de demoler un rascacielos. Estás examinando la última carga cuando ves a un chico que está a punto de detonar accidentalmente una de las cargas. La explosión resultará en el derrumbe incontrolado del edificio sobre ti, el chico y la multitud de espectadores. El chico está varios pisos por debajo de ti y no puede oírte.

Si dejas caer un pesado bloque de hormigón sobre su cabeza, esto lo matará, pero os salvará a ti y a la multitud porque evitará la explosión.

¿Detienes al chico dejando caer el bloque sobre su cabeza, para que el edificio no se derrumbe sobre ti y la multitud de espectadores?

**20) Impersonal-Instrumental**

Eres el experto/a en explosivos de una compañía encargada de demoler un rascacielos. Estás examinando la última carga cuando ves a un chico que está a punto de detonar accidentalmente una de las cargas. La explosión resultará en el derrumbe descontrolado del edificio sobre ti, el chico y la multitud de espectadores. El chico está varios pisos por debajo de ti y no puede oírte.

Si activas el diferencial devolviendo la electricidad al edificio, el chico se electrocutará dado que está tocando un circuito abierto. Esto lo matará, pero os salvará a ti y a la multitud porque evitará la explosión.

¿Detienes al chico, haciendo que se electrocute mediante la activación del diferencial, para que el edificio no se derrumbe sobre ti y la multitud de espectadores?

21) **Personal – instrumental**

Eres trabajador/a de la construcción. Tú y tu equipo trabajáis sobre un andamio en un rascacielos. De pronto el andamio se derrumba parcialmente. Tú y cinco compañeros estáis suspendidos de un travesaño colgante, pero pesáis demasiado para que resista. Otro compañero patina del travesaño y se agarra de tu brazo. La estructura está a punto de ceder.

Si golpeas repetidamente a ese compañero para que te suelte y caiga, esto restará suficiente peso para mantener el andamio. La caída lo matará, pero os salvará a ti y a los otros cinco compañeros.

¿Liberas peso golpeando a ese compañero, que se caerá, para que tú y los otros cinco compañeros podáis quedar colgando del travesaño?

**22) Impersonal-Instrumental**

Eres trabajador/a de la construcción. Tú y tu equipo trabajáis sobre un andamio en un rascacielos. De pronto el andamio se derrumba parcialmente. Tú y cinco compañeros estáis suspendidos de un travesaño colgante, pero pesáis demasiado para que resista. Otro compañero patina del travesaño y se agarra de otra parte del andamio. La estructura está a punto de ceder.

Si quitas el pasador que sujeta la parte dónde está ese compañero, éste se caerá. Esto restará suficiente peso para mantener el andamio. La caída matará al compañero, pero os salvará a ti y a los otros cinco compañeros.

¿Restas el peso del compañero quitando el pasador, para que tú y los otros cinco compañeros podáis quedar colgando del travesaño?

**23) Personal – accidental**

Eres conductor/a de un autocar de guardería de excursión al zoo. De camino, un defecto en las ruedas provoca que os precipitéis desde un puente a un río bravo. Tú y tres niños estáis todavía en el vehículo que se hunde. El resto han sido arrastrados por el río. Coges a los dos niños que tienes al lado y nadas hacia la salida, pero el tercer niño se agarra a tu pierna. No eres lo suficientemente fuerte para luchar contra la corriente y nadar con los tres niños.

Si te deshaces del niño que se ha agarrado a tu pierna podrás luchar contra la corriente, pero el niño se quedará atrás ahogándose. Esto lo matará, pero os salvará a ti y a los otros dos niños.

¿Luchas contra la corriente deshaciéndote del niño, cosa que hará que ese niño se ahogue, para que tú y los otros dos niños podáis alcanzar la superficie?

**24) Impersonal-accidental**

Eres conductor/a de un autocar de guardería de excursión al zoo. De camino, un defecto en las ruedas provoca que os precipitéis desde un puente a un río bravo. Tú y tres niños estáis todavía en el vehículo que se hunde. El resto han sido arrastrados por el río. Coges a los dos niños que tienes al lado y nadas hacia la salida, pero está bloqueada por algunos asientos destrozados.

Si quitas los asientos liberarás la salida, pero obstaculizarás el pasillo y el tercer niño se quedará atrapado, ahogándose. Esto lo matará, pero os salvará a ti y a los otros dos niños.

¿Liberas la salida poniendo los asientos en el pasillo, cosa que atrapará al tercer niño, para que tú y los otros dos niños podáis alcanzar la superficie?

**(25) Personal – instrumental**

Eres doctor/a. Tienes cinco pacientes que van a morir si no reciben el trasplante inmediato de algún órgano, distinto en cada caso. Otra persona llega al hospital después de un serio accidente de coche. Su estado es crítico, pero es probable que puedas salvarla mediante una larga y complicada operación.

Si cortas a propósito su arteria carótida durante la cirugía causarás su muerte sin que nadie se entere y dispondrás de los órganos para los otros cinco pacientes. Esto matará a la víctima del accidente pero salvará a los otros cinco pacientes.

¿Obtienes los órganos cortando la arteria carótida de la víctima del accidente, para que puedas realizar los trasplantes a los otros cinco pacientes?

**26) Impersonal-Instrumental**

Eres doctor/a. Tienes cinco pacientes que van a morir si no reciben el trasplante inmediato de algún órgano, distinto en cada caso. Otra persona llega al hospital después de un serio accidente de coche. Su estado es crítico pero es probable que puedas salvarla. Durante la larga y complicada operación ves que una enfermera va a inyectarle una medicación inadecuada, letal en ese caso.

Si dejas que la enfermera inyecte la medicación errónea, la víctima del accidente morirá y dispondrás de los órganos necesarios para los otros cinco pacientes. Esto matará a la víctima, pero salvará a los cinco pacientes.

¿Obtienes los órganos dejando que la enfermera inyecte la medicación errónea a la víctima del accidente, para poder realizar los trasplantes a los otros cinco pacientes?

**27) Personal – accidental**

Eres un estibador que sujeta los cables de la grúa para descargar los barcos mercantes. Tú, con otros compañeros, sujetáis estos cables a un contenedor y montáis sobre él para asegurar que se descarga adecuadamente. De pronto ves la luz roja que indica que el cable va a fallar. Te das cuenta de que la culpa la tienen dos compañeros que se están peleando. Hacen que el contenedor se balancee peligrosamente sobre otros cinco compañeros que están en el muelle.

Si empujas a los que se pelean para separarlos evitarás que el contenedor caiga, pero uno de ellos no lleva su arnés de seguridad y caerá. Esto lo matará pero salvará a los cinco compañeros de abajo.

¿Paras el balanceo del contenedor empujando a los que se pelean, cosa que hará que el compañero sin arnés caiga, para evitar que el contenedor se desplome sobre los cinco compañeros de abajo?

**28) Impersonal – accidental**

Eres un estibador que sujeta los cables de la grúa para descargar los barcos mercantes. Tú, con otros compañeros, sujetáis estos cables a un contenedor y montáis sobre él para asegurar que se descarga adecuadamente. De pronto ves la luz roja que indica que el cable va a fallar. Si lo hace sobre la cubierta, el contenedor caerá sobre cinco tripulantes.

Si pulsas el botón de seguridad que soltará el contenedor de vuelta a la bodega, vosotros quedaréis suspendidos por vuestro arnés de seguridad, pero hay un miembro de la tripulación justo debajo. El desplome del contenedor lo matará, pero se salvarán a los cinco compañeros de la cubierta.

¿Pulsas el botón de seguridad para soltar el contenedor, cosa que hará que caiga sobre el tripulante de la bodega, para evitar que el contenedor caiga sobre los cinco tripulantes de la cubierta?

**29) Personal – accidental**

Se ha extendido una epidemia por todo el planeta, matando a miles de personas. Eres investigador/a médico/a y has desarrollado dos sustancias en el laboratorio. Por error ambas están etiquetadas como vacuna. Una de ellas ciertamente es la vacuna, que producida en masa salvará miles de vidas. La otra es una sustancia letal.

Si inyectas esas dos sustancias, sin saber cuál es cuál, a tus dos ayudantes de laboratorio, podrás identificar la vacuna. Uno de los ayudantes morirá, pero podrás salvar miles de vidas con la vacuna.

¿Identificas la vacuna inyectando las dos sustancias a tus ayudantes, cosa que hará que uno de ellos sea envenenado, para que se puedan vacunar miles de personas?

**30) Impersonal-accidental**

Se ha desatado una epidemia por todo el planeta, matando miles de personas. Eres investigador/a médico/a y has desarrollado dos sustancias en el laboratorio. Por error ambas están etiquetadas como vacuna. Una de ellas ciertamente es la vacuna, que producida en masa salvará miles de vidas. La otra es una sustancia letal.

Si permites que un enfermero inyecte esas dos sustancias, sin saber cuál es cuál, a dos pacientes, podrás identificar la vacuna. Uno de los pacientes morirá, pero podrás salvar miles de vidas con la vacuna.

¿Identificas la vacuna permitiendo que un enfermero inyecte las dos sustancias a dos pacientes, cosa que hará que uno de ellos sea envenenado, para que se puedan vacunar miles de personas?

**31) Personal – instrumental**

Un tranvía fuera de control se dirige a toda velocidad por las vías hacia cinco trabajadores, que morirán si el tranvía sigue su curso. Tú estás junto a las vías pero demasiado lejos para avisarlos. Junto a ti hay un desconocido muy grande.

Si empujas al desconocido a las vías, el tranvía descarrilará al atropellarle y no seguirá su curso hacia los trabajadores. Esto matará al desconocido, pero salvarás a los cinco trabajadores.

¿Haces descarrilar el tranvía empujando el desconocido a las vías, para que el tranvía no alcance a los cinco trabajadores?

**32) Impersonal – accidental**

Un tranvía fuera de control se dirige a toda velocidad por las vías hacia cinco trabajadores que morirán si el tranvía sigue su curso. Tú estás junto a las vías pero demasiado lejos para avisarlos. Junto a ti hay una palanca de control que puede reconducir el tranvía a otra vía donde hay un único trabajador.

Si accionas la palanca, redirigirás el tranvía hacia la vía donde hay un trabajador, y no seguirá su curso hacia los cinco trabajadores. Esto matará a ese trabajador, pero salvará a los otros cinco.

¿Rediriges las vías accionando la palanca, cosa que hará que un trabajador sea atropellado, para que el tranvía no siga su curso hacia los otros cinco trabajadores?

**33) Personal – instrumental**

Tú y un compañero de investigación habéis descubierto una nueva fuente de energía que es barata, limpia y segura. Podría acabar con la contaminación y la pobreza en el mundo. Sin embargo, tu colega quiere vender el descubrimiento, y sabes que hoy planea contactar con potenciales compradores. También sabes con seguridad que éstos intentarán usar la energía como un arma horrible, pero tu compañero no te cree.

Si envenenas a tu compañero con un veneno que se encuentra en vuestro laboratorio de forma habitual, evitarás que el descubrimiento caiga en malas manos. Esta sustancia matará a tu compañero, pero salvarás a miles de personas.

¿Impides la venta del descubrimiento envenenando a tu compañero, para que la fuente de energía no se use como arma contra miles de personas?

**34) Impersonal-Instrumental**

Tú y un compañero de investigación habéis descubierto una nueva fuente de energía que es barata, limpia y segura. Podría acabar con la contaminación y la pobreza en el mundo. Sin embargo, tu colega quiere vender el descubrimiento, y sabes que hoy planea contactar con potenciales compradores por correo electrónico. También sabes con seguridad que intentarán usar la energía como un arma horrible, pero tu compañero no te cree.

Si liberas un gas inflamable en el laboratorio, habrá una explosión en el momento en que tu compañero encienda su ordenador. Esto lo matará, pero salvarás a miles de personas.

¿Haces explotar el ordenador de tu compañero liberando el gas inflamable, para evitar que el descubrimiento se use como arma contra miles de personas?

**35) Personal – instrumental**

Eres un/a experta motorista participando en un evento de moteros. Mientras conduces delante de un grupo de 10 motoristas ves que otro motorista que va más adelante está perdiendo el control de su moto. Mientras aceleras para ponerte a su lado te das cuenta de que sufrirá una caída en cualquier momento, provocando un accidente en cadena en el que pueden morir los diez de los motoristas que te siguen.

Si empujas a este motorista fuera de la carretera se estrellará contra los árboles, pero evitarás el accidente en cadena. A vuestra velocidad, esto lo matará, pero salvarás a los diez motoristas.

¿Evitas el accidente en cadena empujando al motorista fuera de la carretera, salvando así la vida de los otros diez motoristas?

**36) Impersonal-instrumental**

Eres un/a experto/a motorista participando en un evento de moteros. Mientras conduces delante de un grupo de diez motoristas ves que otro motorista que va más adelante está perdiendo el control de su moto y cae. Puedes esquivarlo fácilmente, pero los diez motoristas detrás de ti chocarán con él y morirán en el accidente en cadena que se producirá.

Si dejas que tu moto atropelle al motorista caído, mientras saltas hábilmente a la cuneta, alertarás a tiempo a los demás. Esto matará al caído, pero salvarás a los otros diez motoristas.

¿Alertas del accidente dejando que tu moto atropelle al accidentado y saltando a la cuneta, para que los diez motoristas que te siguen también tengan tiempo de esquivarlo?

**37) Personal – instrumental**

Eres el líder de un pequeño grupo de soldados. Volvéis de una misión en territorio enemigo cuando uno de tus hombres cae en una trampa que atrapa su pierna, hiriéndole gravemente. No puedes liberarle sin que muera, pero si le abandonáis las tropas enemigas le torturarán hasta que revele la posición de un importante campamento aliado. Después atacarán dicho campamento y matarán al pelotón de diez hombres establecido allí. El soldado te suplica que no lo abandones, pero las tropas enemigas se acercan y es peligroso quedaros para ti y tus hombres porque habéis agotado la munición.

Si tú mismo apuñalas al soldado atrapado evitarás que sea torturado y revele la información que llevaría a la muerte de los diez soldados aliados. Esto le matará pero salvarás a los diez miembros del pelotón.

¿Impides que el soldado atrapado sea torturado apuñalándolo tú mismo para que no revele la localización de los diez integrantes del pelotón aliado?

**38) Impersonal – Instrumental**

Eres el líder de un pequeño grupo de soldados. Volvéis de una misión en territorio enemigo cuando uno de tus hombres cae en una trampa que atrapa su pierna, hiriéndole gravemente. No puedes liberarle sin que muera, pero si le abandonáis las tropas enemigas lo torturarán hasta que revele la posición de un importante campamento aliado. Después atacarán dicho campamento y matarán al pelotón de diez soldados establecido allí. El soldado te suplica que no lo abandones, pero las tropas enemigas se acercan y es peligroso quedaros para ti y tus hombres porque habéis agotado la munición.

Si señalas la posición del soldado atrapado con una bengala, el área será bombardeada y así evitarás que el soldado sea torturado y revele la información que llevará a la muerte del pelotón aliado. Esto lo matará, pero salvarás a los diez integrantes de este pelotón.

¿Impides que el soldado atrapado sea torturado programando el bombardeo de la zona donde está, para que no revele la localización de los diez soldados aliados?

**39) Personal – instrumental**

Eres celador/a en un hospital durante el turno de noche. Descubres que se ha vertido un producto químico en una habitación con seis pacientes. Dicho producto es altamente tóxico y si se deja en el suelo se evapora creando gas venenoso que matará a todos los pacientes de la habitación. No se puede fregar con toallas o bayetas convencionales. Sin embargo, la piel humana lo absorbe rápidamente.

Si colocas uno de los pacientes sobre el producto su piel lo absorberá y así evitarás que se evapore. Este paciente morirá, pero salvarás a los otros cinco de la habitación.

¿Evitas la evaporación tóxica colocando a un paciente sobre el producto, para que no se intoxiquen los otros cinco pacientes?

**40) Impersonal – accidental**

Eres celador/a en un hospital durante el turno de noche. Descubres que se ha vertido un producto químico en una habitación con seis pacientes. Dicho producto es altamente tóxico y si se deja en el suelo se evapora creando gas venenoso que matará a todos los pacientes de la habitación. No se puede fregar con toallas o bayetas convencionales.

Si activas el sistema de ventilación el gas venenoso será extraído de la habitación. Sin embargo, acabará en una sala del piso superior donde hay otro paciente que no puedes evacuar a tiempo. Esto matará a ese paciente, pero salvarás a los otros cinco.

¿Extraes el gas de la habitación activando el sistema de ventilación, cosa que hará que termine donde hay un paciente, para que no se intoxiquen los otros cinco pacientes?

**41) Personal – instrumental**

Estás en un bote de remos con un guía turístico en un lago de Alaska. Tres niños se han caído de su bote cerca de ti y ahora corren peligro de morir congelados. Rescatáis a los niños pero uno de ellos pesa mucho y si lo mantienes a bordo seguro que os hundiréis y todos moriréis. Ni tú ni el guía podéis abandonar el bote porque tú estás remando y el guía está reanimando uno de los niños.

Si tiras al niño más pesado por la borda seréis capaces de llegar a la orilla. Esto matará al niño, pero salvarás a los otros dos.

¿Eliminas peso de la barca tirando al niño más pesado, para que podáis alcanzar la orilla con los otros dos niños?

**42) Impersonal- accidental**

Estás en un bote de remos con un guía turístico en un lago de Alaska. Tres niños se han caído de su bote cerca de ti y ahora corren peligro de morir congelados. . Tras rescatar dos niños te das cuenta que el peso del tercero hará que el bote se hunda y todos moriréis. Ni tú ni el guía podéis abandonar el bote porque tú estás remando y el guía está reanimando a uno de los niños.

Si vuelves a la orilla dejando al tercer niño en el agua evitarás que se hunda el bote. Esto matará al niño que dejas, pero salvarás a los otros dos.

¿Evitas el hundimiento del bote dejando atrás a un niño, cosa que hará que se congele, para que podáis alcanzar la orilla con los otros dos niños?

**43) Personal – instrumental**

Trabajas en una mina. La única salida es subir en unos cubículos que cuelgan de un cable elevador. Habéis terminado la jornada y todos ascendéis a la superficie en los cubículos. Mientras esperas turno, te fijas en que el cable que los sujeta a todos está a punto de romperse.

Si vuelcas el último cubículo, el minero que lo ocupa caerá. Así reducirás suficiente peso para mantener los cubículos de otros diez mineros en el aire. Esto matará al minero del cubículo volcado, pero salvará a los otros diez.

¿Reduces la carga del cable volcando el último cubículo en el que viaja sólo un minero, para que los otros diez lleguen a la salida?

**44) Impersonal- instrumental**

Trabajas en una mina. La única salida es subir en unos cubículos que cuelgan de un cable elevador. Habéis terminado la jornada y todos ascendéis a la superficie en los cubículos. Mientras esperas turno, te fijas en que el cable que los sujeta a todos está a punto de romperse.

Si pulsas el botón de emergencia se soltará el último cubículo del cable en el que viaja sólo un minero. Así se reducirá suficiente peso para que el cable aguante los cubículos de otros diez mineros. Esto matará al minero del cubículo desenganchado, pero salvará a los otros diez.

¿Liberas carga del cable pulsando el botón que desenganchará el cubículo en el que viaja sólo un minero, para que los otros diez mineros lleguen a la salida?

**45) Personal – accidental**

Eres un/a electricista contratado/a para arreglar un problema en casa de un rico magnate del acero que está enfermo terminal por un cáncer. Recientemente ha sido anunciado que cuando muera donará su gran fortuna a un hospital infantil local. Sabes que hay 109 niños en ese hospital que podrían salvarse con ese dinero, pero que no pueden esperar mucho más porque están al borde de la muerte.

Si asfixias al magnate mientras duerme tapando su boca y nariz con tus manos enguantadas, avanzarás la donación vital para los niños. Esto matará al magnate, pero salvarás las vidas de 109 niños.

¿Adelantas la donación asfixiando a este hombre, para que la donación llegue a tiempo a los 109 niños enfermos?

**46) Impersonal-Instrumental**

Eres un/a electricista contratado/a para arreglar un problema en casa de un rico magnate del acero que está enfermo terminal por un cáncer. Recientemente ha sido anunciado que cuando muera donará su gran fortuna a un hospital infantil local. Sabes que hay 112 niños en ese hospital que podrían salvarse con ese dinero, pero que no pueden esperar mucho más porque están al borde de la muerte.

Si sobrecargas los circuitos en lugar de arreglar el problema eléctrico, el soporte vital del magnate fallará y adelantarás la donación para los niños. Esto matará al magnate, pero salvarás las vidas de 112 niños.

¿Adelantas la donación haciendo que falle el soporte vital del magnate, para que la donación llegue a tiempo a los 112 niños enfermos?

47) **Personal – instrumental**

Eres oficial en el ejército durante una guerra. Tus soldados han encontrado un espía de alto rango atrapado y herido en un edificio parcialmente derrumbado. Te han informado por radio que los soldados enemigos pretenden un ataque sorpresa sobre una de las ciudades cercanas, matando a los miles de civiles que viven allí si tú y tus soldados no lo impedís. Seguro que el espía sabe qué ciudad será atacada pero lo has interrogado y no te lo dice. No durará mucho con vida por sus heridas.

Si lo torturas, el espía revelará la información que permitirá salvar la ciudad. La tortura lo matará, pero salvarás a miles de civiles.

¿Haces que el espía revele la información torturándole, para que podáis impedir el ataque sobre miles de civiles?

**48) Impersonal-Instrumental**

Eres oficial en el ejército durante una guerra. Tus soldados han encontrado un espía de alto rango atrapado y herido en un edificio parcialmente derrumbado. Te han informado por radio que los soldados enemigos pretenden un ataque sorpresa sobre una de las ciudades cercanas, matando a los miles de civiles que viven allí si tú y tus soldados no lo impedís. Seguro que el espía sabe qué ciudad será atacada pero lo has interrogado y no te lo dice. No durará mucho con vida por sus heridas. Las ratas han empezado a morderlo y él agoniza.

Si permites que las ratas continúen esta tortura, el espía revelará la información que permitirá salvar la ciudad. Esto lo matará, pero salvarás a miles de civiles.

¿Haces que el espía te revele la información dejando que las ratas sigan torturándole, para que podáis impedir el ataque sobre miles de civiles?

**49) Personal – Others Beneficial – Inevitable (instrumental)**

Eres médico Uno de tus pacientes, que ha sido diagnosticado de SIDA, está a punto de recibir una transfusión antes de darle el alta del hospital. Te confiesa, bajo la confidencial relación médico-paciente, que tiene intención de contagiar al mayor número de personas posible, empezando esa misma tarde al salir del hospital. No tienes forma legal de pararle porque la policía no podrá arrestarle en base a una información protegida.

Si contaminas su transfusión con un veneno irrastreable evitarás que pueda infectar a otros. Esto lo matará, pero salvarás a muchos.

¿Evitas el contagio del SIDA envenenando al paciente, para que no infecte a otras personas?

**50) Impersonal – Others Beneficial – Inevitable (instrumental)**

Eres médico. Uno de tus pacientes, que tiene el SIDA, está a punto de recibir una transfusión antes de darle el alta del hospital. Te confiesa, bajo la confidencial relación médico-paciente, que tiene intención de contagiar al mayor número de personas posible, empezando esa misma tarde al salir del hospital. No tienes forma legal de pararle porque la policía no podrá arrestarle en base a una información protegida.

Si cambias su grupo sanguíneo en la base de datos del hospital recibirá la sangre equivocada y evitarás que contagie a muchas personas. Esto lo matará, pero salvarás a muchos.

¿Impides el contagio del SIDA cambiando el grupo sanguíneo del paciente, para que no infecte a otras personas?

**51) Personal – Others Beneficial – Avoidable (instrumental)**

Estás negociando con un hombre trastornado y armado en la azotea de un edificio. Abajo hay una multitud de gente en el festival de arte y música de la ciudad. Él afirma que disparará a la multitud y no puedes convencerlo de lo contrario. La multitud desconoce la amenaza y no tienes forma de avisarles. Desde esa posición, él podría matar a una docena de personas, sin contar las que se verían afectadas por el caos que se produciría al intentar ponerse a salvo.

Si corres y le empujas se precipitará desde la azotea y no podrá disparar a la multitud. Esto lo matará, pero salvarás muchas vidas.

¿Previenes el caos empujando al hombre azotea abajo, para que no dispare a la multitud?

**52) Impersonal – Others Beneficial – Avoidable (instrumental)**

Estás negociando con un hombre trastornado y armado en la azotea de un edificio. Abajo hay una multitud de gente en el festival de arte y música de la ciudad. Él afirma que disparará a la multitud y no puedes pararlo. La multitud desconoce la amenaza y no tienes forma de avisarles. Desde esa posición, él podría matar una docena de personas, sin contar las que se verían afectadas por el caos que se produciría al intentar ponerse a salvo.

Si avisas a la policía de que la negociación ha fracasado le dispararán, evitando el tiroteo sobre la multitud. Esto matará al hombre trastornado, pero se salvarán muchas vidas.

¿Evitas el caos haciendo que la policía dispare al hombre, para evitar que él acribille a la multitud?
