# Supplementary material for: Moral judgment reloaded: a moral dilemma validation study
Source: Front Psychol. 2014 Jul 1;5:607. doi: 10.3389/fpsyg.2014.00607 (PMC4077230; doi:10.3389/fpsyg.2014.00607)
Supplement: Supplementary file 11 [file DataSheet11.DOC]

Word count

**Table 3 (A): Word number of each dilemma (English) (Grey: instrumental Dilemmas)**

|  | **Personal** | | | | **Impersonal** | | | |
| --- | --- | --- | --- | --- | --- | --- | --- | --- |
|  | **Self beneficial** | | **Others beneficial** | | **Self beneficial** | | **Others beneficial** | |
| *Dilemma*  *number* | *Avoidable Death* | *Inevitable Death* | *Avoidable Death* | *Inevitable Death* | *Avoidable Death* | *Inevitable Death* | *Avoidable Death* | *Inevitable Death* |
| 1 | **117** |  |  |  |  |  |  |  |
| 2 |  |  |  |  | **133** |  |  |  |
| 3 | **146** |  |  |  |  |  |  |  |
| 4 |  |  |  |  | **139** |  |  |  |
| 5 | **154** |  |  |  |  |  |  |  |
| 6 |  |  |  |  | **156** |  |  |  |
| 7 | **138** |  |  |  |  |  |  |  |
| 8 |  |  |  |  | **153** |  |  |  |
| 9 | **165** |  |  |  |  |  |  |  |
| 10 |  |  |  |  | **171** |  |  |  |
| 11 | **163** |  |  |  |  |  |  |  |
| 12 |  |  |  |  | **168** |  |  |  |
| 13 |  | **142** |  |  |  |  |  |  |
| 14 |  |  |  |  |  | **145** |  |  |
| 15 |  | **174** |  |  |  |  |  |  |
| 16 |  |  |  |  |  | **176** |  |  |
| 17 |  | **126** |  |  |  |  |  |  |
| 18 |  |  |  |  |  | **138** |  |  |
| 19 |  | **121** |  |  |  |  |  |  |
| 20 |  |  |  |  |  | **130** |  |  |
| 21 |  | **155** |  |  |  |  |  |  |
| 22 |  |  |  |  |  | **150** |  |  |
| 23 |  | **157** |  |  |  |  |  |  |
| 24 |  |  |  |  |  | **143** |  |  |
| 25 |  |  | **112** |  |  |  |  |  |
| 26 |  |  |  |  |  |  | **124** |  |
| 27 |  |  | **169** |  |  |  |  |  |
| 28 |  |  |  |  |  |  | **168** |  |
| 29 |  |  | **122** |  |  |  |  |  |
| 30 |  |  |  |  |  |  | **121** |  |
| 31 |  |  | **106** |  |  |  |  |  |
| 32 |  |  |  |  |  |  | **130** |  |
| 33 |  |  | **138** |  |  |  |  |  |
| 34 |  |  |  |  |  |  | **141** |  |
| 35 |  |  | **138** |  |  |  |  |  |
| 36 |  |  |  |  |  |  | **132** |  |
| 37 |  |  |  | **184** |  |  |  |  |
| 38 |  |  |  |  |  |  |  | **200** |
| 39 |  |  |  | **119** |  |  |  |  |
| 40 |  |  |  |  |  |  |  | **141** |
| 41 |  |  |  | **133** |  |  |  |  |
| 42 |  |  |  |  |  |  |  | **144** |
| 43 |  |  |  | **147** |  |  |  |  |
| 44 |  |  |  |  |  |  |  | **138** |
| 45 |  |  |  | **135** |  |  |  |  |
| 46 |  |  |  |  |  |  |  | **139** |
| 47 |  |  |  | **136** |  |  |  |  |
| 48 |  |  |  |  |  |  |  | **161** |

**Table 3 (B): Word number of each dilemma (GERMAN)**

|  | **Personal** | | | | **Impersonal** | | | |
| --- | --- | --- | --- | --- | --- | --- | --- | --- |
|  | **Self beneficial** | | **Others beneficial** | | **Self beneficial** | | **Others beneficial** | |
| *Dilemma*  *number* | *Avoidable Death* | *Inevitable Death* | *Avoidable Death* | *Inevitable Death* | *Avoidable Death* | *Inevitable Death* | *Avoidable Death* | *Inevitable Death* |
| 1 | **108** |  |  |  |  |  |  |  |
| 2 |  |  |  |  | **126** |  |  |  |
| 3 | **139** |  |  |  |  |  |  |  |
| 4 |  |  |  |  | **137** |  |  |  |
| 5 | **138** |  |  |  |  |  |  |  |
| 6 |  |  |  |  | **136** |  |  |  |
| 7 | **131** |  |  |  |  |  |  |  |
| 8 |  |  |  |  | **136** |  |  |  |
| 9 | **143** |  |  |  |  |  |  |  |
| 10 |  |  |  |  | **154** |  |  |  |
| 11 | **153** |  |  |  |  |  |  |  |
| 12 |  |  |  |  | **159** |  |  |  |
| 13 |  | **128** |  |  |  |  |  |  |
| 14 |  |  |  |  |  | **138** |  |  |
| 15 |  | **160** |  |  |  |  |  |  |
| 16 |  |  |  |  |  | **165** |  |  |
| 17 |  | **120** |  |  |  |  |  |  |
| 18 |  |  |  |  |  | **126** |  |  |
| 19 |  | **119** |  |  |  |  |  |  |
| 20 |  |  |  |  |  | **130** |  |  |
| 21 |  | **117** |  |  |  |  |  |  |
| 22 |  |  |  |  |  | **124** |  |  |
| 23 |  | **155** |  |  |  |  |  |  |
| 24 |  |  |  |  |  | **142** |  |  |
| 25 |  |  | **109** |  |  |  |  |  |
| 26 |  |  |  |  |  |  | **120** |  |
| 27 |  |  | **159** |  |  |  |  |  |
| 28 |  |  |  |  |  |  | **145** |  |
| 29 |  |  | **109** |  |  |  |  |  |
| 30 |  |  |  |  |  |  | **113** |  |
| 31 |  |  | **99** |  |  |  |  |  |
| 32 |  |  |  |  |  |  | **122** |  |
| 33 |  |  | **124** |  |  |  |  |  |
| 34 |  |  |  |  |  |  | **125** |  |
| 35 |  |  | **124** |  |  |  |  |  |
| 36 |  |  |  |  |  |  | **127** |  |
| 37 |  |  |  | **182** |  |  |  |  |
| 38 |  |  |  |  |  |  |  | **191** |
| 39 |  |  |  | **115** |  |  |  |  |
| 40 |  |  |  |  |  |  |  | **134** |
| 41 |  |  |  | **151** |  |  |  |  |
| 42 |  |  |  |  |  |  |  | **152** |
| 43 |  |  |  | **131** |  |  |  |  |
| 44 |  |  |  |  |  |  |  | **142** |
| 45 |  |  |  | **124** |  |  |  |  |
| 46 |  |  |  |  |  |  |  | **132** |
| 47 |  |  |  | **131** |  |  |  |  |
| 48 |  |  |  |  |  |  |  | **149** |

**Table 3 (C): Word number of each dilemma (CATALAN)**

|  | **Personal** | | | | **Impersonal** | | | |
| --- | --- | --- | --- | --- | --- | --- | --- | --- |
|  | **Self beneficial** | | **Others beneficial** | | **Self beneficial** | | **Others beneficial** | |
| *Dilemma*  *number* | *Avoidable Death* | *Inevitable Death* | *Avoidable Death* | *Inevitable Death* | *Avoidable Death* | *Inevitable Death* | *Avoidable Death* | *Inevitable Death* |
| 1 | **114** |  |  |  |  |  |  |  |
| 2 |  |  |  |  | **124** |  |  |  |
| 3 | **130** |  |  |  |  |  |  |  |
| 4 |  |  |  |  | **133** |  |  |  |
| 5 | **133** |  |  |  |  |  |  |  |
| 6 |  |  |  |  | **135** |  |  |  |
| 7 | **133** |  |  |  |  |  |  |  |
| 8 |  |  |  |  | **146** |  |  |  |
| 9 | **143** |  |  |  |  |  |  |  |
| 10 |  |  |  |  | **160** |  |  |  |
| 11 | **141** |  |  |  |  |  |  |  |
| 12 |  |  |  |  | **144** |  |  |  |
| 13 |  | **116** |  |  |  |  |  |  |
| 14 |  |  |  |  |  | **129** |  |  |
| 15 |  | **176** |  |  |  |  |  |  |
| 16 |  |  |  |  |  | **167** |  |  |
| 17 |  | **120** |  |  |  |  |  |  |
| 18 |  |  |  |  |  | **124** |  |  |
| 19 |  | **112** |  |  |  |  |  |  |
| 20 |  |  |  |  |  | **116** |  |  |
| 21 |  | **106** |  |  |  |  |  |  |
| 22 |  |  |  |  |  | **116** |  |  |
| 23 |  | **146** |  |  |  |  |  |  |
| 24 |  |  |  |  |  | **124** |  |  |
| 25 |  |  | **111** |  |  |  |  |  |
| 26 |  |  |  |  |  |  | **120** |  |
| 27 |  |  | **140** |  |  |  |  |  |
| 28 |  |  |  |  |  |  | **143** |  |
| 29 |  |  | **110** |  |  |  |  |  |
| 30 |  |  |  |  |  |  | **115** |  |
| 31 |  |  | **97** |  |  |  |  |  |
| 32 |  |  |  |  |  |  | **126** |  |
| 33 |  |  | **125** |  |  |  |  |  |
| 34 |  |  |  |  |  |  | **125** |  |
| 35 |  |  | **118** |  |  |  |  |  |
| 36 |  |  |  |  |  |  | **121** |  |
| 37 |  |  |  | **158** |  |  |  |  |
| 38 |  |  |  |  |  |  |  | **170** |
| 39 |  |  |  | **110** |  |  |  |  |
| 40 |  |  |  |  |  |  |  | **133** |
| 41 |  |  |  | **115** |  |  |  |  |
| 42 |  |  |  |  |  |  |  | **126** |
| 43 |  |  |  | **113** |  |  |  |  |
| 44 |  |  |  |  |  |  |  | **119** |
| 45 |  |  |  | **106** |  |  |  |  |
| 46 |  |  |  |  |  |  |  | **108** |
| 47 |  |  |  | **124** |  |  |  |  |
| 48 |  |  |  |  |  |  |  | **141** |

**Table 3 (D): Word number of each dilemma (SPANISH)**

|  | **Personal** | | | | **Impersonal** | | | |
| --- | --- | --- | --- | --- | --- | --- | --- | --- |
|  | **Self beneficial** | | **Others beneficial** | | **Self beneficial** | | **Others beneficial** | |
| *Dilemma*  *number* | *Avoidable Death* | *Inevitable Death* | *Avoidable Death* | *Inevitable Death* | *Avoidable Death* | *Inevitable Death* | *Avoidable Death* | *Inevitable Death* |
| 1 | **114** |  |  |  |  |  |  |  |
| 2 |  |  |  |  | **126** |  |  |  |
| 3 | **130** |  |  |  |  |  |  |  |
| 4 |  |  |  |  | **135** |  |  |  |
| 5 | **134** |  |  |  |  |  |  |  |
| 6 |  |  |  |  | **139** |  |  |  |
| 7 | **134** |  |  |  |  |  |  |  |
| 8 |  |  |  |  | **148** |  |  |  |
| 9 | **143** |  |  |  |  |  |  |  |
| 10 |  |  |  |  | **161** |  |  |  |
| 11 | **145** |  |  |  |  |  |  |  |
| 12 |  |  |  |  | **150** |  |  |  |
| 13 |  | **132** |  |  |  |  |  |  |
| 14 |  |  |  |  |  | **141** |  |  |
| 15 |  | **160** |  |  |  |  |  |  |
| 16 |  |  |  |  |  | **162** |  |  |
| 17 |  | **125** |  |  |  |  |  |  |
| 18 |  |  |  |  |  | **131** |  |  |
| 19 |  | **116** |  |  |  |  |  |  |
| 20 |  |  |  |  |  | **127** |  |  |
| 21 |  | **114** |  |  |  |  |  |  |
| 22 |  |  |  |  |  | **119** |  |  |
| 23 |  | **153** |  |  |  |  |  |  |
| 24 |  |  |  |  |  | **130** |  |  |
| 25 |  |  | **115** |  |  |  |  |  |
| 26 |  |  |  |  |  |  | **126** |  |
| 27 |  |  | **150** |  |  |  |  |  |
| 28 |  |  |  |  |  |  | **142** |  |
| 29 |  |  | **114** |  |  |  |  |  |
| 30 |  |  |  |  |  |  | **118** |  |
| 31 |  |  | **93** |  |  |  |  |  |
| 32 |  |  |  |  |  |  | **117** |  |
| 33 |  |  | **128** |  |  |  |  |  |
| 34 |  |  |  |  |  |  | **123** |  |
| 35 |  |  | **123** |  |  |  |  |  |
| 36 |  |  |  |  |  |  | **119** |  |
| 37 |  |  |  | **156** |  |  |  |  |
| 38 |  |  |  |  |  |  |  | **169** |
| 39 |  |  |  | **118** |  |  |  |  |
| 40 |  |  |  |  |  |  |  | **132** |
| 41 |  |  |  | **125** |  |  |  |  |
| 42 |  |  |  |  |  |  |  | **135** |
| 43 |  |  |  | **114** |  |  |  |  |
| 44 |  |  |  |  |  |  |  | **127** |
| 45 |  |  |  | **119** |  |  |  |  |
| 46 |  |  |  |  |  |  |  | **125** |
| 47 |  |  |  | **128** |  |  |  |  |
| 48 |  |  |  |  |  |  |  | **147** |

**Table 3 (E): Word number of each dilemma (FRENCH)**

|  | **Personal** | | | | **Impersonal** | | | |
| --- | --- | --- | --- | --- | --- | --- | --- | --- |
|  | **Self beneficial** | | **Others beneficial** | | **Self beneficial** | | **Others beneficial** | |
| *Dilemma*  *number* | *Avoidable Death* | *Inevitable Death* | *Avoidable Death* | *Inevitable Death* | *Avoidable Death* | *Inevitable Death* | *Avoidable Death* | *Inevitable Death* |
| 1 | **116** |  |  |  |  |  |  |  |
| 2 |  |  |  |  | **131** |  |  |  |
| 3 | **138** |  |  |  |  |  |  |  |
| 4 |  |  |  |  | **127** |  |  |  |
| 5 | **134** |  |  |  |  |  |  |  |
| 6 |  |  |  |  | **146** |  |  |  |
| 7 | **140** |  |  |  |  |  |  |  |
| 8 |  |  |  |  | **144** |  |  |  |
| 9 | **144** |  |  |  |  |  |  |  |
| 10 |  |  |  |  | **156** |  |  |  |
| 11 | **137** |  |  |  |  |  |  |  |
| 12 |  |  |  |  | **144** |  |  |  |
| 13 |  | **136** |  |  |  |  |  |  |
| 14 |  |  |  |  |  | **138** |  |  |
| 15 |  | **158** |  |  |  |  |  |  |
| 16 |  |  |  |  |  | **164** |  |  |
| 17 |  | **119** |  |  |  |  |  |  |
| 18 |  |  |  |  |  | **137** |  |  |
| 19 |  | **129** |  |  |  |  |  |  |
| 20 |  |  |  |  |  | **134** |  |  |
| 21 |  | **126** |  |  |  |  |  |  |
| 22 |  |  |  |  |  | **134** |  |  |
| 23 |  | **146** |  |  |  |  |  |  |
| 24 |  |  |  |  |  | **137** |  |  |
| 25 |  |  | **113** |  |  |  |  |  |
| 26 |  |  |  |  |  |  | **111** |  |
| 27 |  |  | **174** |  |  |  |  |  |
| 28 |  |  |  |  |  |  | **170** |  |
| 29 |  |  | **116** |  |  |  |  |  |
| 30 |  |  |  |  |  |  | **119** |  |
| 31 |  |  | **89** |  |  |  |  |  |
| 32 |  |  |  |  |  |  | **118** |  |
| 33 |  |  | **147** |  |  |  |  |  |
| 34 |  |  |  |  |  |  | **152** |  |
| 35 |  |  | **125** |  |  |  |  |  |
| 36 |  |  |  |  |  |  | **126** |  |
| 37 |  |  |  | **169** |  |  |  |  |
| 38 |  |  |  |  |  |  |  | **177** |
| 39 |  |  |  | **124** |  |  |  |  |
| 40 |  |  |  |  |  |  |  | **143** |
| 41 |  |  |  | **153** |  |  |  |  |
| 42 |  |  |  |  |  |  |  | **171** |
| 43 |  |  |  | **151** |  |  |  |  |
| 44 |  |  |  |  |  |  |  | **168** |
| 45 |  |  |  | **128** |  |  |  |  |
| 46 |  |  |  |  |  |  |  | **139** |
| 47 |  |  |  | **135** |  |  |  |  |
| 48 |  |  |  |  |  |  |  | **153** |

**Table 3 (F): Word number of each dilemma (DANISH)**

|  | **Personal** | | | | **Impersonal** | | | |
| --- | --- | --- | --- | --- | --- | --- | --- | --- |
|  | **Self beneficial** | | **Others beneficial** | | **Self beneficial** | | **Others beneficial** | |
| *Dilemma*  *number* | *Avoidable Death* | *Inevitable Death* | *Avoidable Death* | *Inevitable Death* | *Avoidable Death* | *Inevitable Death* | *Avoidable Death* | *Inevitable Death* |
| 1 | **122** |  |  |  |  |  |  |  |
| 2 |  |  |  |  | **128** |  |  |  |
| 3 | **131** |  |  |  |  |  |  |  |
| 4 |  |  |  |  | **126** |  |  |  |
| 5 | **128** |  |  |  |  |  |  |  |
| 6 |  |  |  |  | **136** |  |  |  |
| 7 | **127** |  |  |  |  |  |  |  |
| 8 |  |  |  |  | **134** |  |  |  |
| 9 | **136** |  |  |  |  |  |  |  |
| 10 |  |  |  |  | **141** |  |  |  |
| 11 | **143** |  |  |  |  |  |  |  |
| 12 |  |  |  |  | **143** |  |  |  |
| 13 |  | **123** |  |  |  |  |  |  |
| 14 |  |  |  |  |  | **131** |  |  |
| 15 |  | **152** |  |  |  |  |  |  |
| 16 |  |  |  |  |  | **159** |  |  |
| 17 |  | **100** |  |  |  |  |  |  |
| 18 |  |  |  |  |  | **117** |  |  |
| 19 |  | **122** |  |  |  |  |  |  |
| 20 |  |  |  |  |  | **121** |  |  |
| 21 |  | **120** |  |  |  |  |  |  |
| 22 |  |  |  |  |  | **127** |  |  |
| 23 |  | **147** |  |  |  |  |  |  |
| 24 |  |  |  |  |  | **139** |  |  |
| 25 |  |  | **109** |  |  |  |  |  |
| 26 |  |  |  |  |  |  | **118** |  |
| 27 |  |  | **158** |  |  |  |  |  |
| 28 |  |  |  |  |  |  | **153** |  |
| 29 |  |  | **101** |  |  |  |  |  |
| 30 |  |  |  |  |  |  | **108** |  |
| 31 |  |  | **98** |  |  |  |  |  |
| 32 |  |  |  |  |  |  | **110** |  |
| 33 |  |  | **126** |  |  |  |  |  |
| 34 |  |  |  |  |  |  | **135** |  |
| 35 |  |  | **128** |  |  |  |  |  |
| 36 |  |  |  |  |  |  | **135** |  |
| 37 |  |  |  | **164** |  |  |  |  |
| 38 |  |  |  |  |  |  |  | **174** |
| 39 |  |  |  | **111** |  |  |  |  |
| 40 |  |  |  |  |  |  |  | **129** |
| 41 |  |  |  | **147** |  |  |  |  |
| 42 |  |  |  |  |  |  |  | **168** |
| 43 |  |  |  | **131** |  |  |  |  |
| 44 |  |  |  |  |  |  |  | **137** |
| 45 |  |  |  | **130** |  |  |  |  |
| 46 |  |  |  |  |  |  |  | **131** |
| 47 |  |  |  | **130** |  |  |  |  |
| 48 |  |  |  |  |  |  |  | **151** |
